# Weak and inconsistent associations between birch-feeding insects and pollution levels around two industrial towns during substantial emission decline (1988–2025)

**DOI:** 10.1007/s10661-026-15971-1

**Published:** 2026-10-07

**Authors:** Mikhail V. Kozlov, Vitali Zverev

**Affiliations:** Department of Biology, University of Turku, Turku, 20014 Finland

**Keywords:** Aluminium smelter, Insect diversity, Natural recovery, Pollution decline, Population density, Pulp and paper mill

## Abstract

Industrial emissions have historically altered terrestrial ecosystems, but their long-term effects on insect herbivores remain insufficiently understood, particularly during ecosystem recovery following emission decline. We analysed a dataset on the densities of 24 Recognisable Taxonomic Units (RTUs) of birch-feeding insects collected from 1988 to 2025 around two industrial towns in northwestern Russia previously identified as pollution hotspots. Volkhov originally hosted an aluminium smelter, and Syasstroy was home to a pulp and paper mill. Industrial emissions from these two towns formed pollution gradients that differed in pollutant composition but were originally comparable in their environmental effects, as indicated by Norway spruce vigour. A small subset of RTUs exhibited higher densities near both polluters during the high-emission years (1988–1996), and these patterns persisted until 2025, despite the subsequent reduction in emissions and apparent ecosystem recovery. Insect herbivore diversity was independent of pollution level during both high- and low-emission periods. At the same time, birch leaf quality for autumnal moth, *Epirrita autumnata* increased near polluters, whereas foliar damage by insects decreased. From 1988 to 2025, the densities of monitored insects showed no significant temporal trend in sites with any pollution levels. Our results demonstrate that moderate industrial pollution has limited and taxon- or guild-specific effects on insect herbivore communities, which often show inconsistencies between polluters and among the study years. Furthermore, substantial emission reductions do not produce long-term trends in the abundance of birch-feeding insects. These findings challenge the putative role of industrial pollution as a major driver of insect decline.

**Supplementary Information:**

The online version contains supplementary material available at https://doi.org/10.1007/s10661-026-15971-1.

## Introduction

Industrial emissions have long been recognised as major drivers of ecosystem degradation, as they alter soil chemistry, biodiversity, and ecosystem functioning (Euler, 1939; Freedman & Hutchinson, 1980; Haywood, 1910; Kozlov et al., 2009). However, because early studies of the effects of industrial pollution on terrestrial biota focused primarily on lifeless industrial barrens, these studies created the impression that pollution caused broadly uniform changes across ecosystems (Kozlov & Zvereva, 2011). Consequently, several attempts were made to identify general rules that govern ecosystem responses to abiotic stress (Odum, 1985; Rapport et al., 1985). However, subsequent research demonstrated that biotic responses to pollution vary depending on the characteristics of the polluter (type and amount of emissions, duration of exposure), the affected organisms (trophic group, life-history traits), the level of biological organisation examined (organism, population, community), and the surrounding environment (biome, climate). These context dependencies have complicated the formulation of reliable predictions of the ecosystem-level effects of airborne industrial pollutants (Kozlov & Zvereva, 2011; Kozlov et al., 2009).

The environmental impacts of large industrial polluters appear to have lessened over recent decades, driven by regulatory changes, industrial decline, and technological modernisation (Pacyna et al., 2009; Turnock et al., 2016). This reduction in pollutant levels has created an urgent need to explore how natural systems respond to reduced pollution pressure, particularly with respect to unassisted ecosystem recovery. However, data addressing this problem remain scarce (Vorobeichik, 2022), raising the question of whether the rates and trajectories of ecosystem recovery can be predicted by collating the results from previously published meta-analyses of pollution impacts on biota and integrating them with large-scale, long-term datasets that span periods of substantial emission decline (Kozlov et al., 2017a).

One source of data for this type of analysis is provided by herbivorous insects, which are often regarded as reliable indicators of environmental disturbance and recovery (Chowdhury et al., 2023; Zvereva & Kozlov, 2010), owing to their important roles in ecosystem processes (e.g. plant community dynamics and nutrient cycling: Kozlov & Zvereva, 2017; Tamburini et al., 2018). Historically, this focus has been reinforced by the widespread assumption that pollution promotes outbreaks of herbivorous insects, particularly forest pests (Baltensweiler, 1985; Führer, 1985). A meta-analysis by Zvereva and Kozlov (2010) supported this view by reporting elevated herbivore abundances near industrial sites; however, these authors also suggested that the observed pattern may partly reflect methodological and publication biases, including selective reporting and preferential attention to expected outcomes. Consequently, more recent studies based on systematically collected data have challenged the notion that pollution consistently increases herbivory. Instead, they have reported either no increase or even decreases in herbivorous insect densities near pollution sources (Belskaya & Zamshina, 2024; Kozlov et al., 2009, 2017a).

Consistent with these findings, the performance of major pest invertebrates and other herbivores appears largely unaffected by air pollution (Ryalls et al., 2024). However, because the strength of biotic responses declines with decreasing pollution level (Kozlov & Zvereva, 2011; Zvereva et al., 2008), the apparent disappearance of significant pollution effects on herbivory described in recent studies may instead reflect ecosystem recovery following reductions in emissions (Belskaya, 2018). Here, we test this hypothesis using long-term observations of birch-feeding insects from consistent networks of sites surrounding two industrial towns in northwestern Russia: Volkhov, which initially hosted an aluminium smelter that was later replaced by a fertiliser plant, and Syasstroy, where a pulp and paper mill is located.

Based on the results of an earlier comparative study (Kozlov et al., 2009), together with meta-analyses aimed at identifying general patterns of ecosystem responses to pollution (Kozlov & Zvereva, 2011; Zvereva & Kozlov, 2010), we formulated the following predictions: (1) the densities of different leaf-eating insect taxa respond to pollution in divergent ways, ranging from increases to decreases; (2) during the period of relatively high emissions (1988–1996), the overall abundance of herbivorous insects increases, whereas their diversity decreases with their proximity to polluters; (3) these effects are stronger near Syasstroy, which is characterised by larger SO_2_ emissions, than near Volkhov, which is characterised primarily by fluoride emissions; (4) during the period of relatively low emissions (1997–2025), these patterns weaken or disappear completely; and (5) ecosystem recovery leads to shifts in the herbivore community composition because species belonging to different feeding guilds respond differently to declining pollution pressure.

The recent surge of interest in global insect decline (van Klink et al., 2020; Vidal et al., 2025) further prompted us to evaluate two additional predictions: (6) both the abundance and diversity of herbivorous insects in unpolluted and slightly polluted sites show declines over the 38-year observation period, and (7) insect abundance declines more rapidly over time in sites that were moderately or heavily polluted at the beginning of the study than in less polluted sites, whereas insect diversity declines more slowly in the more heavily polluted than in less polluted sites. Importantly, testing these predictions (6) and (7) using our dataset minimises potential observer bias, because most of these data were collected before global insect decline became widely recognised as an ecologically important phenomenon requiring urgent scientific attention.

## Materials and methods

### Study region

**Fig. 1:**
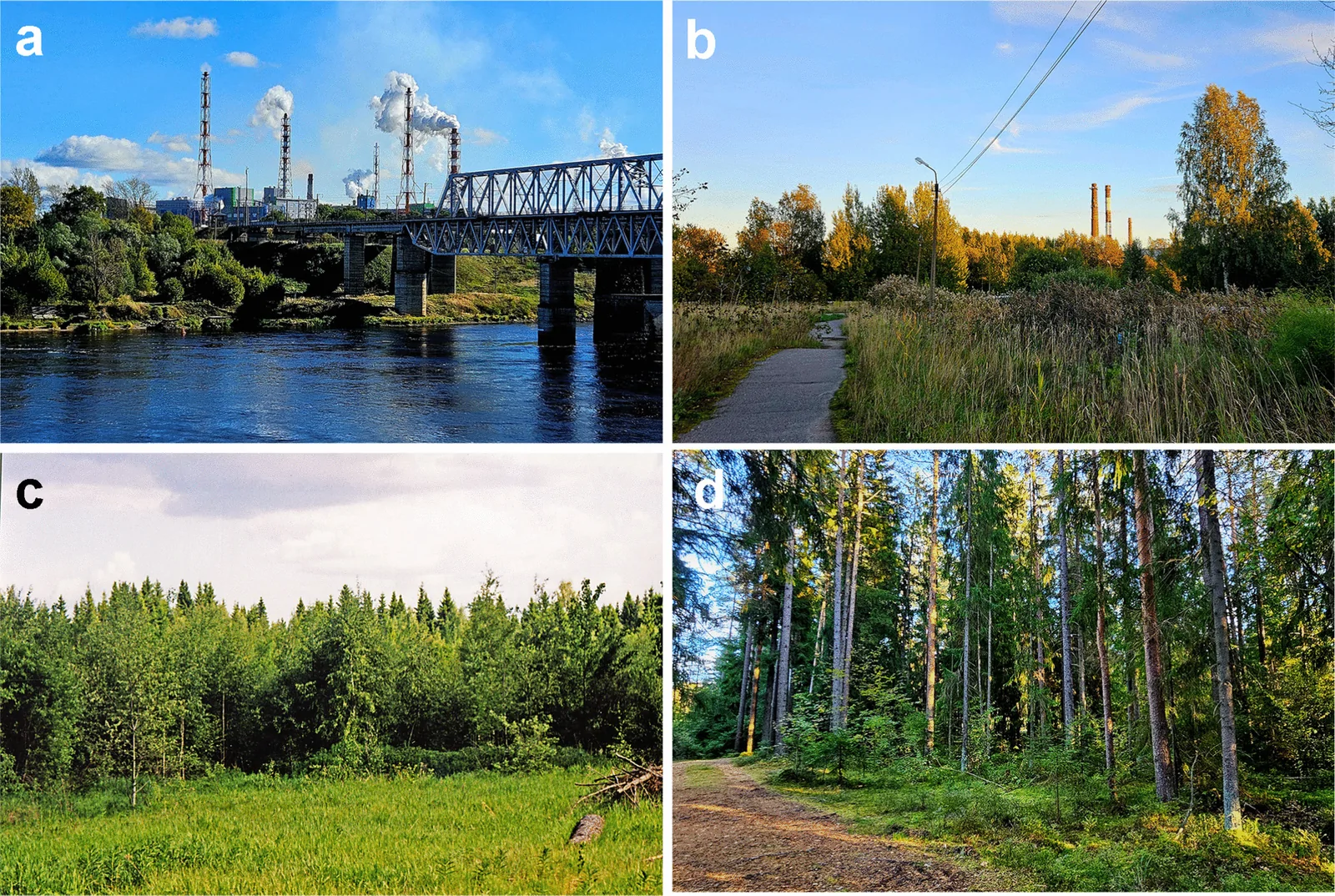
Industrially polluted (**a**, **b**) and unpolluted forest sites (**c**, **d**): **a** the Volkhov chemical cluster of the PhosAgro Group; **b** chimneys of the Syassky pulp and paper mill; **c** site located 13.9 km north of Volkhov; **d** site located 11.7 km west of Syasstroy. Photo: V. Zverev

The industrial settlements of Volkhov (59° 53′ N, 32° 20′ E; Fig. 1a) and Syasstroy (60° 08′ N, 32° 34′ E; Fig. 1b) are located in the eastern part of the Leningrad Oblast, northwestern Russia, within the southern boreal forest zone. The topography is mostly flat, with numerous rivers, streams, and wetlands, including peat bogs, contributing to high landscape heterogeneity and ecological diversity. The natural vegetation is dominated by mixed coniferous–deciduous forests, where Norway spruce (*Picea abies*), Scots pine (*Pinus sylvestris*), silver and downy birches (*Betula pendula*, *B. pubescens*), and aspen (*Populus tremula*) are the most common tree species. Understory vegetation includes shrubs such as *Vaccinium myrtillus*, *V. vitis-idaea*, and *Empetrum nigrum*, as well as mosses and ferns, particularly in moist or shaded areas (Fig. 1c, d). The climate is humid continental, with cool summers and cold, snowy winters. Mean annual temperature is around + 4 to + 6 °C, with January averaging − 6.2 °C and July + 18.4 °C. Annual precipitation ranges from 600 to 700 mm, evenly distributed across seasons. During the growing season, wind directions in the study region are broadly distributed, with no strongly prevailing direction (Koroleva et al., 2020).

### Industrial history

The town of Volkhov (current population 37,300) developed in the early twentieth century from a small village. Its growth was enabled by the Volkhov hydroelectric power station, launched in 1926. The availability of cheap electricity was critical for the development of the Volkhov aluminium smelter. Launched in 1932, the smelter was a key industrial asset, producing 5000 metric tonnes (t) of aluminium annually in the 1930 s and 21,000–24,000 t in the 2000 s until its closure in 2012 due to outdated technology and environmental concerns. On its grounds, a modern chemical enterprise, Metachem, was established in 2003. Since 2012, Metachem has operated as part of the PhosAgro Group, manufacturing fertilisers and various acids (https://en.wikipedia.org/wiki/Volkhov).

Syasstroy (current population 12,200), located approximately 28 km northeast of Volkhov, was founded in 1926 as a workers’ settlement for the Syassky pulp and paper mill, which began operation in 1928. This mill remains the town’s dominant employer and has shaped its development and identity. Originally focused on producing newsprint and industrial paper, the plant has expanded over the decades to include a variety of pulp, paper, and cardboard products. Today, it is one of the largest enterprises of its kind in northwestern Russia, with ongoing modernisation and infrastructure upgrades (https://en.wikipedia.org/wiki/Syasstroy).

### Aerial emissions, extent of contaminated territory, and location of study sites

Industrial enterprises in both towns emit solid particles (dust), SO_2_, and NO_x_ into the atmosphere. Additionally, industries in Volkhov release fluoride-containing compounds in both particulate and gaseous forms. In Volkhov, SO_2_ emissions decreased by a factor of four from 1988 to 2021, while fluorine emissions dropped 15-fold in the early 1990 s and have since remained relatively stable. Emissions of dust fluctuated, but were about 50% lower in the 2020 s than in the late 1980s. NO_x_ emissions declined fourfold between the 1980 s and 2000 s, but later increased in some years to approximately three times higher in the 2020 s as in the 1980 s (Table S1). In Syasstroy, emissions of dust and SO_2_ plummeted 100-fold in the 1990 s following abrupt production decline (Melnikova, 2001) and have remained low since then. NO_x_ emissions declined eightfold over the same period but later rose again in 2001, nearing (though not fully reaching) the levels observed in the late 1980 s (Table S1).

**Fig. 2:**
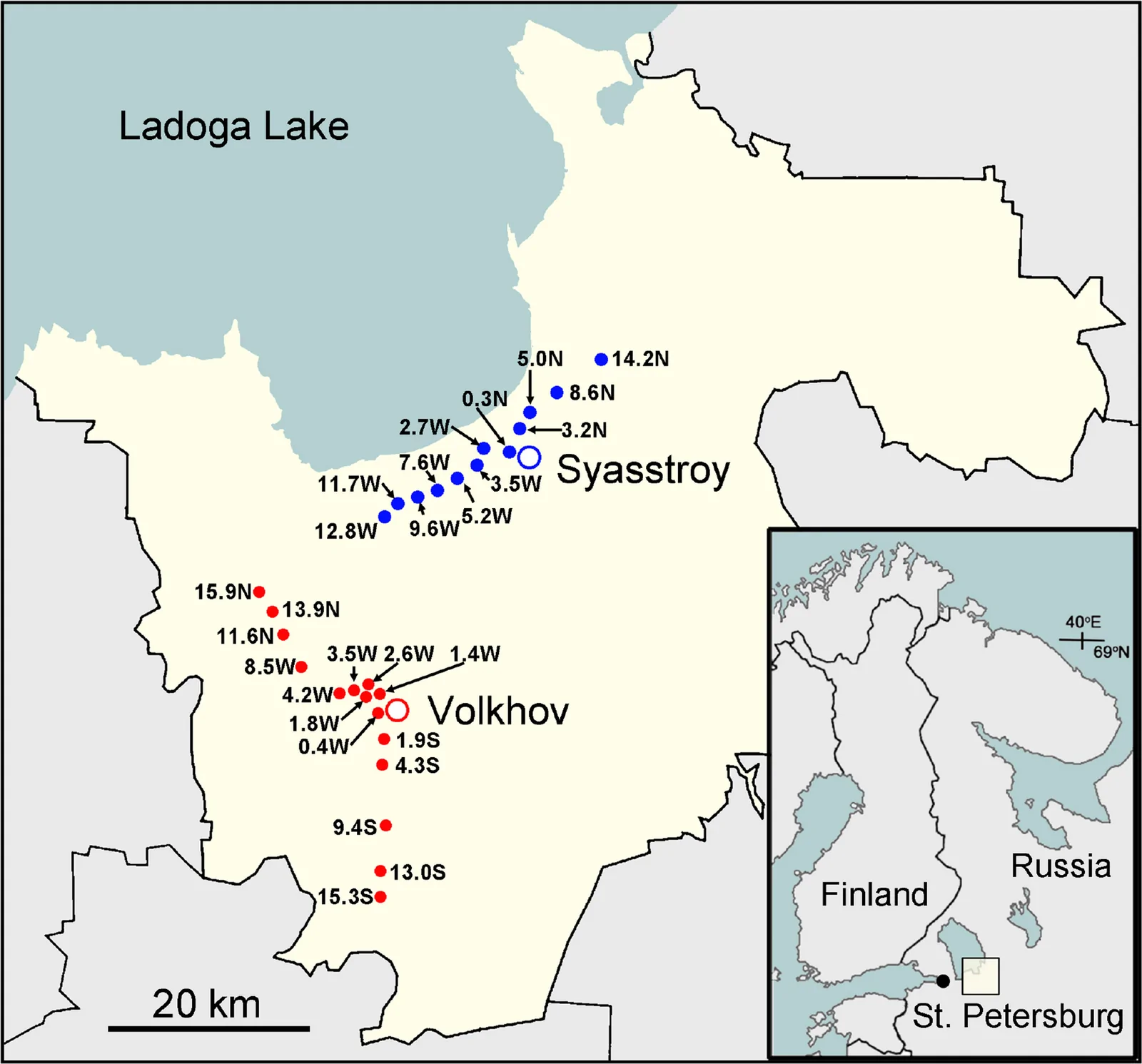
Location of study sites around Volkhov (red symbols) and Syasstroy (blue symbols). Open circles indicate polluters. Site codes denote distance (km) and direction (north, west, or south) from the respective polluters. Coordinates of study sites are provided in (Table S2). Inset: location of the study area (square) in Northern Europe

At the start of our study (in 1988), the features of pollution dispersal near Volkhov and Syasstroy were entirely unknown. Based on emission levels and comparisons with other industrial enterprises (Kozlov, 1990), we initially estimated the extent of the affected areas as approximately 10 km from the pollution source. Therefore, control (unpolluted) sites were selected 12–16 km away from each polluter (Fig. 2). Further on, sites located 6–12 km from the polluters are termed slightly polluted and contrasted with moderately to heavily polluted sites located 0.3–5.2 km from the polluters.

Study sites were selected in two approximately opposite directions from each polluter in such a way that during the growing season, winds from the polluter to our study sites occurred at approximately similar frequencies (Volkhov, 12% northern vs 9% southeastern; Syasstroy, 14% southwestern vs 10% northeastern; Koroleva et al., 2020). Where possible, we selected study sites in natural forests; however, no forest patches persisted near the polluters. Therefore, the plots nearest to the emission sources were located in parks, rural settlements, or roadside urban habitats. Because pollutant concentrations decrease hyperbolically with distance from the source (Kozlov et al., 2009), we placed our study plots more densely near the polluters and at wider intervals farther away (Table S2).

### Study system

Downy birch (*B. pubescens*) is one of the dominant deciduous tree species in the southern taiga forests of northern Europe and western Russia. It typically grows in moist soils, forming pure stands or mixed stands with conifers, such as Norway spruce and Scots pine. Downy birch is well adapted to cold climates and short growing seasons and plays key roles in forest succession, soil formation and the maintenance of biodiversity (Atkinson, 1992).

Birch foliage supports a diverse community of insect herbivores (Atkinson, 1992; Kozlov et al., 2013, 2017a), including externally feeding defoliators (Fig. S1a, j), leafminers (Fig. S1b, c, e–g, k–o), and leafrollers (Fig. S1h, i). Common defoliators include geometrid moths and sawflies, which can cause extensive leaf loss during outbreak years. Leafminers are mainly moths from the families Eriocraniidae, Stigmellidae, and Gracillariidae, as well as sawflies (Tenthredinidae). Leaf rollers (moths and leaf-rolling weevils) fold or roll leaves or tie them together to form characteristic nests. In our study region, we recorded more than 60 species of insects whose larvae feed on birch leaves (Table 1).

**Table 1:** Insects that were recorded feeding on foliage of *Betula pubescens* in surroundings of Volkhov and Syasstroy, northwestern Russia, from 1988–2025

Herbivore species/group (RTU^a^)	Species comprising a group	Order and family	Feeding guild	Observation unit	Number of units
*Achlya flavicornis* (L.)		Lepidoptera: Drepanidae	Defoliator	Larva	2
*Agromyza alnibetulae* (Hendel)		Diptera: Agromyzidae	Leafminer	Mine	208
*Apoderus coryli* (L.)		Coleoptera: Attelabidae	Leaf roller	Roll	1
*Bucculatrix demaryella* (Dup.)		Lepidoptera: Bucculatricidae	Leafminer	Mine	9
*Coleophora* spp.	*Coleophora serratella* (L.), *C. milvipennis* Z., and other species	Lepidoptera: Coleophoridae	Leafminer	Mine	8650
*Colocasia coryli* (L.)		Lepidoptera: Noctuidae	Defoliator	Larva	18
*Deporaus betulae* (L.)		Coleoptera: Rhynchitidae	Leaf roller	Roll	6636
Drepanidae	*Drepana falcataria* (L.), *Falcaria lacertinaria* (L.)	Lepidoptera: Drepanidae	Defoliator	Larva	12
*Ectoedemia minimella* (Zett.)		Lepidoptera: Nepticulidae	Leafminer	Mine	199
*Epirrita autumnata* (Bkh.)		Lepidoptera: Geometridae	Defoliator	Larva	76
*Eriocrania sparrmannella* (Bosc)		Lepidoptera: Eriocraniidae	Leafminer	Mine	33
*Eriocrania* spp.	*Eriocrania semipurpurella* (Stph.), *E. sangii* (Wood), *E. cicatricella* (Zett.)	Lepidoptera: Eriocraniidae	Leafminer	Mine	42281
Geometridae	*Cabera pusaria* (L.), *Ectropis crepuscularia* (Den. and Schiff.), *Erannis defoliaria* (Cl.), *Jodis lactearia* (L.), *Lycia hirtaria* (Cl.), *Selenia dentaria* (F.), and other species	Lepidoptera: Geometridae	Defoliator	Larva	63
*Incurvaria pectinea* (Hw.)		Lepidoptera: Incurvariidae	Leafminer	Mine	1428
Leaf rollers	*Apotomis betuletana* (Hw.), *A. sororculana* (Zett.), *Choristoneura diversana* (Hb.), *Epinotia solandriana* (L.), *Eulia ministrana* (L.), *Gypsonoma dealbana* (Fröl.), *Metendothenia atropunctana* (Zett.), *Syndemis musculana* (Hb.), *Caloptilia populetorum* (Zeller), *C. betulicola* (Hering), *Ypsolopha parenthesella* (L.), *Carpatolechia proximella* (Hb.), *Hypatima rhomboidella* (L.), *Pseudotelphusa paripunctella* (Thunb.), *Swammerdamia caesiella* (Hb.), and other species	Lepidoptera: Tortricidae Lepidoptera: Gracillariidae Lepidoptera: Ypsolophidae Lepidoptera: Gelechiidae Lepidoptera: Yponomeutidae	Leaf roller	Larval shelter	4236
Leaf-chewing sawflies	*Arge* sp., *Cimbex femoratus* (L.), *Cladius* sp., *Dineura* sp., *Empria candidata* (Fall.), *Euura* sp., *Hemichroa australis* (Serv.), *Nematinus* sp., *Nematus latipes* Villaret, and other species	Hymenoptera: Argidae Hymenoptera: Cimbicidae Hymenoptera: Tenthredinidae	Defoliator	Larva	182
Leafmining sawflies	*Fenusa pumila* (Leach), *Fenusella nana* (Klug), *Scolioneura betuleti* (Klug)	Hymenoptera: Tenthredinidae	Leafminer	Mine	1786
*Notodonta dromedarius* (L.)		Lepidoptera: Notodontidae	Defoliator	Larva	11
*Orchestes* spp.	*Orchestes calceatus* (Germar), *O. rusci* (Herbst), *O. testaceus* (Müller)	Coleoptera: Curculionidae	Leafminer	Mine	40
*Parornix* spp.	*Parornix betulae* (Stt.), *P. loganella* (Stt.), *P. scoticella* (Stt.)	Lepidoptera: Gracillariidae	Leafminer	Mine and folded leaf	2770
*Phyllonorycter corylifoliella* (Hbn.)		Lepidoptera: Gracillariidae	Leafminer	Mine	14
*Phylloporia bistrigella* (Hw.)		Lepidoptera: Incurvariidae	Leafminer	Mine	90
Psychidae	*Sterrhopterix standfussi* (Wck.), *Psyche casta* (Pallas)	Lepidoptera: Psychidae	Defoliator	Larva	8
*Stigmella* spp.	*Stigmella betulicola* (Stt.), *S. confusella* (Wood), *S. continuella* (Stt.), *S. lapponica* (Wck.), *S. luteella* (Stt.)	Lepidoptera: Nepticulidae	Leafminer	Mine	1720

^a^*RTU* Recognisable Taxonomic Unit

### Patterns of pollution impacts on ecosystems

We used the fluoride concentration in plants as a direct measure of environmental contamination along the Volkhov pollution gradient. Birch leaves (approximately 1 g dry weight per sample) were collected on 21 September 1988 at eight sites (five trees per site), on 18 September 1994 at 15 sites (five trees per site), and on 26 June 2025 at nine sites (two trees per site). Samples collected in 1988 were analysed using a colorimetric method at the Volkhov Sanitary and Epidemiological Station (Russia). Samples collected in 1994 and 2025 were analysed using a potentiometric method at the Finnish Forest Research Institute (Finland) and at Enviroservis (Slovakia), respectively. The latter laboratory used a fluoride ion-selective electrode (Orion Model 96-09) in an automatic SINTALYZER system (SINTEF ADR 7034), with results verified against a blank test and the certified reference material GBW07604 (poplar leaves).

We compared the patterns of pollution impacts on biota around Volkhov and Syasstroy, which differ in the composition of their aerial emissions, using soil pH and the needle longevity of Norway spruce. This first indicator is widely used to assess soil quality, while the second consistently declines from low- to high-stress sites in both natural and anthropogenic gradients (Kozlov et al., 2009, 2025). On 1 June 1989 and 26 June 2025, we counted the annual nodes bearing at least one live needle on two first-order branches located on opposite sides of the crown of ten haphazardly selected mature Norway spruce trees at each site where this species was present (24 sites in 1989 and 15 sites in 2025). The counts were averaged to obtain tree-specific values. Soil pH was measured on 26 June 2025 using a digital tester (PH328; Smart Sensor, Dongguan, China) at 10 points spaced 5 m apart at each of the 23 sites sampled at that time.

### Herbivore population densities

Following a previously validated approach (Kozlov et al., 2009, 2017a), we based our study on counts of Recognisable Taxonomic Units (RTUs). Herbivores and traces of their feeding (mines, leaf rolls and larval nests) attributable to specific RTUs (Table 1) were recorded at 27 study sites (15 around Volkhov and 12 around Syasstroy) between 20 May and 26 June, and again between 9 August and 25 September. Censuses were conducted annually from 1988 to 2005, and subsequently in 2017 and 2025.

At each site, the counts of each RTU were made on ten haphazardly selected birch trees 40–400 cm tall. Where trees of this size were absent, low-canopy branches of larger trees were examined. Depending on the birch density, the surveyed area ranged from 200 to 2000 m^2^ per site. Three observers were involved in data collection between 1988 and 2025, with approximately 85% of the data collected by the authors.

The leaf area (*S*, m^2^) of each surveyed tree or branch was estimated from its basal diameter (d, mm; corrected for dead shoots or stems, if present) using the equation: *S* = 1.234 × 10^−3^ × d^2^ (Zvereva & Kozlov, 2006). Data from all surveyed trees or branches at each site were summed (Data S1), and the densities of each RTU were expressed per 1 m^2^ of birch leaf area.

### Birch leaf quality

Relative growth rate (RGR) of autumnal moth *Epirrita autumnata* larvae has been used as an integrative measure of birch foliage quality because RGR reflects the effects of leaf nutritional and defensive characteristics on herbivore performance (Ossipov et al., 2001; Suomela et al., 1995). On 14 June 2017, 40 larvae reared from eggs under laboratory conditions were individually weighed to the nearest 0.1 mg and placed in 50 mL plastic vials, and each larva was provided with leaves from a single birch tree. The leaves were collected from five trees at each of the two most polluted and the two least polluted sites around each of the two polluters. After 24 h, the larvae were reweighed (Data S2) and the RGR was calculated as the increase in live mass divided by initial mass.

### Leaf area loss

Insect herbivory on downy birch was assessed in early summer and mid-autumn of 2011, 2017 and 2025 at the same eight sites used for leaf quality measurements. At each site, one branch bearing 50–200 leaves was collected from each of five mature downy birch trees, located at least 10 m apart. To minimise selection bias, branches were chosen while the observer was at a distance of 5–10 m away from the tree.

In the laboratory, each leaf was assigned to one of seven visually defined damage classes (0%; 0.01–1%; 1–5%; 5–25%; 25–50%; 50–75%; 75–100%). Herbivory was estimated by multiplying the number of leaves in each class (Data S3) by the midpoint of that class (e.g. 0.5% for 0.01–1%, 3% for 1–5%), summing these products across classes and dividing by the total number of leaves (Alliende, 1989).

### Data analysis

Spatial variation in pollution indices was analysed by correlating site-specific means with log-transformed distances from polluters (SAS CORR procedure; SAS Institute, 2009). Differences in spatial patterns between years and polluters were tested using ANCOVA (SAS GLIMMIX procedure, Type III sums of squares).

Variation in herbivore densities between censuses was analysed using the Kruskal–Wallis test (SAS NPAR1WAY procedure). Shannon diversity indices were calculated based on RTU counts. These metrics were related to distance from a focal polluter and sampling year using Spearman rank correlations (SAS CORR procedure).

Predictions were generally tested using meta-analysis of spatial and temporal patterns in herbivore density and diversity conducted in MetaWin 3 (Rosenberg, 2024). Correlation coefficients were *z*-transformed and weighted by their sample sizes to calculate effect sizes (ES). A general model was used to estimate overall mean ESs and 95% confidence intervals (CI95). Additional models tested differences among polluters, periods of high- versus low-emission levels, sites with elevated versus background pollution levels, herbivore feeding guilds, and abundance classes by calculating between-group heterogeneity (*Q*_B_). Effects were considered significant when CI95 ranges excluded zero.

Variation in log-transformed RGR was analysed using two-way ANOVA (SAS GLM procedure), with polluter and pollution level as factors. Leaf area loss was analysed using mixed-effects ANOVA (SAS GLIMMIX procedure), with pollution gradient, pollution level, year, and their interactions as fixed effects, and sites nested within gradients as a random intercept term.

## Results

### Pollution levels and impacts on ecosystems

Fluoride concentrations in birch leaves increased significantly with increasing proximity to Volkhov during the entire study period (1988: Fig. 3a; 1994: *r* = −0.88, *n* = 15 sites, *P* = 0.002; 2025: Fig. 3b). However, concentrations declined markedly over time (*F*_2, 26_ = 25.0, *P* < 0.0001), with peak values decreasing from 200 µg g^–1^ in 1988 to 125 µg g^–1^ in 1994 and to 32 µg g^–1^ in 2025, while background levels remained unchanged. Consequently, the Volkhov pollution gradient is now significantly shallower than it was at the beginning of our study (interaction with distance: *F*_2, 26_ = 17.4, *P* < 0.0001).

**Fig. 3:**
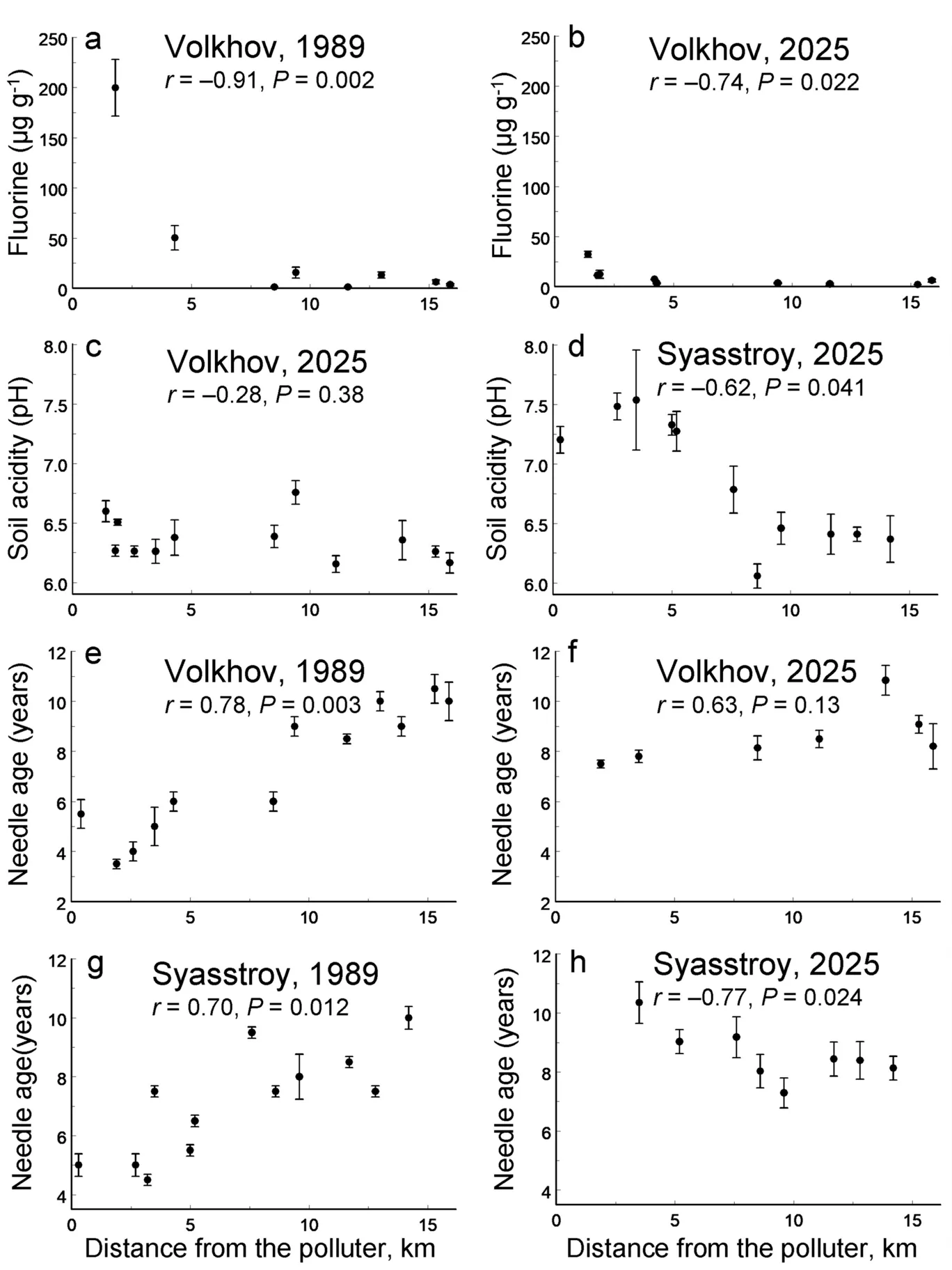
Environmental quality in the vicinity of Volkhov (**a**–**c**, **e**, **f**) and Syasstroy (**d**, **g**, **h**): **a**, **b** fluoride concentrations in leaves of *Betula pubescens*; **c**, **d** soil pH; **e**–**h** needle longevity of *Picea abies*. Values are means ± SE. Sample sizes: **a** 5 trees; **b** 2 trees; **c**, **d** 10 measurements; **e**–**h** 10 trees. All correlation coefficients were calculated using log-transformed distances

In 2025, soil pH did not vary along the Volkhov gradient (Fig. 3c), whereas it increased significantly with increasing proximity to Syasstroy (Fig. 3d). Nevertheless, the difference between the slopes of these two gradients did not reach statistical significance (interaction term:* F*_1, 19_ = 2.92, *P* = 0.10).

In 1989, Norway spruce needle longevity declined similarly with increasing proximity to both polluters (Fig. 3e, g:* F*_1, 20_ = 0.88, *P* = 0.36). However, by 2025, the patterns diverged, with needle longevity recovering to background levels near Volkhov (Fig. 3f), but exceeding background values 3.5 km from Syasstroy (Fig. 3h), resulting in a reversed (relative to 1989) spatial pattern (*F*_1, 11_ = 9.99, *P* = 0.009).

### Herbivore abundance and differences between early and late summer censuses

**Fig. 4:**
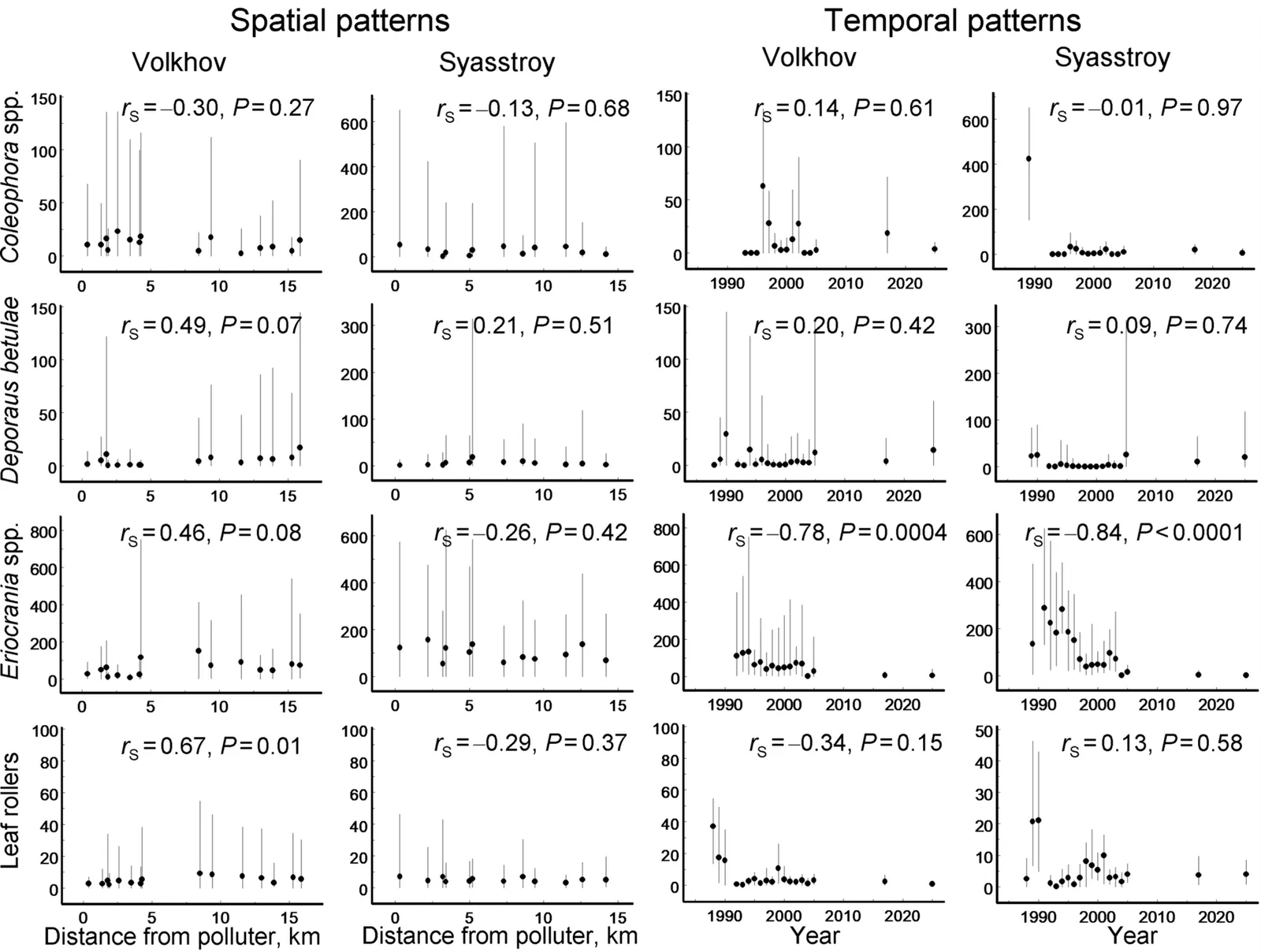
Density patterns of the four most abundant Recognisable Taxonomic Units. Dots indicate mean values; lines show minimum and maximum values

Across the 27 study sites and 9674 sampling units (downy birch trees or branches), we recorded 70,473 observation units of birch-feeding herbivores (insect larvae or diagnosable traces of their feeding, such as mines, rolls, or leaf shelters), attributable to over 60 species grouped into 24 RTUs (Table 1 and Data S1). The most common RTUs were *Coleophora* spp., *Deporaus betulae*, *Eriocrania* spp., *Incurvaria pectinea*, leaf rollers, leaf-mining sawflies, *Parornix* spp., and *Stigmella* spp. (Fig. 4; 1428–42,281 records).

Four extremely rare RTUs (Table 1; < 10 records each) were excluded from meta-analyses, although they were retained in RTU-specific analyses and diversity calculations. The densities of 16 RTUs differed significantly between the early and late summer (Table S3). Accordingly, further analyses were based on early summer data for four RTUs, late summer data for 14 RTUs, and averages of both censuses for six RTUs.

### Spatial patterns of herbivore density and diversity: high-emission versus low-emission years

**Fig. 5:**
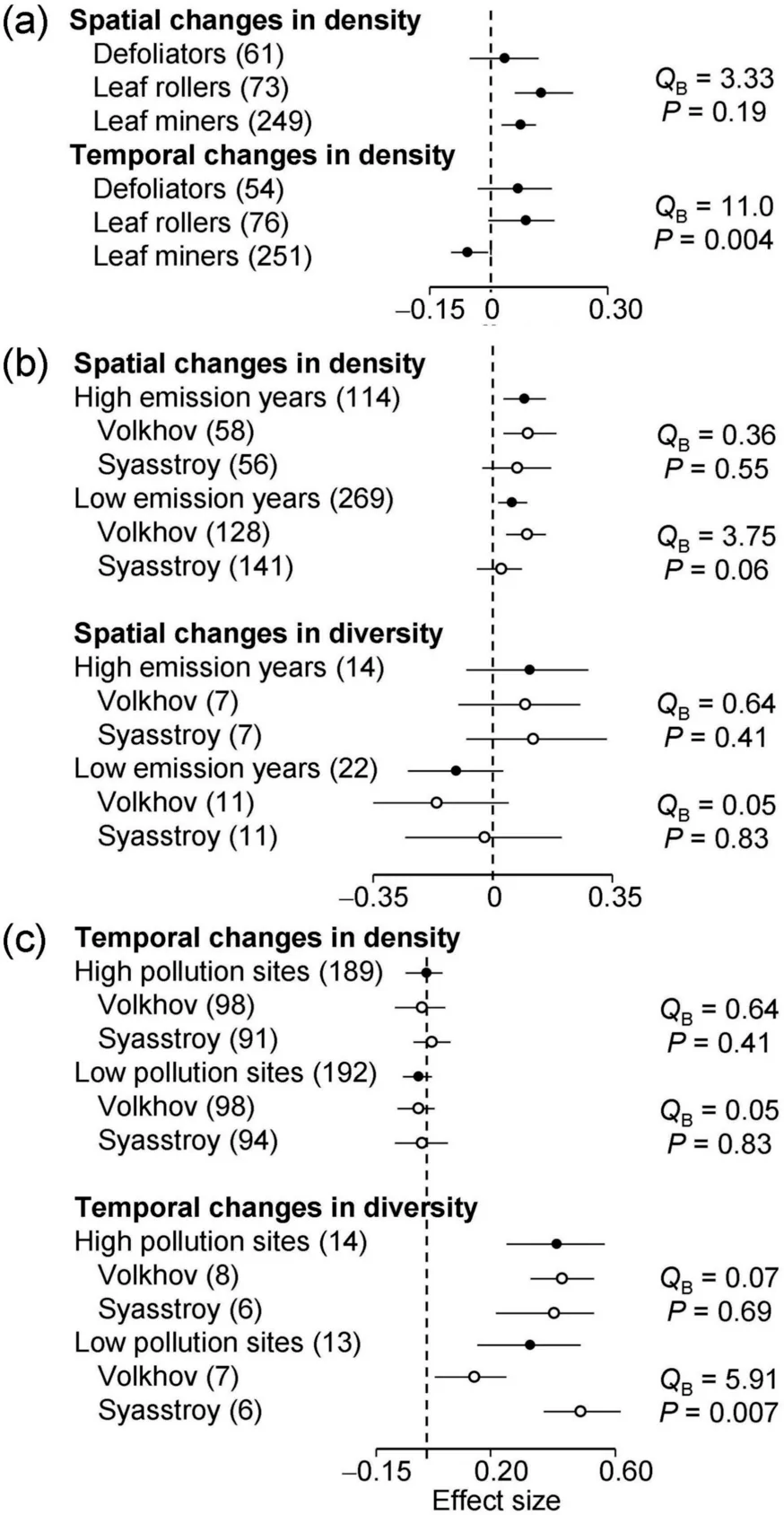
Spatial (from unpolluted to most polluted sites) and temporal (from 1988 to 2025) changes in density and diversity of birch-feeding insects: **a** differences among herbivore feeding guilds; **b** differences between periods of high (1988–1996) and low (1997–2025) aerial emissions to ambient air; **c** differences between heavily to moderately polluted sites (0.3–5.2 km from the polluters) and slightly polluted to relatively unpolluted sites (7.6–15.9 km from the polluters). Positive effect sizes indicate increases in trait values near polluters (spatial trends) or over time (temporal trends). Open circles: polluter-specific means; filled circles: overall effects across both polluters. Horizontal lines show 95% confidence intervals; sample sizes (numbers of effect sizes) are given in parentheses. Effects are considered significant when the confidence interval does not include zero. Significant (*P* < 0.05) *Q*_B_ values indicate differences among feeding guilds (**a**) or between polluters (**b**, **c**)

Across the entire study period, only 8.1% of the 383 RTU-by-year correlations between herbivore density and distance from the polluter were statistically significant at *P* = 0.05. In agreement with prediction 1 (Table S4), even the directions of these significant correlations sometimes differed among years within the same RTUs (Tables S5, S6). Nevertheless, our meta-analysis revealed significant overall variation in spatial patterns among RTUs (*Q*_B_ = 44.1, df = 19, *P* = 0.005), with six RTUs increasing with increasing proximity to the polluter, whereas the remaining 14 RTUs showed no association with pollution (examples shown on Fig. 4). This variation was not explained either by herbivore abundance (*Q*_B_ = 1.16, df = 1, *P* = 0.28) or by feeding guild (Fig. 5a). The Shannon diversity index decreased significantly with increasing proximity to Volkhov in only 1 year and showed no relationship with distance in any of the 20 study years around Syasstroy (Table S7).

During the period of relatively high emissions (1988–1996), the overall abundance of herbivorous insects increased with increasing proximity to the polluters (Fig. 5b), thus supporting prediction 2, although, contrary to prediction 3, the increase was statistically significant only near Volkhov. Diversity did not change around either Volkhov or Syasstroy (Fig. 5b).

During the period of relatively low emissions (1997–2025), the overall abundance of herbivorous insects increased with increasing proximity to the polluters at the same rate as during the high-emission period (Fig. 5b; difference between periods: *Q*_B_ = 0.82, df = 1, *P* = 0.36), whereas the direction of the diversity change shifted to negative (Fig. 5b; difference between periods: *Q*_B_ = 3.76, df = 1, *P* = 0.04). Thus, prediction 4 was not supported (Table S4).

### Temporal patterns of herbivore density and diversity: high versus low pollution sites

Across the entire study period, only 12.1% of the 380 RTU-by-site correlations between herbivore density and study year were statistically significant at *P* = 0.05. In contrast to the spatial patterns, the directions of these significant correlations were always consistent within individual RTUs (Tables S8, S9). Accordingly, our meta-analysis revealed significant overall variation in the temporal patterns among RTUs (*Q*_B_ = 429.3, df = 19, *P* < 0.001), which was partly explained by the herbivore feeding guild (Fig. 5a). Notably, leafminer densities decreased over time, whereas defoliator densities remained unchanged and leafroller densities tended to increase (Figs. 4, 5a), supporting prediction 5 regarding the shift in herbivore community composition (Table S4). The Shannon diversity index increased significantly with study year at two sites around Volkhov and at five sites around Syasstroy (Table S10).

Contrary to our expectations (predictions 6 and 7), we observed no differences in the temporal changes in communities of birch-feeding insects in moderately to heavily polluted sites, located 0.3–5.2 km from the polluters, when compared with the changes observed in relatively unpolluted sites located 7.6–15.9 km from the polluters. In both groups of sites, the herbivore abundance did not change over time (Fig. 5c), whereas herbivore diversity increased substantially (Fig. 5c). Within the slightly polluted and unpolluted sites, the temporal changes in diversity around Syasstroy were greater than around Volkhov (Fig. 5c).

### Leaf quality for herbivores

**Fig. 6:**
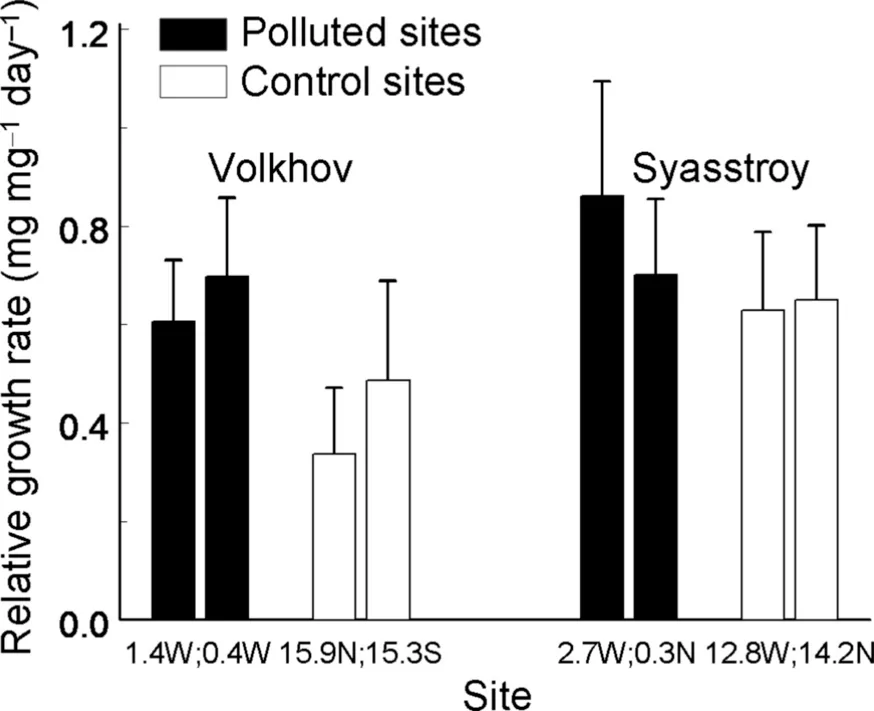
Relative growth rate of third-instar larvae of the autumnal moth, *Epirrita autumnata* (means + SE; *n* = 5 larvae per site), near Volkhov and Syasstroy. See main text for statistical results

The density of the autumnal moth, *E. autumnata*, did not change with an increase in pollution level (ES = 0.07, *n* = 10, CI95 = −0.08 to 0.21). Nevertheless, its larvae grew 45% faster on birch leaves collected in 2017 from sites closest to the polluters when compared to their growth on leaves from the most distant sites (Data S2; Fig. 6; *F*_1, 4_ = 42.0, *P* = 0.0029). This effect was stronger near Volkhov than near Syasstroy (78% and 26%, respectively; pollution level by polluter interaction: *F*_1, 4_ = 9.06, *P* = 0.04).

### Foliar damage

**Fig. 7:**
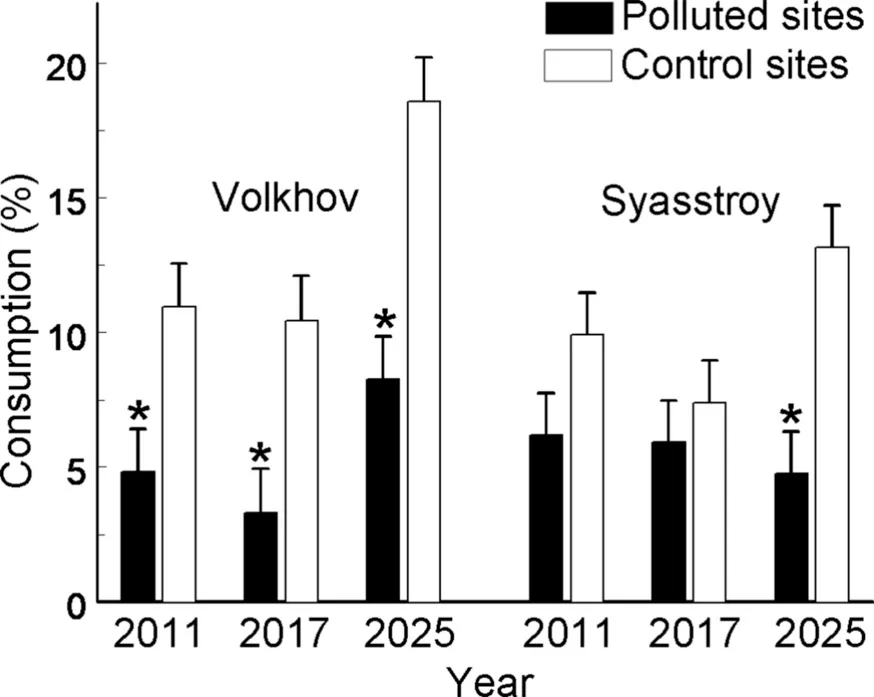
Damage of birch foliage around Volkhov and Syasstroy (estimated marginal means + SE). An asterisk indicates a significant difference (*P* < 0.01) between the most and least polluted sites (mixed-model ANOVA, *t*-test). Full statistical results are provided in Table S11

Across the study years, the insect-inflicted birch foliage losses near both Volkhov and Syasstroy (Data S3) were approximately half of the losses recorded at the most distant sites (Fig. 7). This reduction was statistically significant and did not differ between the two polluters (Table S11).

## Discussion

### Temporal changes in environmental contamination

The main industrial enterprises of Volkhov (aluminium smelter) and Syasstroy (pulp and paper mill) were commissioned in 1932 and 1928, respectively. By the mid-1950s, the peak concentrations of sulphur dioxide in Volkhov had reached 30 mg m^–3^ (Kijamov, 1959), or approximately 750 times higher than the currently recommended health-protective level (40 µg m^–3^ over 24 h; World Health Organization, 2021). Historical pollution data for Syasstroy are scarce, but the local ecosystems were classified as being in a “critical” or even “catastrophic” state in the 1990 s (Melnikova, 2001). Thus, the data collected at the beginning of our study reflect the legacy of decades of substantial pollution impact, whose effects were expected to remain evident (prediction 2).

Both the Volkhov smelter and the Syasstroy mill were designated in the early 1990 s as regional pollution “hot spots” by the Baltic Marine Environment Protection Commission (Ministry of the Environment of Finland, 1991) and were included in the environmental priority action programme. This international attention facilitated investments aimed at reducing emissions (HELCOM, 2013), but the consequent changes in terrestrial ecosystems have not been monitored. Our study helps to reduce this knowledge gap.

The reported fluoride emissions to ambient air in Volkhov decreased 16-fold between 1989 and 2012, whereas the peak fluoride concentrations in birch leaves declined only sevenfold between 1988 and 2025. This mismatch may reflect a legacy effect (Arnesen et al., 1995; Cronin et al., 2000). However, around 2010, the fluoride concentrations in soil near the smelter exceeded background levels by only threefold (Shavrina & Gavrilenko, 2012), whereas birch leaves in 2025 showed a tenfold difference (Fig. 3b). This discrepancy suggests that fluoride in birch leaves may originate primarily from surface deposition rather than root uptake; therefore, the fluoride content may reflect current emissions rather than legacy contamination.

No data are available regarding the fluoride content of industrial dust currently emitted by the fertiliser-producing enterprise in Volkhov. Assuming that it is similar to the 3.7% reported for a comparable facility in Akmalyk, Uzbekistan (Litvinovich et al., 2021), we estimate that the current annual fluoride emission in Volkhov is approximately 90 t. This estimate is more consistent with our data on temporal changes in fluoride content in birch leaves than it is with the available governmental data (Table S1). Nevertheless, environmental contamination around both Volkhov and Syasstroy had declined substantially during the study period, so the weakening or even complete disappearance of the adverse effects on biota was justifiably expected (prediction 4).

### Temporal changes in the vitality of Norway spruce

Short needle-retention time has long been recognised as a visible sign of the damaging effects of air pollution on coniferous trees (Manning & Feder, 1980). This response, driven by accelerated needle ageing in industrial areas (Wulff et al., 1996), occurs under exposure to several pollutants, including sulphur dioxide and fluoride (Kozlov et al., 2009). Therefore, needle longevity is currently recognised as a reliable bioindicator for comparing the environmental impacts of different polluters.

At the beginning of our study (1989), the maximum age of Norway spruce needles in the vicinity of both Volkhov and Syasstroy was reduced to 3–6 years, i.e. roughly half of its background value (Fig. 3c, e). Combined with the comparable levels of aerial emissions (Table S1), this decline suggested that the magnitudes of pollution impacts at our two towns were only slightly weaker than the magnitude reported at the large-scale smelter at Monchegorsk (Kozlov et al., 2009), and showed similar spatial patterns of pollutant distribution. The decline in aerial emissions over time was followed by recovery of needle longevity around Volkhov to 7–8 years by 2025, whereas the needle longevity near Syasstroy reached 10–11 years and even exceeded the regional background values (Fig. 3c–f).

The divergence in spruce vitality patterns between Volkhov and Syasstroy may be linked to differences in soil development following emission decline. Near Volkhov, soil that was significantly more alkaline than background levels in 2006 (Kozlov et al., 2009) no longer differed from the control by 2025 (Fig. 3g), whereas the soil near Syasstroy was more alkaline in 2025 (Fig. 3h). Thus, both soil pH and spruce vitality suggested that the nature of plant–insect interactions was likely to have changed from 1988 to 2025, although they did so in distinct ways around the two industrial towns (prediction 3). However, this expectation was not met.

### Is air pollution the leading driver of the observed patterns?

One of the most striking outcomes of our long-term, multisite study is the low magnitude of the density increase of birch-feeding insects with increasing proximity to either of the two polluters during the high-emission period. In particular, only 8.1% of the correlations between herbivore density and distance from the polluter (across all RTU-by-year combinations) were statistically significant, and this proportion only marginally exceeded the 5% expected by chance. Consistently, the overall ES = 0.10 detected by our study is considered small (Cohen, 1988), whereas previous meta-analyses reported modest to large effects of pollution on the abundances of herbivorous insects (Vidal et al., 2026; Zvereva & Kozlov, 2010).

Because the strength of pollution effects on biota generally increases with pollution intensity (Kozlov & Zvereva, 2011), the small effect on insect herbivores detected in our study could reflect proportionally weaker environmental impacts of the emissions at Syasstroy and Volkhov relative to previously studied polluters. However, the median annual SO_2_ emission at Syasstroy in 1988–1995 was approximately 5500 t (Table S1), so it matched the median emissions from the 74 industrial enterprises included in the meta-analysis of insect responses to pollution (Zvereva & Kozlov, 2010). Similarly, fluoride emissions at Volkhov in 1989–1990 (375–378 t; Table S1) exceeded the median emission in the same dataset (265 t). Thus, despite NO_x_ emissions from both polluters at the beginning of our study being slightly smaller than the median value in the cited dataset (1370 t), the pollution levels near Syasstroy and Volkhov could still have been expected to induce much greater effects (approaching those reported by Zvereva & Kozlov, 2010) than those actually observed.

However, the latter meta-analysis demonstrated that the mean ESs, based on randomly chosen years from multiyear datasets on herbivore population densities, did not differ from zero, whereas publications reporting density data from single years have shown significantly positive ESs. Similarly, pseudoreplicated studies contrasting one polluted and one control site produced larger effects on herbivore population density than properly replicated studies (Zvereva & Kozlov, 2010). Taken together, these results suggest that previous meta-analyses incorporating single-year and pseudoreplicated studies have overestimated the overall impact of pollution on herbivorous insects; therefore, our current findings are more indicative of the true effect size.

Another striking finding is the persistence of increased herbivore density near polluters after substantial emission declines. This persistence questions whether air pollution is the primary driver of the observed patterns, and it suggests that the increase in herbivore density may instead be attributable to a factor, such as urbanisation, that remained relatively stable from 1988 to 2025. However, this explanation is only weakly supported because urbanisation has no significant overall effect on insect herbivore abundance, with positive effects observed only in sap-feeders (Korányi et al., 2022), and because the study towns were too small to produce significant urbanisation effects on herbivores and herbivory (Kozlov et al., 2017b).

Both the spatial and temporal patterns detected in our study are difficult to reconcile with the approximately 40% improvement observed in birch leaf quality for one of our study species, *E. autumnata*. Such an improvement might be expected to alter its population dynamics substantially (Kozlov, 2022; Zvereva & Kozlov, 2010) but did not, in fact, affect it. They also do not explain the reduction in leaf damage near both polluters to approximately half the level observed at unpolluted sites or the shift from positive effects of pollution on herbivore diversity during high-emission years to negative effects during low-emission years, possibly because other herbivore species respond to pollution-induced changes in birch foliage in a different way than *E. autumnata* did.

Previous studies have reported a similar decoupling between plant quality, herbivore abundance, and plant damage due to interactions between bottom-up and top-down processes, as well as taxon-specific responses and community-level averaging (Campbell & Vallano, 2018; Stiling et al., 1999; Zvereva & Kozlov, 2010). In particular, improved host plant quality does not necessarily translate into higher herbivore densities or increased herbivory; instead, it may reduce insect consumption rates (DeLucia et al., 2012), thereby lessening plant damage. Consequently, environmental gradients may leave only weak or inconsistent signatures at the community level (Hillebrand et al., 2008; Kozlov et al., 2009). Although these types of patterns can often be explained post hoc, they remain difficult to predict a priori.

From an applied perspective, the observed inconsistencies highlight a key limitation of bioindication, namely that conclusions regarding ecosystem health depend strongly on the indicators used (Kozlov et al., 2025). In our study, pollution gradients were clearly reflected in fluoride accumulation and, historically, in reduced spruce vigour, whereas herbivore density and diversity suggested weak overall impacts due to the pronounced spatial and temporal variations of their associations with pollution level. Ignoring these variations can easily lead to biased conclusions. For example, if *Eriocrania* spp. had been used as the sole representative of insect herbivores, the 1994 data would suggest decreased herbivory near Syasstroy, while the 2017 data would suggest increased herbivory, and data from other years would suggest no effect (Table S10). Likewise, comparing the data on *Eriocrania* spp. from 1994 and 2005 would have led to the conclusion that emission reductions eliminated negative effects on herbivory.

This context dependence of bioindicators is rarely documented, although different indicators may yield contrasting assessments of environmental quality (Kozlov et al., 2025; McMahon et al., 2012; Niemi & McDonald, 2004). Given that most studies of pollution effects on insects are based on single-year data (Kozlov et al., 2009; Zvereva & Kozlov, 2010), our results call into question the reliability of datasets used to identify insect bioindicators. At a minimum, they challenge the generality of the view that insects are universally sensitive to even slight environmental changes (Chowdhury et al., 2023).

### Did birch-feeding insects decline from 1988 to 2025?

In the context of growing concern about global biodiversity loss, insects have received considerable attention, as individual studies and systematic reviews have reported substantial overall declines in terrestrial insect populations over recent decades, while also acknowledging that trends differ widely among taxa, habitats and regions (van Klink et al., 2020; Vidal et al., 2025). However, the fragmented nature of the evidence limits our understanding of both this variation and the coexistence of forecasts predicting global insect decline alongside increased insect herbivory under climate change (Hamann et al., 2021; Jamieson et al., 2012).

To help address this gap in knowledge, we analysed temporal changes in communities of birch-feeding insects. Initially, we focused on unpolluted and slightly polluted sites (prediction 6), as we expected these to reflect broader temporal trends rather than local effects of environmental disturbance. However, because we found no differences in temporal patterns between heavily polluted and unpolluted sites, we searched for general patterns across the entire dataset.

First and most importantly, the detected changes in density varied among insect groups (RTUs), ranging from increases to decreases over time and yielding no net effect overall. Thus, although we found no evidence of decline among birch-feeding insects, we also did not detect the increase in their abundance predicted from latitudinal patterns (Bracalini et al., 2024; Kozlov et al., 2013) and confirmed in German forests (Staab et al., 2023). This discrepancy suggests that temporal trends in insect abundance may differ not only among trophic levels but also among regions.

At the same time, our findings were consistent with data on birch herbivores collected around a copper–nickel smelter at Monchegorsk, approximately 900 km north of Volkhov and Syasstroy, where annual censuses were conducted from 1991 to 2016 (Kozlov et al., 2017a). In agreement with that study, we found that the absence of an overall trend in herbivore abundance resulted from a combination of increasing densities in leafminers, stable densities in externally feeding defoliators, and declining densities in leaf rollers. This consistency suggests that conclusions regarding temporal shifts in herbivorous insect populations may depend strongly on the feeding guild examined.

Finally, we detected an increase in the diversity of birch-feeding insects during the observation period. The most plausible explanation for this trend is the northward arrival of insect species from more southern regions due to an increase in the mean ambient temperature in the St. Petersburg region from 5.7 to 6.7 °C between 1985 and 2024 (Nikolaeva, 2025), followed by a gradual increase in the contribution of these species to local communities (Bracalini et al., 2024; Kozlov & Zvereva, 2017).

### Limitations of the study

Despite the unusually long temporal span and multi-site structure of our dataset, several limitations should be considered when interpreting the results. First, although our sampling covers 1988–2025, the temporal resolution is uneven across years and RTUs, and not all taxa were consistently recorded throughout the entire study period. This introduces potential biases in estimating long-term trends and may reduce the power to detect subtle directional changes in population densities.

Second, while the study is based on two historically well-characterised industrial pollution gradients, the available emission and deposition data are incomplete, particularly for earlier decades and for certain pollutants (e.g. industrial dust composition at Syasstroy and contemporary fluoride emissions in Volkhov). Consequently, part of the inferred exposure history relies on indirect estimates, which may introduce uncertainty into comparisons between polluters and across time.

Third, although we demonstrate substantial declines in emissions and improvements in several ecosystem indicators, environmental conditions were not experimentally controlled. Other co-occurring drivers, such as climate warming and regional atmospheric deposition, likely changed during the same period and may have influenced both plant and insect responses. While we considered some of these factors qualitatively, their relative contributions cannot be fully disentangled in an observational framework.

Finally, our insect data are largely based on Recognisable Taxonomic Units, which only partially correspond to species-level identifications. Although RTUs are widely used in long-term ecological monitoring and provide a consistent operational taxonomic framework, they may mask species-specific responses, particularly if species composing an RTU differ in their sensitivity to pollution or climatic change. This could contribute to the observed variability in responses among RTUs and weaken detectable trends at the community level.

## Conclusions

Our study supported only a handful of the predictions derived from previous studies of the ecology of polluted habitats. Moreover, several findings of the present study can be classified as “ecological surprises,” defined as unexpected observations about the natural environment (Lindenmayer et al., 2010). These include the weakness of spatial changes in the abundance and diversity of herbivorous insects across pollution gradients and the absence of temporal changes in either polluted or unpolluted sites despite emission decline and climate warming. The lack of adverse effects on insects near two industrial towns previously identified as pollution hotspots calls into question the role of industrial air pollution as a driver of insect decline, at least for herbivores. Similarly, the absence of long-term (1988–2025) changes in the overall abundance of birch-feeding insects, accompanied by their increased diversity, contrasts with forecasts of both insect decline and increased herbivory under global change. Documenting these types of ecological surprises is critically important because their analysis can prompt re-evaluations of established views and improve assessment of how well a priori predictions can capture real ecological patterns and processes.

## Supplementary Information

Below is the link to the electronic supplementary material.

ESM 1 (PDF 1.51 MB)

## Data Availability

All data are in the manuscript or supporting material.
