# Supplementary material for: Weak and inconsistent associations between birch-feeding insects and pollution levels around two industrial towns during substantial emission decline (1988–2025)
Source: Environ Monit Assess. 2026 Oct 7;198(11):1157. doi: 10.1007/s10661-026-15971-1 (PMC13645969; doi:10.1007/s10661-026-15971-1)
Supplement: Supplementary file 1 [file 10661_2026_15971_MOESM1_ESM.pdf]

Weak and inconsistent associations between birch-feeding insects and pollution levels around two industrial towns during substantial emission decline (1988–2025)

Mikhail V. Kozlov*, Vitali Zverev
Department of Biology, University of Turku, FI-20014 Turku, Finland
* Corresponding author. E-mail address: mikoz@utu.fi (M.V. Kozlov).

Supplementary materials

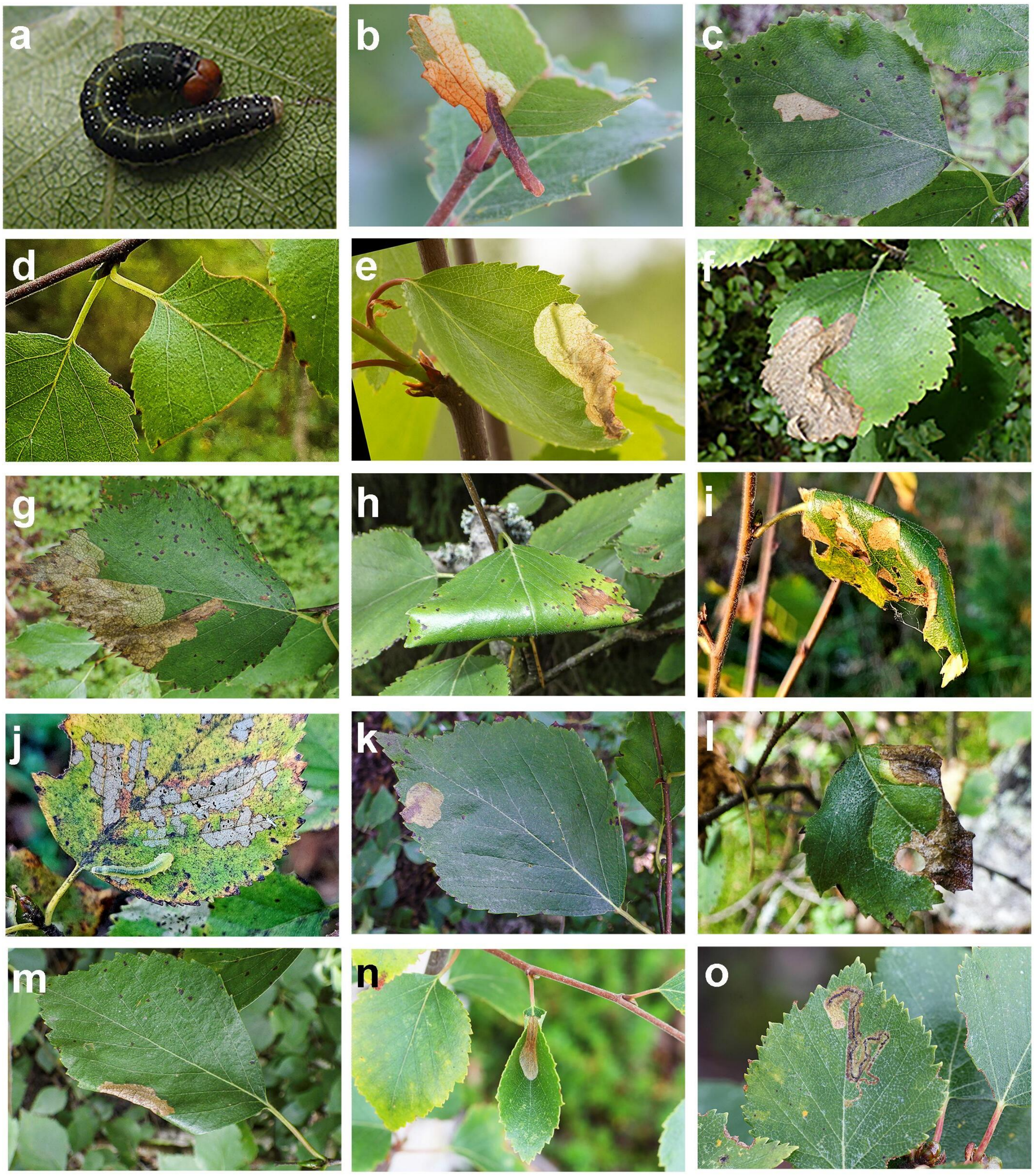

Figure S1

Selected Recognizable Taxonomic Units (RTUs): a, larva of *Achlya flavicornis* (L.); b, larval case of *Coleophora milvipennis* Z. attached to a leaf mine; c, mine of *Coleophora* sp.; d, leaf damaged by *Deporaus betulae* (L.); e, mine containing a living *Eriocrania* sp. larva; f, mine abandoned by an *Eriocrania* sp. larva; g, mine of *Eriocrania sparrmannella* (Bosc); h, i, shelters of leaf rolling moth larvae; j, larva of a leaf-chewing sawfly; k, mine of *Fenusella nana* (Klug); l, mine of *Phylloporia bistrigella* (Hw.); m, mine of *Parornix* sp.; n, mine of *Phyllonorycter corylifoliella* (Hbn.); o, mine of *Stigmella lapponica* (Wck.). For the complete list of RTUs, see Table 1. Photo: V. Zverev and M. V. Kozlov.

Table S1
Aerial emissions (metric tons) from industrial enterprises in Volkhov and Syasstroy.

Year	Volkhov[1]				Syasstroy		
	Dust	SO_2	NO_x	F	Dust	SO_2	NO_x
1988	6200	3600	1200		4132	6004	814
1989	4900	3000	1100	378	4108	5548	815
1990	4100	2700	800	375	4000	5500	800
1991	3669	2900	900	238			
1992	3000	3400	800	253	2500	5400	600
1993	2300	2600	600	133	1900	5900	600
1994	1700	1400	400	22	500	3600	300
1995	1400	2900	300	7	400	6200	400
1996	300	1300	200	18	400	3800	300
1997	200	1300	100	22	100	2800	200
1998	200	1500	300	16	20	1400	100
1999	200	1600	300	16	10	680	100
2000	700	1300	300	19	11	100	146
2001	800	600	200	16	30	90	450
2002	2400	1500	400	16	30	200	500
2003	2400	1500	400	15	20	10	500
2004	2600	1100	300	26	20	80	600
2005	2000	1000	300	24	20	60	600
2006	1800	500	300				
2007	4400	600	300				
2008	4500	500	300	22		56	554
2009	2130	600	2100	19		51	567
2010	900	1000	900	27		57	629
2011	800	800	300	26		50	429
2012	1090	1010	460	24		49	428
2013	898	742	617		18	48	510
2014	1300	560	750		7	46	515
2015	1300	800	1200				
2016	1100	1000	1100				
2017	2100	500	1400				
2018	622	155	323				
2019	610	162	150				
2020	2500/462	700/181	3200/283				
2021	2300/529	800/207	3300/208				
2022	235	320	331				
2023	215	352	224				
2024	287	493	224				

[1] Data for 2020–2021 differ between sources, with lower values reported by the main polluter.

Table S2
Basic characteristics of study sites located around Volkhov and Syasstroy.

Town	Site code [a]	Latitude, ºN	Longitude, ºE	Habitat
Volkhov	15.9 N	60.010833	32.154167	Forest
	13.9 N	59.993240	32.175800	Forest
	11.6 N	59.973980	32.195510	Forest
	8.5 N	59.947880	32.226950	Forest
	4.2 W	59.926270	32.291320	Forest
	3.5 W	59.927990	32.307780	Urban
	2.6 W	59.927550	32.330830	Urban
	1.8 W[b]	59.921930	32.339460	Rural
	1.4 W	59.920050	32.346200	Rural
	0.4 W[c]	59.909040	32.350810	Urban
	1.9 S	59.891900	32.363520	Urban
	4.3 S	59.871450	32.360140	Rural
	9.4 S	59.825290	32.367230	Rural
	13.0 S[d]	59.792020	32.362250	Forest
	15.3 S	59.772160	32.360650	Forest
Syasstroy	12.8 W	60.082990	32.360340	Forest
	11.7 W	60.088950	32.376920	Forest
	9.6 W	60.097530	32.410110	Forest
	7.6 W	60.105450	32.443960	Forest
	5.2 W	60.114200	32.476990	Forest
	3.5 W	60.124130	32.507150	Forest
	2.7 W	60.140480	32.524750	Rural
	0.3 N	60.135590	32.566030	Urban
	3.2 N[e]	60.160080	32.584880	Forest
	5.0 N[f]	60.173760	32.603240	Forest
	8.6 N	60.196490	32.653530	Forest
	14.2 N	60.228890	32.732210	Forest

[a] Distance (km) and direction from the polluter.
[b] The site was destroyed between 2017 and 2025 due to construction.
[c] Birch trees were removed between 2005 and 2017, and the site was converted into an urban recreational area between 2017 and 2025.
[d] The site became inaccessible in 2025 due to colonisation of the area between the roadside and forest edge by *Heracleum sosnowskyi*.
[e] Birch trees were removed between 2004 and 2005 as part of roadside maintenance.
[f] Birch trees were removed between 2005 and 2017 as part of roadside maintenance.

Table S3
Differences in RTU[a] density between early and late summer censuses (Kruskal–Wallis test).

Herbivore species/group (RTU[a])	Mean density (units m^{-2} of leaf area)[b]		Differences between censuses	
	Census 1	Census 2	χ^2_1	*P*
Achlya flavicornis	0.00000	**0.00494**	1.71	0.19
Agromyza alnibetulae	0.019	**0.337**	51.8	<0.0001
Apoderus coryli	**0.00186**	0.00000	1.17	0.28
Bucculatrix demaryella	0.0000	**0.0140**	4.28	0.04
Coleophora spp.	**18.65**	5.75	180.9	<0.0001
Colocasia coryli	0.0017	**0.0240**	3.65	0.05
Deporaus betulae	**5.78**	**4.95**	0.14	0.71
Drepanidae	0.0024	**0.0150**	5.25	0.02
Ectoedemia minimella	0.004	**0.194**	22.0	<0.0001
Epirrita autumnata	**0.0895**	0.0243	13.4	0.0002
Eriocrania sparrmannella	0.0074	**0.0360**	12.6	0.0004
Eriocrania spp.	**79.19**	2.49	521.0	<0.0001
Geometridae	**0.0232**	**0.0968**	3.05	0.08
Incurvaria pectinea	**0.592**	**0.632**	2.99	0.08
Leaf rollers	1.76	**5.03**	93.1	<0.0001
Leaf-chewing sawflies	0.100	**0.158**	4.85	0.03
Leafmining sawflies	0.004	**3.052**	336.9	<0.0001
Notodonta dromedarius	**0.0052**	**0.0104**	0.03	0.86
Orchestes spp.	0.0065	**0.0544**	10.3	0.0013
Parornix spp.	0.14	**4.28**	329.7	<0.0001
Phyllonorycter corylifoliella	0.0000	**0.0160**	2.67	0.10
Phylloporia bistrigella	0.003	**0.125**	40.2	<0.0001
Psychidae	**0.00849**	**0.00458**	0.04	0.84
Stigmella spp.	0.13	**2.45**	208.2	<0.0001

[a] RTU, Recognizable Taxonomic Unit.
[b] Boldface indicates the census used in subsequent analyses.

Table S4
Predictions and their support.

Prediction #	Response variable	Explanatory variable	Boundary conditions	Prediction	Support	Reference
1	Density	Distance	No	Diverse responses, negative to positive	Supported	Text #3.3
2	Density	Distance	High emission years	Increase	Supported	Fig. 5b
	Diversity	Distance	High emission years	Decrease	Not supported	Fig. 5b
3	Density	Distance	High emission years	Stronger increase near Syasstroy	Not supported	Fig. 5b
	Diversity	Distance	High emission years	Stronger decrease near Syasstroy	Not supported	Fig. 5b
4	Density	Distance	Low emission years	Increase weakens	Not supported	Fig. 5b
	Diversity	Distance	Low emission years	Decrease weakens	Not applicable[a]	Fig. 5b
5	Density	Year	No	Guild-specific changes	Supported	Fig. 5a
6	Density	Year	Low pollution sites	Decrease	Not supported	Fig. 5c
	Diversity	Year	Low pollution sites	Decrease	Not supported	Fig. 5c
7	Density	Year	No	Greater decrease in more polluted sites	Not applicable[b]	Fig. 5c
	Density	Year	No	Greater decrease in more polluted sites	Not applicable[b]	Fig. 5c

[a] Prediction was based on the assumption that prediction 2 for diversity was supported.
[b] Prediction was based on the assumption that prediction 6 was supported.

Table S5
Spearman rank correlation coefficients (r_S) between site-specific herbivore [a] densities and distance from Syasstroy in each study year [b].

Year	*n*	Agro	Cole	Colo	Depo	Drep	Ecto	Epir	Espa	Erio	Geom	Incu	Roll	Sawf	Mine	Noto	Orch	Paro	Pcor	Pbis	Stig
1988	8												–0.15					0.28			
1989	8	–0.41	–0.29		0.12					–0.02			–0.31					0.25			**0.84**
1990	8				–0.02						0.41		–0.57	0.17	0.07			0.07			–0.41
1991	8									0.12											
1992	12				–0.14	–0.16	–0.28		0.04	0.11			0.18					0.23			0.42
1993	11									–0.03			–0.10					0.15			0.34
1994	12	0.12			0.46			0.06		**–0.59**			–0.07		–0.04			0.19			0.07
1995	12				–0.24	0.31			0.12	–0.19	0.53		0.28	0.04	0.37			0.18			0.51
1996	12	–0.32	–0.30		0.06			–0.13		0.20			0.33		**0.61**			–0.17			0.05
1997	12		**0.75**		–0.25		–0.37		–0.22	0.35		0.22	0.38	0.48	–0.54			0.09			**0.57**
1998	11	–0.12	0.11		–0.44			–0.13		–0.20	–0.04	–0.18	0.38	0.48	–0.28	–0.05		–0.43			0.14
1999	12	0.11	0.18		–0.22		0.39	0.48		–0.03	–0.22		0.38	0.15	–0.09		0.44	–0.35			0.01
2000	12	0.09	0.15		0.48	0.31	–0.13			0.17	–0.31		–0.16	0.22	–0.04			0.01		0.23	–0.27
2001	12	0.09	**0.73**	0.31	0.01	–0.04	–0.05		–0.48	–0.24	0.39		–0.08	–0.06	–0.13		–0.31	0.10		0.52	0.39
2002	12	–0.22	–0.03		0.16		–0.04		–0.48	–0.25		–0.33	–0.01		0.24		0.04	0.23		0.39	–0.16
2003	12	0.22			0.02				0.39	0.43			–0.34		–0.15					0.30	
2004	12	–0.13			0.05				–0.48	–0.18			0.16	**–0.65**	–0.09			0.18		0.11	–0.13
2005	11	–0.10	–0.28		–0.10			0.10	**0.66**	0.27	0.20	–0.11	**–0.72**	0.32	–0.07	–0.30	–0.20	0.09	–0.20	0.39	–0.01
2017	11	0.04	0.10	0.11	–0.04	–0.50		0.10	0.11	**0.66**	–0.21	0.30	–0.04	0.09	**–0.72**		–0.30	–0.33			0.38
2025	10	–0.29	0.11		0.31				–0.41	–0.24	0.52	–0.01	0.02	0.17	–0.07			–0.18			–0.35

[a] Recognizable Taxonomic Units (RTUs) abbreviated as follows: Agro, *Agromyza alnibetulae;* Cole, *Coleophora* spp.; Colo, *Colocasia coryli;* Depo, *Deporaus betulae;* Drep, Drepanidae; Ecto, *Ectoedemia minimella;* Epir, *Epirrita autumnata;* Espa, *Eriocrania sparrmannella;* Erio, *Eriocrania* spp.; Geom, Geometridae; Incu, *Incurvaria pectinea;* Roll, Leaf rollers; Sawf, Leaf-chewing sawflies; Mine, Leafmining sawflies; Noto, *Notodonta dromedarius;* Orch, *Orchestes* spp.; Paro, *Parornix* spp.; Pcor, *Phyllonorycter corylifoliella;* Pbis, *Phylloporia bistrigella;* Stig, *Stigmella* spp.

[b] Empty cells indicate the absence of RTU during the study year. Boldface indicates statistically significant correlations ($P < 0.05$).

Table S6
Spearman rank correlation coefficients (r_S) between site-specific herbivore [a] densities and distance from Volkhov in each study year [b].

Year	*n*	Agro	Cole	Colo	Depo	Drep	Ecto	Epir	Espa	Erio	Geom	Incu	Roll	Sawf	Mine	Noto	Orch	Paro	Pcor	Pbis	Stig
1988	8	–0.68			–0.08								–0.36			–0.08		–0.52			–0.22
1989	13	0.22			0.32								**0.64**					0.39			–0.11
1990	14				0.49						–0.31		**0.56**	0.01	–0.22	–0.31		0.21			–0.06
1992	13				0.25		–0.01			0.31			–0.15					–0.21	–0.19		0.16
1993	14						0.39			0.06			0.41					–0.30			–0.29
1994	15	0.35			0.43	0.37		0.33		0.34			0.08		**0.57**			**0.68**			–0.28
1995	15		–0.03		0.27	0.31				0.40	–0.19	0.43	0.01	–0.37	0.46			–0.23			–0.42
1996	15		**–0.52**		**0.51**					**0.58**	–0.05		**0.69**		0.23			0.07			–0.24
1997	15		–0.29		0.30		–0.31	0.31	0.31	0.13	–0.43	–0.11	**0.58**		0.22			0.06	0.37		0.02
1998	15	–0.07	–0.23		0.17					0.14	0.16	–0.14	0.33		–0.09	0.31		–0.13			0.50
1999	15	0.05	–0.12	0.06	0.22					–0.30	–0.37		0.21		**0.62**		0.31	0.03			–0.25
2000	15		–0.16		0.41		0.08			0.28	0.43		–0.04		0.28			0.26		**0.52**	0.09
2001	15	–0.43	–0.32		0.03		–0.37			0.32	–0.37		–0.12	–0.43	0.11		0.01	0.00		**0.73**	–0.35
2002	15	0.17	–0.18		0.18		0.21	0.06		**0.56**	–0.02		–0.01		0.30			0.00		0.32	–0.20
2003	15	0.19			0.10				–0.43	**0.51**			–0.22	–0.25	0.14			0.20		0.38	–0.38
2004	15	0.06			–0.27					–0.29			–0.28	–0.19	0.34			–0.07		**0.51**	–0.40
2005	15	–0.31	0.48		0.35		0.43			**0.73**	0.19	0.28	–0.25	0.06	0.12	–0.19	0.31	**–0.55**		0.49	–0.03
2017	13		0.33	–0.23	**0.66**			–0.38	**–0.56**	0.50	**0.72**		**0.56**	0.13	0.23		–0.20	0.08		0.52	0.52
2025	12		0.24		0.50				–0.13	0.40			0.02		0.42			–0.33		0.48	0.20

[a] Recognizable Taxonomic Units (RTUs) abbreviated as follows: Agro, *Agromyza alnibetulae;* Cole, *Coleophora* spp.; Colo, *Colocasia coryli;* Depo, *Deporaus betulae;* Drep, Drepanidae; Ecto, *Ectoedemia minimella;* Epir, *Epirrita autumnata;* Espa, *Eriocrania sparrmannella;* Erio, *Eriocrania* spp.; Geom, Geometridae; Incu, *Incurvaria pectinea;* Roll, Leaf rollers; Sawf, Leaf-chewing sawflies; Mine, Leafmining sawflies; Noto, *Notodonta dromedarius;* Orch, *Orchestes* spp.; Paro, *Parornix* spp.; Pcor, *Phyllonorycter corylifoliella;* Pbis, *Phylloporia bistrigella;* Stig, *Stigmella* spp.
[b] Empty cells indicate the absence of RTU during the study year. Boldface indicates statistically significant correlations ($P < 0.05$).

Table S7
Spearman rank correlation coefficients (r_S) between site-specific herbivore diversity (Shannon index) and distance from the polluter.

Year	Volkhov			Syasstroy		
	r_S	n	P	r_S	n	P
1988	–0.657	6	0.16			
1989				0.071	8	0.87
1990	0.270	14	0.33	–0.310	8	0.46
1992	–0.125	15	0.66	–0.196	12	0.54
1993	–0.018	15	0.95	0.091	12	0.78
1994	0.318	15	0.25	0.378	12	0.23
1995	0.004	15	0.99	0.476	12	0.12
1996	0.293	15	0.29	0.112	12	0.73
1997	0.086	15	0.76	–0.182	12	0.57
1998	0.032	15	0.91	–0.119	12	0.71
1999	0.411	15	0.13	0.014	12	0.97
2000	–0.146	15	0.60	–0.098	12	0.76
2001	–0.489	15	0.06	0.287	12	0.37
2002	–0.504	15	0.06	0.287	12	0.37
2003	–0.536	15	0.04	–0.217	12	0.50
2004	–0.136	15	0.63	–0.210	12	0.51
2005	–0.307	15	0.27	0.136	11	0.69
2017	0.248	14	0.39	–0.064	11	0.85
2025	–0.252	12	0.43	–0.248	10	0.49

Table S8
Spearman rank correlation coefficients (r_S) between site-specific herbivore [a] densities and study year [b] around Volkhov.

Site	*n*	Agro	Cole	Colo	Depo	Drep	Ecto	Epir	Espa	Erio	Geom	Incu	Roll	Sawf	Mine	Noto	Orch	Paro	Pcor	Pbis	Stig
0.4W	16	0.20	–0.12		**0.51**				0.31	–0.50	–0.02	0.00	0.27	0.20	0.34			–0.02			–0.22
1.4W	18	0.12	0.22		**0.51**		0.14		0.35	**–0.71**	0.12	–0.07	0.11	–0.16	–0.11			0.05		0.16	–0.13
1.8W	19	0.02	–0.11		0.23		–0.04	0.37		**–0.84**	–0.11	–0.04	–0.16	–0.30	0.12	–0.30	0.34	–0.27			–0.23
1.9S	18		–0.02		–0.09				0.35	**–0.76**		–0.02	–0.28	0.26	0.20			–0.39		0.21	0.03
2.6W	17	0.15	0.11	0.41	0.03		0.20			**–0.67**	–0.15	–0.05	–0.05	0.26	**0.50**	0.36		**–0.48**	–0.31		–0.01
3.5W	18	0.12	0.13		0.06		–0.41			**–0.58**			0.06		–0.17			**0.00**			0.07
4.2W	16	–0.25	–0.05		–0.02				0.42	**–0.77**		–0.10	–0.03		–0.11			–0.16			0.12
4.3S	19	–0.21	0.24	0.04	**0.77**					**–0.75**	0.34	–0.03	–0.41	**0.47**	0.22			–0.36			–0.15
8.5N	19	–0.10	0.04		0.17			–0.16		–0.32	0.00	0.30	**–0.56**	0.34	0.22			**–0.66**		**0.47**	–0.33
9.4S	19		0.15		0.28	–0.16	–0.26		–0.07	**–0.56**	–0.09	–0.03	**–0.51**		0.07	–0.39	0.35	**–0.47**		0.30	0.11
11.6N	19	–0.27	**0.59**		0.03		–0.22	–0.37		–0.39	0.37		–0.40	0.34	0.08		0.04	–0.22		0.32	–0.09
13.0S	18	–0.16	0.14		0.26					**–0.61**	0.11	–0.02	–0.22		–0.07		0.07	**–0.63**		0.26	0.13
13.9N	16	–0.31	0.25		0.35	–0.25	–0.36	–0.46	–0.14	–0.48	0.36	0.31	0.09	0.36	0.36	–0.08	0.31	–0.34		**0.56**	–0.25
15.3S	19		0.11		0.10	–0.17				**–0.68**	0.27	–0.04	–0.42	–0.30	0.17		0.13	**–0.58**	–0.04	**0.69**	0.00
15.9N	19	–0.07	0.41		0.41		0.21			**–0.63**	0.34	–0.11	–0.24		0.41			–0.32		**0.46**	–0.32

[a] Recognizable Taxonomic Units (RTUs) abbreviated as follows: Agro, *Agromyza alnibetulae;* Cole, *Coleophora* spp.; Colo, *Colocasia coryli;* Depo, *Deporaus betulae;* Drep, Drepanidae; Ecto, *Ectoedemia minimella;* Epir, *Epirrita autumnata;* Espa, *Eriocrania sparrmannella;* Erio, *Eriocrania* spp.; Geom, Geometridae; Incu, *Incurvaria pectinea;* Roll, Leaf rollers; Sawf, Leaf-chewing sawflies; Mine, Leafmining sawflies; Noto, *Notodonta dromedarius;* Orch, *Orchestes* spp.; Paro, *Parornix* spp.; Pcor, *Phyllonorycter corylifoliella;* Pbis, *Phylloporia bistrigella;* Stig, *Stigmella* spp.

[b] Empty cells indicate the absence of RTU during the study year. Boldface indicates statistically significant correlations ($P < 0.05$).

Table S9
Spearman rank correlation coefficients (r_S) between site-specific herbivore [a] densities and study year [b] around Syasstroy.

Site	*n*	Agro	Cole	Colo	Depo	Drep	Ecto	Epir	Espa	Erio	Geom	Incu	Roll	Sawf	Mine	Noto	Orch	Paro	Pcor	Pbis	Stig
0.3N	18	–0.07	0.10	0.35	–0.27	0.35	0.03	–0.08	0.24	**–0.64**		–0.02	–0.18	0.45	0.28			–0.28			0.38
2.7W	18	–0.40	0.08		0.15	–0.30	–0.26	–0.31	**0.55**	**–0.55**		–0.02	0.19	0.45	0.28			–0.38			**–0.66**
3.2N	15	–0.10	–0.13		–0.27				–0.12	–0.45	0.19	0.31	–0.16	–0.37	0.42		0.25	**–0.53**			0.00
3.5W	18	0.38	–0.02		0.19		–0.10	–0.31		**–0.86**	0.02	0.19	0.34	0.07	0.11	0.30	0.28	–0.26		0.46	0.31
5.0N	15	0.06	0.43		0.46		0.33	–0.24	0.13	**–0.69**	0.43	0.10	0.31	0.12	0.16	–0.06	0.59	–0.40		0.37	0.04
5.2W	17	0.12	0.03		0.19	0.10	0.03	–0.31		**–0.85**	0.24	0.07	0.35	0.44	0.40		0.00	–0.04		0.31	0.01
7.6W	18	–0.18	0.06	0.36	0.08		–0.20	–0.31	–0.36	**–0.70**	–0.03	0.26	0.15	0.14	–0.11	–0.02	0.10	0.09		0.46	–0.18
8.6N	18	–0.02	0.11		–0.09	–0.30	–0.07	0.25	0.19	**–0.84**		0.26	–0.04	0.35	0.14		0.26	0.12			–0.31
9.6W	17	0.06	0.01		0.22		0.09		0.09	**–0.69**	0.31	0.06	0.25	**0.56**	**0.35**		0.00	**–0.49**		–0.10	–0.13
11.7W	18	0.34	**–0.19**	0.35	0.30	–0.12	0.01	–0.08	0.36	**–0.86**	–0.38	0.25	0.14	0.45	–0.06		0.00	**–0.63**		**0.49**	**–0.12**
12.8W	17	–0.13	–0.14		0.42		0.00		0.18	**–0.74**	–0.09	–0.05	0.14	**0.49**	0.31		0.00	–0.37		0.42	–0.34
14.2N	18		0.32	0.36	–0.04		–0.10	–0.33	0.31	**–0.71**	0.40		–0.36	–0.28	–0.31			–0.35			–0.16

[a] Recognizable Taxonomic Units (RTUs) abbreviated as follows: Agro, *Agromyza alnibetulae;* Cole, *Coleophora* spp.; Colo, *Colocasia coryli;* Depo, *Deporaus betulae;* Drep, Drepanidae; Ecto, *Ectoedemia minimella;* Epir, *Epirrita autumnata;* Espa, *Eriocrania sparrmannella;* Erio, *Eriocrania* spp.; Geom, Geometridae; Incu, *Incurvaria pectinea;* Roll, Leaf rollers; Sawf, Leaf-chewing sawflies; Mine, Leafmining sawflies; Noto, *Notodonta dromedarius;* Orch, *Orchestes* spp.; Paro, *Parornix* spp.; Pcor, *Phyllonorycter corylifoliella;* Pbis, *Phylloporia bistrigella;* Stig, *Stigmella* spp.

[b] Empty cells indicate the absence of RTU during the study year. Boldface indicates statistically significant correlations ($P < 0.05$).

Table S10
Spearman rank correlation coefficients (r_S) between herbivore diversity (Shannon index) and study year.

Polluter	Site code [a]	r_S	n	P
Volkhov	15.9 N	0.211	17	0.42
	13.9 N	0.250	16	0.35
	11.6 N	–0.110	17	0.67
	8.5 N	0.100	18	0.69
	4.2 W	0.434	17	0.08
	3.5 W	0.221	17	0.39
	2.6 W	0.362	16	0.17
	1.8 W[b]	0.418	18	0.08
	1.4 W	0.243	17	0.35
	0.4 W[c]	0.571	15	0.03
	1.9 S	0.292	17	0.26
	4.3 S	0.550	18	0.02
	9.4 S	0.358	18	0.14
	13.0 S[d]	0.061	17	0.82
	15.3 S	0.096	18	0.70
Syasstroy	12.8 W	0.245	17	0.34
	11.7 W	0.608	18	0.01
	9.6 W	0.532	17	0.03
	7.6 W	0.604	18	0.01
	5.2 W	0.395	17	0.12
	3.5 W	0.404	18	0.10
	2.7 W	0.263	18	0.29
	0.3 N	0.484	18	0.04
	3.2 N[e]	–0.037	14	0.90
	5.0 N[f]	0.525	15	0.04
	8.6 N	0.341	17	0.18
	14.2 N	0.349	17	0.17

Table S11
Sources of variation in damage of birch foliage around Volkhov and Syasstroy (SAS GLIMMIX procedure, type III sum of squares).

Effect type	Source of variation	Statistics	P
Fixed	Polluter	$F_{1, 3.8} = 2.36$	0.20
	Year	$F_{2, 175.6} = 9.12$	0.0002
	Pollution load	$F_{1, 3.8} = 40.2$	0.0038
	Polluter × Year	$F_{2, 175.6} = 2.86$	0.06
	Polluter × Pollution load	$F_{1, 3.8} = 2.90$	0.17
	Pollution load × Year	$F_{2, 175.6} = 3.29$	0.04
	Polluter × Pollution load × Year	$F_{2, 175.6} = 0.44$	0.64
Random	Site (Polluter)	$\chi^2_1 = 0.08$	0.39

Data S1

Results of population counts of insect herbivores feeding on foliage of *Betula pubescens* in the surroundings of Volkhov and Syasstroy, 1988–2025.

Explanations of columns:

1. Year;
2. Census: 1, late Juny – early July; 2, mid August to late September;
3. Pollution gradient (Volkhov or Syasstroy);
4. Distance from the polluter to the study site (for site co-ordinates consult Table S2);
5. Compass direction from the polluter to the study site;
6. Number of birch trees or branches;
7. Estimated leaf area of surveyed birch trees, m^2;
8. *Eriocrania* spp., number of mines;
9. Leaf rollers, number of larval shelters;
10. *Stigmella* spp., number of mines;
11. *Parornix* spp., number of mines;
12. *Deporaus betulae*, number of rolls;
13. *Epirrita autumnata*, number of larvae;
14. Leaf-mining sawflies, number of mines;
15. *Agromyza alnibetulae*, number of mines;
16. *Coleophora* spp., number of mines;
17. Drepanidae, number of larvae;
18. *Incurvaria pectinea*, number of mines;
19. Geometridae, number of larvae;
20. *Colocasia coryli*, number of larvae;
21. Psychidae, number of larvae;
22. *Notodonta dromedarius*, number of larvae;
23. Leaf-chewing sawflies, number of larvae;
24. *Eriocrania sparrmannella*, number of mines;
25. *Ectoedemia minimella*, number of mines;
26. *Achlya flavicornis*, number of larvae;
27. *Phylloporia bistrigella*, number of mines;
28. *Orchestes* spp., number of mines;
29. *Apoderus coryli*, number of larvae;
30. *Bucculatrix demaryella*, number of mines;
31. *Phyllonorycter corylifoliellus*, number of mines;

```
1988 2 Syasstro 0.3 N 10 0.66759 . 6 . 6 . . . . . . . . . . . . . . . . . . . 0
1988 2 Syasstro 2.7 W 10 0.64168 . 0 . 2 . . . . . . . . . . . . . . . . . . . 0
1988 2 Syasstro 3.5 W 10 0.69104 . 0 . 1 . . . . . . . . . . . . . . . . . . . 0
1988 2 Syasstro 5.2 W 10 0.68117 . 4 . 0 . . . . . . . . . . . . . . . . . . . 0
1988 2 Syasstro 7.6 W 10 0.78606 . 2 . 0 . . . . . . . . . . . . . . . . . . . 0
1988 2 Syasstro 9.6 W 10 0.66513 . 0 . 5 . . . . . . . . . . . . . . . . . . . 0
1988 2 Syasstro 11.7 W 10 0.79840 . 1 . 7 . . . . . . . . . . . . . . . . . . . 0
1988 2 Syasstro 12.8 W 10 0.65032 . 1 . 7 . . . . . . . . . . . . . . . . . . . 0
1988 2 Volkhov 1.8 W 11 0.88478 0 30 2 28 0 0 0 23 0 0 0 0 0 0 0 0 0 0 0 0 0 0 0 0
1988 2 Volkhov 8.5 N 24 1.68071 0 92 1 62 0 0 0 4 0 0 0 0 0 0 0 0 0 0 0 0 0 0 0 0
1988 2 Volkhov 4.3 S 6 0.34058 0 13 2 63 0 0 0 9 0 0 0 0 0 0 0 0 0 0 0 0 0 0 0 0
1988 2 Volkhov 11.6 N 25 1.54003 1 59 0 49 0 . 0 12 0 0 0 0 0 1 0 0 0 0 0 0 0 0 0 0
1988 2 Volkhov 9.4 S 12 0.88848 0 41 0 113 2 0 0 0 1 0 0 0 0 0 1 0 0 0 0 0 0 0 0 0
1988 2 Volkhov 13.0 S 10 0.83295 0 31 0 64 0 0 0 0 1 0 0 0 0 0 0 0 0 0 0 0 0 0 0 0
1988 2 Volkhov 15.9 N 19 1.77696 2 24 4 24 0 . 0 1 8 0 0 0 0 0 0 0 0 0 0 0 0 0 0 0
1988 2 Volkhov 15.3 S 14 0.69351 0 24 2 16 0 0 0 0 0 0 0 0 0 0 0 0 0 0 0 0 0 0 0 0
1989 1 Syasstro 0.3 N 10 0.43807 2 5 0 0 1 0 0 0 286 0 0 0 0 0 0 0 0 0 0 0 0 0 0 0
1989 1 Syasstro 2.7 W 10 0.42943 204 1 0 0 1 0 0 0 182 0 0 0 0 0 0 0 0 0 0 0 0 0 0 0
1989 1 Syasstro 3.5 W 10 0.56887 44 4 0 0 37 0 0 0 137 0 0 0 0 0 0 0 0 0 0 0 0 0 0 0
1989 1 Syasstro 5.2 W 10 0.51458 146 2 0 1 3 0 0 0 123 0 0 0 0 0 0 0 0 0 0 0 0 0 0 0
1989 1 Syasstro 7.6 W 10 0.53432 24 0 0 0 11 0 0 0 310 0 0 0 0 0 0 0 0 0 0 0 0 0 0 0
1989 1 Syasstro 9.6 W 10 0.51705 12 0 0 0 30 0 0 0 262 0 0 0 0 0 0 0 0 0 0 0 0 0 0 0
1989 1 Syasstro 11.7 W 10 0.53679 62 4 0 1 2 0 0 0 321 0 0 0 0 0 0 3 0 0 0 0 0 0 0 0
```

1989 1 Syasstro 12.8 W 10 0.56270 33 3 1 0 0 0 0 0 86 0 0 0 0 0 0 0 0 0 0 0 0 0 0 0
1989 2 Syasstro 0.3 N 10 0.77742 19 36 0 1 10 0 0 0 . 0 0 0 0 0 0 0 0 0 0 0 0 0 0 0
1989 2 Syasstro 2.7 W 10 0.76631 48 12 1 1 19 0 0 1 . 0 0 0 0 0 0 0 0 0 0 0 0 0 0 0
1989 2 Syasstro 3.2 N 10 0.56147 10 16 0 7 0 0 0 0 . 0 0 0 0 0 0 0 0 0 0 0 0 0 0 0
1989 2 Syasstro 5.2 W 10 0.60343 208 4 1 1 15 0 0 0 . 0 0 0 0 0 0 0 0 0 0 0 0 0 0 0
1989 2 Syasstro 8.6 N 10 0.60836 31 13 1 0 51 0 0 0 . 0 0 0 0 0 0 0 0 0 0 0 0 0 0 0
1989 2 Syasstro 9.6 W 10 0.49483 39 6 3 0 18 0 0 0 . 0 0 0 0 0 0 0 0 0 0 0 0 0 0 0
1989 2 Syasstro 12.8 W 10 0.62934 23 10 2 12 0 0 0 0 . 0 0 0 0 0 0 0 0 0 0 0 0 0 0 0
1989 2 Syasstro 14.2 N 10 0.71942 12 14 2 2 19 0 0 0 . 0 0 0 0 0 0 0 0 0 0 0 0 0 0 0
1989 2 Volkhov 0.4 W 10 0.64662 0 1 1 0 0 0 0 0 . 0 0 0 0 0 0 0 0 0 0 0 0 0 0 0
1989 2 Volkhov 1.4 W 10 0.54543 0 2 0 0 0 0 0 0 . 0 0 0 0 0 0 0 0 0 0 0 0 0 0 0
1989 2 Volkhov 1.8 W 10 0.48126 0 3 0 1 4 0 0 0 . 0 0 0 0 0 0 0 0 0 0 0 0 0 0 0
1989 2 Volkhov 2.6 W 10 0.47879 0 2 0 2 1 0 0 0 . 0 0 0 0 0 0 0 0 0 0 0 0 0 0 0
1989 2 Volkhov 1.9 S 10 0.67870 0 2 0 3 0 0 0 0 . 0 0 0 0 0 0 0 0 0 0 0 0 0 0 0
1989 2 Volkhov 3.5 W 10 0.71572 0 10 1 0 0 0 0 0 . 0 0 0 0 0 0 0 0 0 0 0 0 0 0 0
1989 2 Volkhov 8.5 N 10 0.71202 48 35 1 9 32 0 0 3 . 0 0 0 0 0 0 0 0 0 0 0 0 0 0 0
1989 2 Volkhov 4.3 S 10 0.54049 0 10 0 1 0 0 0 0 . 0 0 0 0 0 0 0 0 0 0 0 0 0 0 0
1989 2 Volkhov 11.6 N 10 0.73917 10 25 0 0 3 0 0 3 . 0 0 0 0 0 0 0 0 0 0 0 0 0 0 0
1989 2 Volkhov 9.4 S 10 0.82555 0 36 0 6 0 0 0 0 . 0 0 0 0 0 0 0 0 0 0 0 0 0 0 0
1989 2 Volkhov 13.0 S 10 0.52939 0 7 0 3 0 0 0 0 . 0 0 0 0 0 0 0 0 0 0 0 0 0 0 0
1989 2 Volkhov 15.9 N 10 1.08962 6 33 1 4 2 0 0 0 . 0 0 0 0 0 0 0 0 0 0 0 0 0 0 0
1989 2 Volkhov 15.3 S 5 0.35786 0 2 0 1 4 0 0 0 . 0 0 0 0 0 0 0 0 0 0 0 0 0 0 0
1990 2 Syasstro 0.3 N 10 1.27719 0 32 0 3 8 0 23 0 46 0 0 0 0 0 0 0 0 0 0 0 0 0 0 0
1990 2 Syasstro 2.7 W 10 0.89835 0 23 9 8 19 0 37 0 8 0 0 0 0 0 0 0 0 0 0 0 0 0 0 0
1990 2 Syasstro 3.2 N 10 0.55900 0 24 0 8 16 0 5 0 32 0 0 0 0 0 0 1 0 0 0 0 0 0 0 0
1990 2 Syasstro 3.5 W 10 0.84035 0 11 0 8 3 0 19 0 19 0 0 0 0 0 0 0 0 0 0 0 0 0 0 0
1990 2 Syasstro 7.6 W 10 0.83542 0 12 0 1 35 0 5 0 0 0 0 2 0 0 0 0 0 0 0 0 0 0 0 0
1990 2 Syasstro 8.6 N 10 0.59109 0 18 0 7 53 0 18 0 3 0 0 0 0 0 0 0 0 0 0 0 0 0 0 0
1990 2 Syasstro 11.7 W 10 0.61577 0 3 0 9 0 0 42 0 0 0 0 2 0 0 0 0 0 0 0 0 0 0 0 0
1990 2 Syasstro 14.2 N 10 0.95635 0 12 0 2 7 0 12 0 4 0 0 0 0 0 0 1 0 0 0 0 0 0 0 0
1990 2 Volkhov 0.4 W 10 0.58738 0 1 0 0 2 0 13 0 43 0 0 0 0 0 0 0 0 0 0 0 0 0 0 0
1990 2 Volkhov 1.4 W 10 0.63798 0 5 0 6 5 0 17 0 121 0 0 0 0 0 0 0 0 0 0 0 0 0 0 0
1990 2 Volkhov 1.8 W 10 0.53556 0 11 0 4 20 0 17 0 124 0 0 10 0 0 1 1 0 0 1 0 0 0 0 0
1990 2 Volkhov 2.6 W 10 0.46769 11 6 0 4 0 0 0 0 121 0 0 0 0 0 0 0 0 0 0 0 0 0 0 0
1990 2 Volkhov 1.9 S 10 0.54296 0 5 1 1 3 0 9 0 30 0 0 0 0 0 0 0 0 0 0 0 0 0 0 0
1990 2 Volkhov 3.5 W 10 0.47509 0 0 0 0 1 0 1 0 93 0 0 0 0 0 0 0 0 0 0 0 0 0 0 0
1990 2 Volkhov 4.2 W 10 0.52692 0 7 0 0 2 0 7 0 86 0 0 0 0 0 0 0 0 0 0 0 0 0 0 0
1990 2 Volkhov 8.5 N 10 0.41339 0 11 10 17 1 0 38 0 32 0 0 0 0 0 0 0 0 0 0 0 0 0 0 0
1990 2 Volkhov 4.3 S 10 0.64415 3 2 1 0 0 0 5 0 226 0 0 0 0 0 0 0 0 0 0 0 0 0 0 0
1990 2 Volkhov 11.6 N 10 0.46522 0 7 0 7 4 0 7 0 57 0 0 0 0 0 0 0 0 0 0 0 0 0 0 0
1990 2 Volkhov 9.4 S 10 0.51211 0 18 0 0 39 0 6 0 111 0 0 0 0 0 0 0 0 0 0 0 0 0 0 0
1990 2 Volkhov 13.0 S 10 0.39488 0 13 0 12 34 0 28 0 29 0 0 0 0 0 0 0 0 0 0 0 0 0 0 0
1990 2 Volkhov 15.9 N 10 0.45658 0 5 0 1 66 0 0 0 55 0 0 0 0 0 0 0 0 0 0 0 0 0 0 0
1990 2 Volkhov 15.3 S 10 0.63674 0 18 0 3 23 0 10 0 150 0 0 0 0 0 0 1 0 0 0 0 0 0 0 0
1991 1 Syasstro 0.3 N 10 0.66142 126 . . . .
1991 1 Syasstro 2.7 W 10 0.70955 95 . . . .
1991 1 Syasstro 3.5 W 10 0.52322 327 . . . .
1991 1 Syasstro 5.2 W 10 0.64045 374 . . . .
1991 1 Syasstro 7.6 W 10 0.62811 120 . . . .
1991 1 Syasstro 9.6 W 10 0.56641 74 . . . .
1991 1 Syasstro 11.7 W 10 0.72066 177 . . . .
1991 1 Syasstro 12.8 W 10 0.69844 141 . . . .
1992 1 Syasstro 0.3 N 10 0.68117 390 0
1992 1 Syasstro 2.7 W 10 1.15502 32 0
1992 1 Syasstro 3.2 N 10 1.31298 33 0
1992 1 Syasstro 3.5 W 10 0.60466 199 0
1992 1 Syasstro 5.0 N 10 0.94771 56 0
1992 1 Syasstro 5.2 W 10 0.68240 260 0
1992 1 Syasstro 7.6 W 10 0.96746 39 0
1992 1 Syasstro 8.6 N 10 0.76261 247 0
1992 1 Syasstro 9.6 W 10 1.21055 117 0
1992 1 Syasstro 11.7 W 10 0.65155 172 0
1992 1 Syasstro 12.8 W 10 0.82308 285 0
1992 1 Syasstro 14.2 N 10 1.16366 258 0
1992 1 Volkhov 0.4 W 10 1.26238 27 0
1992 1 Volkhov 1.4 W 10 1.49067 140 0
1992 1 Volkhov 1.8 W 10 0.84159 173 0
1992 1 Volkhov 1.9 S 10 2.56302 40 0
1992 1 Volkhov 3.5 W 10 2.31622 11 0
1992 1 Volkhov 4.2 W 10 1.53880 26 0
1992 1 Volkhov 8.5 N 10 0.53062 91 0
1992 1 Volkhov 4.3 S 10 2.01389 135 0
1992 1 Volkhov 11.6 N 10 0.46275 210 0
1992 1 Volkhov 9.4 S 10 1.39689 18 0
1992 1 Volkhov 13.0 S 10 0.93167 96 0
1992 1 Volkhov 15.9 N 10 1.41910 87 0
1992 1 Volkhov 15.3 S 10 1.15502 252 0
1992 2 Syasstro 0.3 N 10 2.74442 31 4 0 3 1 0 0 0 0 0 0 0 0 0 0 0 0 0 0 0 0 0 0 0
1992 2 Syasstro 2.7 W 10 2.08423 3 3 1 1 0 0 0 0 0 1 0 0 0 0 0 0 0 1 0 0 0 0 0 0
1992 2 Syasstro 3.2 N 10 2.60251 0 3 0 8 0 0 0 0 0 0 0 0 0 0 0 0 0 0 0 0 0 0 0 0
1992 2 Syasstro 3.5 W 10 1.58569 1 0 1 4 2 0 0 0 0 0 0 0 0 0 0 0 0 0 0 0 0 0 0 0
1992 2 Syasstro 5.0 N 10 2.29894 0 0 2 10 8 0 0 0 0 0 0 0 0 0 0 0 0 0 0 0 0 0 0 0
1992 2 Syasstro 5.2 W 10 1.99785 2 1 2 2 16 0 0 0 0 0 0 0 0 0 0 0 0 0 0 0 0 0 0 0
1992 2 Syasstro 7.6 W 10 2.40630 3 0 4 3 0 0 0 0 0 0 0 0 0 0 0 0 1 1 0 0 0 0 0 0
1992 2 Syasstro 8.6 N 10 2.48034 2 3 1 4 1 0 0 0 0 3 0 0 0 0 0 0 0 0 0 0 0 0 0 0
1992 2 Syasstro 9.6 W 10 1.37961 1 5 1 7 0 0 0 0 0 0 0 0 0 0 0 0 0 0 0 0 0 0 0 0
1992 2 Syasstro 11.7 W 10 1.94232 0 2 5 4 6 0 0 0 0 0 0 0 0 0 0 0 0 0 0 0 0 0 0 0
1992 2 Syasstro 12.8 W 10 2.01636 17 4 8 5 0 0 0 0 0 0 0 0 0 0 0 0 0 0 0 0 0 0 0 0

```
1992 2 Syasstro 14.2 N 10 3.17632 1 4 0 5 0 0 0 0 0 0 0 0 0 0 0 0 0 0 0 0 0 0 0 0
1992 2 Volkhov 0.4 W 10 2.15950 5 1 1 23 0 0 0 0 0 0 0 0 0 0 0 0 0 0 0 0 0 0 0 0
1992 2 Volkhov 1.4 W 10 3.43546 1 0 5 6 0 0 0 0 0 0 0 0 0 0 0 0 0 0 0 0 0 0 0 0
1992 2 Volkhov 1.8 W 10 2.20146 20 0 6 13 2 0 0 0 0 0 0 0 0 0 0 0 0 0 0 0 0 0 0 0
1992 2 Volkhov 2.6 W 10 2.27673 7 5 3 7 0 0 0 0 0 0 0 0 0 0 0 0 0 0 0 0 0 0 0 1
1992 2 Volkhov 1.9 S 10 2.00772 2 1 0 9 0 0 0 0 0 0 0 0 0 0 0 0 0 0 0 0 0 0 0 0
1992 2 Volkhov 3.5 W 10 2.18665 8 4 1 22 0 0 0 0 0 0 0 0 0 0 0 0 0 1 0 0 0 0 0 0
1992 2 Volkhov 4.2 W 10 1.21796 0 1 0 9 2 0 0 0 0 0 0 0 0 0 0 0 0 0 0 0 0 0 0 0
1992 2 Volkhov 8.5 N 10 2.08793 2 0 0 13 1 0 0 0 0 0 0 0 0 0 0 0 0 0 0 0 0 0 0 0
1992 2 Volkhov 4.3 S 10 2.14099 13 4 0 10 0 0 0 0 0 0 0 0 0 0 0 0 0 0 0 0 0 0 0 0
1992 2 Volkhov 11.6 N 10 2.47540 11 2 2 3 0 0 0 0 0 0 0 0 0 0 0 0 0 0 0 0 0 0 0 0
1992 2 Volkhov 9.4 S 10 2.43715 0 0 1 61 0 0 0 0 0 0 0 0 0 0 0 0 0 1 0 0 0 0 0 0
1992 2 Volkhov 13.9 N 10 2.9900 1 2 3 10 3 0 0 0 0 0 0 0 0 0 0 0 0 0 0 0 0 0 0 0
1992 2 Volkhov 13.0 S 10 1.3068 1 0 1 5 0 0 0 0 0 0 0 0 0 0 0 0 0 0 0 0 0 0 0 0
1992 2 Volkhov 15.9 N 10 2.1373 4 0 5 8 0 0 0 0 0 0 0 0 0 0 0 0 0 0 0 0 0 0 0 0
1992 2 Volkhov 15.3 S 10 2.2570 0 1 5 11 13 0 0 0 0 0 0 0 0 0 0 0 0 0 0 0 0 0 0 0
1993 1 Syasstro 0.3 N 10 1.1600 410 0 0 0 0 0 0 0 0 0 0 0 0 0 0 0 0 0 0 0 0 0 0 0
1993 1 Syasstro 2.7 W 10 1.3216 96 0 0 0 0 0 0 0 0 0 0 0 0 0 0 0 0 0 0 0 0 0 0 0
1993 1 Syasstro 3.2 N 10 1.4882 261 0 0 0 0 0 0 0 0 0 0 0 0 0 0 0 0 0 0 0 0 0 0 0
1993 1 Syasstro 3.5 W 10 1.0304 133 0 0 0 0 0 0 0 0 0 0 0 0 0 0 0 0 0 0 0 0 0 0 0
1993 1 Syasstro 5.0 N 10 0.9773 190 0 0 0 0 0 0 0 0 0 0 0 0 0 0 0 0 0 0 0 0 0 0 0
1993 1 Syasstro 5.2 W 10 1.0884 180 0 0 0 0 0 0 0 0 0 0 0 0 0 0 0 0 0 0 0 0 0 0 0
1993 1 Syasstro 7.6 W 10 0.8984 144 0 0 0 0 0 0 0 0 0 0 0 0 0 0 0 0 0 0 0 0 0 0 0
1993 1 Syasstro 8.6 N 10 1.5166 146 0 0 0 0 0 0 0 0 0 0 0 0 0 0 0 0 0 0 0 0 0 0 0
1993 1 Syasstro 9.6 W 10 0.8613 164 0 0 0 0 0 0 0 0 0 0 0 0 0 0 0 0 0 0 0 0 0 0 0
1993 1 Syasstro 11.7 W 10 1.2328 222 0 0 0 0 0 0 0 0 0 0 0 0 0 0 0 0 0 0 0 0 0 0 0
1993 1 Syasstro 12.8 W 10 1.1982 525 1 0 0 0 0 0 0 0 0 0 0 0 0 0 0 0 0 0 0 0 0 0 0
1993 1 Syasstro 14.2 N 10 1.3204 55 0 0 0 0 0 0 0 0 0 0 0 0 0 0 0 0 0 0 0 0 0 0 0
1993 1 Volkhov 0.4 W 11 1.6165 146 0 0 0 0 0 0 0 0 0 0 0 0 0 0 0 0 0 0 0 0 0 0 0
1993 1 Volkhov 1.4 W 10 1.3204 230 1 0 0 0 0 0 0 0 0 0 0 0 0 0 0 0 0 0 0 0 0 0 0
1993 1 Volkhov 1.8 W 10 1.5869 255 0 0 0 0 0 0 0 0 0 0 0 0 0 0 0 0 0 0 0 0 0 0 0
1993 1 Volkhov 2.6 W 10 1.5005 116 0 0 0 0 0 0 0 0 0 0 0 0 0 0 0 0 0 0 0 0 0 0 0
1993 1 Volkhov 1.9 S 10 1.4351 49 2 0 0 0 0 0 0 0 0 0 0 0 0 0 0 0 0 0 0 0 0 0 0
1993 1 Volkhov 3.5 W 10 1.7757 48 4 0 0 0 0 0 0 0 0 0 0 0 0 0 0 0 0 0 0 0 0 0 0
1993 1 Volkhov 4.2 W 10 1.8473 210 1 0 0 0 0 0 0 0 0 0 0 0 0 0 0 0 0 0 0 0 0 0 0
1993 1 Volkhov 8.5 N 10 2.0114 116 0 0 0 0 0 0 0 0 0 0 0 0 0 0 0 0 0 0 0 0 0 0 0
1993 1 Volkhov 4.3 S 10 1.4796 212 0 0 0 0 0 0 0 0 0 0 0 0 0 0 0 0 0 0 0 0 0 0 0
1993 1 Volkhov 11.6 N 10 1.4648 85 6 0 0 0 0 0 0 0 0 0 0 0 0 0 0 0 0 0 0 0 0 0 0
1993 1 Volkhov 9.4 S 10 1.3549 100 3 0 0 0 0 0 0 0 0 0 0 0 0 0 0 0 0 0 0 0 0 0 0
1993 1 Volkhov 13.9 N 10 1.7782 88 8 0 0 0 0 0 0 0 0 0 0 0 0 0 0 0 0 0 0 0 0 0 0
1993 1 Volkhov 13.0 S 10 1.5573 116 0 0 0 0 0 0 0 0 0 0 0 0 0 0 0 0 0 0 0 0 0 0 0
1993 1 Volkhov 15.9 N 10 1.9238 423 0 0 0 0 0 0 0 0 0 0 0 0 0 0 0 0 0 0 0 0 0 0 0
1993 1 Volkhov 15.3 S 10 0.7182 388 0 0 0 0 0 0 0 0 0 0 0 0 0 0 0 0 0 0 0 0 0 0 0
1993 2 Syasstro 0.3 N 25 7.8803 . 1 2 1 0 0 0 0 0 0 0 0 0 0 0 0 0 0 0 0 0 0 0 0
1993 2 Syasstro 2.7 W 25 7.2362 . 0 0 0 0 0 0 0 0 0 0 0 0 0 0 0 0 0 0 0 0 0 0 0
1993 2 Syasstro 3.2 N 25 7.7828 . 0 5 0 0 0 0 0 0 0 0 0 0 0 0 0 0 0 0 0 0 0 0 0
1993 2 Syasstro 3.5 W 25 4.8496 . 1 0 0 0 0 0 0 0 0 0 0 0 0 0 0 0 0 0 0 0 0 0 0
1993 2 Syasstro 5.0 N 26 8.1395 . 1 6 1 0 0 0 0 0 0 0 0 0 0 0 0 0 0 0 0 0 0 0 0
1993 2 Syasstro 5.2 W 25 7.2115 . 1 3 3 0 0 0 0 0 0 0 0 0 0 0 0 0 0 0 0 0 0 0 0
1993 2 Syasstro 7.6 W 25 7.3016 . 0 10 2 0 0 0 0 0 0 0 0 0 0 0 0 0 0 0 0 0 0 0 0
1993 2 Syasstro 8.6 N 25 8.7614 . 1 0 2 0 0 0 0 0 0 0 0 0 0 0 0 0 0 0 0 0 0 0 0
1993 2 Syasstro 9.6 W 25 8.1753 . 0 3 0 0 0 0 0 0 0 0 0 0 0 0 0 0 0 0 0 0 0 0 0
1993 2 Syasstro 11.7 W 25 6.9178 . 1 4 1 0 0 0 0 0 0 0 0 0 0 0 0 0 0 0 0 0 0 0 0
1993 2 Syasstro 12.8 W 25 9.0748 . 0 6 0 0 0 0 0 0 0 0 0 0 0 0 0 0 0 0 0 0 0 0 0
1993 2 Volkhov 0.4 W 25 12.6929 . 2 13 0 0 0 0 0 0 0 0 0 0 0 0 0 0 0 0 0 0 0 0 0
1993 2 Volkhov 1.4 W 24 8.7787 . 0 3 1 0 0 0 0 0 0 0 0 0 0 0 0 0 0 0 0 0 0 0 0
1993 2 Volkhov 1.8 W 25 6.2477 . 2 6 1 0 0 0 0 0 0 0 0 0 0 0 0 0 0 0 0 0 0 0 0
1993 2 Volkhov 2.6 W 25 7.9507 . 0 3 0 0 0 0 0 0 0 0 0 0 0 0 0 0 0 0 0 0 0 0 0
1993 2 Volkhov 1.9 S 25 8.5751 . 1 9 1 0 0 0 0 0 0 0 0 0 0 0 0 0 0 0 0 0 0 0 0
1993 2 Volkhov 3.5 W 25 4.3338 . 2 4 1 0 0 0 0 0 0 0 0 0 0 0 0 0 1 0 0 0 0 0 0
1993 2 Volkhov 4.2 W 13 4.3597 . 1 0 0 0 0 0 0 0 0 0 0 0 0 0 0 0 0 0 0 0 0 0 0
1993 2 Volkhov 8.5 N 37 10.0756 . 2 4 1 0 0 0 0 0 0 0 0 0 0 0 0 0 0 0 0 0 0 0 0
1993 2 Volkhov 4.3 S 25 10.0435 . 1 2 0 0 0 0 0 0 0 0 0 0 0 0 0 0 0 0 0 0 0 0 0
1993 2 Volkhov 11.6 N 25 8.8873 . 3 6 1 0 0 0 0 0 0 0 0 0 0 0 0 0 1 0 0 0 0 0 0
1993 2 Volkhov 13.9 N 25 11.5342 . 9 6 0 0 0 0 0 0 0 0 0 0 0 0 0 0 1 0 0 0 0 0 0
1993 2 Volkhov 13.0 S 25 11.4478 . 2 1 1 0 0 0 0 0 0 0 0 0 0 0 0 0 0 0 0 0 0 0 0
1993 2 Volkhov 15.9 N 25 11.7440 . 9 5 1 0 0 0 0 0 0 0 0 0 0 0 0 0 1 0 0 0 0 0 0
1993 2 Volkhov 15.3 S 25 11.1282 . 1 5 0 0 0 0 0 0 0 0 0 0 0 0 0 0 0 0 0 0 0 0 0
1994 1 Syasstro 0.3 N 15 0.6306 303 6 0 0 0 0 0 0 0 0 0 0 0 0 0 0 0 0 0 0 0 0 0
1994 1 Syasstro 2.7 W 15 0.9440 266 2 0 0 0 1 0 0 0 0 0 0 0 0 0 0 0 0 0 0 0 0 0 0
1994 1 Syasstro 3.2 N 15 1.2254 342 0 0 0 0 0 0 0 0 0 0 0 0 0 0 0 0 0 0 0 0 0 0 0
1994 1 Syasstro 3.5 W 15 0.9971 279 2 0 0 0 2 0 0 0 0 0 0 0 0 0 0 0 0 0 0 0 0 0 0
1994 1 Syasstro 5.0 N 15 1.9978 936 5 0 0 22 0 0 0 0 0 0 0 0 0 0 0 0 0 0 0 0 0 0 0
1994 1 Syasstro 5.2 W 15 1.7066 401 1 0 0 3 3 0 0 0 0 0 0 0 0 0 0 0 0 0 0 0 0 0 0
1994 1 Syasstro 7.6 W 15 1.6930 365 2 0 0 0 1 0 0 0 0 0 0 0 0 0 0 0 0 0 0 0 0 0 0
1994 1 Syasstro 8.6 N 15 2.6654 477 0 0 0 18 1 0 0 0 0 0 0 0 0 0 0 0 0 0 0 0 0 0 0
1994 1 Syasstro 9.6 W 15 1.4759 357 2 0 0 4 0 0 0 0 0 0 0 0 0 0 0 0 0 0 0 0 0 0 0
1994 1 Syasstro 11.7 W 15 1.7844 343 0 0 0 8 0 0 0 0 0 0 0 0 0 0 0 0 0 0 0 0 0 0 0
1994 1 Syasstro 12.8 W 15 1.5771 570 0 0 0 0 0 0 0 0 0 0 0 0 0 0 0 0 0 0 0 0 0 0 0
1994 1 Syasstro 14.2 N 15 0.9872 180 2 0 0 0 2 0 0 0 0 0 0 0 0 0 0 0 0 0 0 0 0 0 0
1994 1 Volkhov 0.4 W 15 1.2883 30 2 0 0 0 0 0 0 0 0 0 0 0 0 0 0 0 0 0 0 0 0 0
1994 1 Volkhov 1.4 W 15 1.3451 103 1 0 0 0 0 0 0 0 0 0 0 0 0 0 0 0 0 0 0 0 0 0
1994 1 Volkhov 1.8 W 15 1.7066 216 5 0 0 23 0 0 0 0 0 0 0 0 0 0 0 0 0 0 0 0 0 0 0
1994 1 Volkhov 2.6 W 15 1.3710 71 3 0 0 0 0 0 0 0 0 0 0 0 0 0 0 0 0 0 0 0 0 0
1994 1 Volkhov 1.9 S 15 1.2365 18 1 0 0 0 0 0 0 0 0 0 0 0 0 0 0 0 0 0 0 0 0 0
1994 1 Volkhov 3.5 W 15 1.5931 17 2 0 0 1 0 0 0 0 0 0 0 0 0 0 0 0 0 0 0 0 0 0 0
1994 1 Volkhov 4.2 W 15 1.3475 81 0 0 0 0 0 0 0 0 0 0 0 0 0 0 0 0 0 0 0 0 0 0 0
1994 1 Volkhov 8.5 N 15 1.1957 108 4 0 0 4 4 0 0 0 0 0 0 0 0 0 0 0 0 0 0 0 0 0 0
```

1994 1 Volkhov 4.3 S 15 0.8255 620 0 0 0 0 0 0 0 0 0 0 0 0 0 0 0 0 0 0 0 0 0 0 0
1994 1 Volkhov 11.6 N 15 1.6202 89 1 0 0 0 1 0 0 0 0 0 0 0 0 0 0 0 0 0 0 0 0 0 0
1994 1 Volkhov 9.4 S 15 0.9415 179 0 0 0 0 0 0 0 0 0 0 0 0 0 0 0 0 0 0 0 0 0 0 0
1994 1 Volkhov 13.9 N 15 1.3389 73 4 0 0 0 2 0 0 0 0 0 0 0 0 0 0 0 0 0 0 0 0 0 0
1994 1 Volkhov 13.0 S 15 2.6050 172 9 0 0 13 0 0 0 0 0 0 0 0 0 0 0 0 0 0 0 0 0 0 0
1994 1 Volkhov 15.9 N 15 0.8589 301 0 0 0 0 0 0 0 0 0 0 0 0 0 0 0 0 0 0 0 0 0 0 0
1994 1 Volkhov 15.3 S 15 2.15210 159 8 0 0 11 0 0 0 0 0 0 0 0 0 0 0 0 0 0 0 0 0 0 0
1994 2 Syasstro 0.3 N 10 0.83048 0 1 4 0 0 0 1 0 0 0 0 0 0 0 0 0 0 0 0 0 0 0 0 0
1994 2 Syasstro 2.7 W 10 0.68117 0 2 0 0 0 0 0 0 0 0 0 0 0 0 0 0 0 0 0 0 0 0 0 0
1994 2 Syasstro 3.2 N 10 1.36851 0 1 2 14 2 0 3 3 2 0 0 0 0 0 0 0 0 0 0 0 0 0 0 0
1994 2 Syasstro 3.5 W 10 1.59433 0 1 0 33 2 0 37 1 0 0 0 0 0 0 0 0 0 0 0 0 0 0 0 0
1994 2 Syasstro 5.0 N 10 1.61407 0 9 2 3 9 0 7 1 0 0 0 0 0 0 0 0 0 0 0 0 0 0 0 0
1994 2 Syasstro 5.2 W 10 1.04026 0 1 2 3 18 0 1 0 0 0 0 0 0 0 0 0 0 0 0 0 0 0 0 0
1994 2 Syasstro 7.6 W 10 1.65233 0 3 0 1 94 0 0 4 0 0 0 0 0 0 0 0 0 0 0 0 0 0 0 0
1994 2 Syasstro 8.6 N 10 1.57952 0 0 2 0 0 0 0 1 0 0 0 0 0 0 0 0 0 0 0 0 0 0 0 0
1994 2 Syasstro 9.6 W 10 1.36110 0 3 4 4 16 0 0 1 0 0 0 0 0 0 0 0 0 0 0 0 0 0 0 0
1994 2 Syasstro 11.7 W 10 1.53139 0 3 0 12 17 0 4 0 0 0 0 0 0 0 0 0 0 0 0 0 0 0 0 0
1994 2 Syasstro 12.8 W 10 1.18958 0 0 2 5 7 0 5 3 0 0 0 0 0 0 0 0 0 0 0 0 0 0 0 0
1994 2 Syasstro 14.2 N 10 2.00648 0 4 3 3 8 0 2 0 0 0 0 0 0 0 0 0 0 0 0 0 0 0 0 0
1994 2 Volkhov 0.4 W 10 0.69104 0 3 14 7 0 0 0 0 0 0 0 0 0 0 0 0 0 0 0 0 0 0 0 0
1994 2 Volkhov 1.4 W 10 0.80457 0 1 1 10 12 0 2 0 0 0 0 0 0 0 0 0 0 0 0 0 0 0 0 0
1994 2 Volkhov 1.8 W 10 1.56965 0 1 0 6 191 0 0 0 0 0 0 0 0 0 0 0 0 0 0 0 0 0 0 0
1994 2 Volkhov 2.6 W 10 1.63258 0 4 0 16 4 0 0 0 0 0 0 0 0 0 0 0 0 0 0 0 0 0 0 0
1994 2 Volkhov 1.9 S 10 0.74657 0 3 1 2 0 0 0 0 0 0 0 0 0 0 0 0 0 0 0 0 0 0 0 0
1994 2 Volkhov 3.5 W 10 0.86257 0 2 3 13 0 0 1 0 0 0 0 0 0 0 0 0 0 0 0 0 0 0 0 0
1994 2 Volkhov 4.2 W 10 0.88601 0 0 0 4 0 0 0 1 2 0 0 0 0 0 0 0 0 0 0 0 0 0 0 0
1994 2 Volkhov 8.5 N 10 1.41540 0 7 2 58 10 0 3 10 0 0 0 0 0 0 0 0 0 0 0 0 0 0 0 0
1994 2 Volkhov 4.3 S 10 0.76755 0 3 0 0 0 0 0 15 0 0 0 0 0 0 0 0 0 0 0 0 0 0 0 0
1994 2 Volkhov 11.6 N 10 1.35123 0 0 2 22 26 0 6 2 0 0 0 0 0 0 0 0 0 0 0 0 0 0 0 0
1994 2 Volkhov 9.4 S 10 0.68610 0 2 2 19 0 0 0 0 1 0 0 0 0 0 0 0 0 0 0 0 0 0 0 0
1994 2 Volkhov 13.9 N 10 1.08592 0 3 0 51 100 0 1 1 0 0 0 0 0 0 0 0 0 0 0 0 0 0 0 0
1994 2 Volkhov 13.0 S 10 1.65603 0 0 0 21 89 0 4 1 0 0 0 0 0 0 0 0 0 0 0 0 0 0 0 0
1994 2 Volkhov 15.9 N 10 2.67655 0 10 2 48 97 0 9 0 0 0 0 0 0 0 0 0 0 0 0 0 0 0 0 0
1994 2 Volkhov 15.3 S 10 1.60667 0 8 0 71 110 0 7 0 0 1 0 0 0 0 0 0 0 0 0 0 0 0 0 0
1995 1 Syasstro 0.3 N 10 0.97856 134 8 2 0 0 0 0 0 0 0 0 0 0 0 0 0 0 0 0 0 0 0 0 0
1995 1 Syasstro 2.7 W 10 1.48327 475 1 0 0 0 0 0 0 0 0 0 0 0 0 0 0 0 0 0 0 0 0 0 0
1995 1 Syasstro 3.2 N 10 2.45936 52 6 2 0 0 0 0 0 0 0 0 0 0 0 0 0 0 0 0 0 0 0 0 0
1995 1 Syasstro 3.5 W 10 1.07481 388 0 0 0 0 0 0 0 0 0 0 0 0 0 0 0 0 0 0 0 0 0 0 0
1995 1 Syasstro 5.0 N 10 1.32902 264 3 0 0 0 0 0 0 0 0 0 0 0 0 0 0 0 0 0 0 0 0 0 0
1995 1 Syasstro 5.2 W 10 1.24881 402 2 0 0 0 0 0 0 0 0 0 0 0 0 0 0 0 0 0 0 0 0 0 0
1995 1 Syasstro 7.6 W 10 1.85347 104 3 0 1 0 0 0 0 0 0 0 0 0 0 0 0 0 0 0 0 0 0 0 0
1995 1 Syasstro 8.6 N 10 0.98350 252 1 5 1 0 0 0 0 0 0 0 0 0 0 0 0 0 0 0 0 0 0 0 0
1995 1 Syasstro 9.6 W 10 1.56348 195 2 0 5 0 0 0 0 0 0 0 0 0 0 0 0 0 0 0 0 0 0 0 0
1995 1 Syasstro 11.7 W 6 0.74780 59 3 0 2 0 0 0 0 0 0 0 0 0 0 0 0 0 0 0 0 0 0 0 0
1995 1 Syasstro 14.2 N 10 1.08469 179 4 2 0 0 0 0 0 0 0 0 0 0 0 0 0 0 0 0 0 0 0 0 0
1995 1 Volkhov 0.4 W 10 1.63011 46 11 11 1 0 0 0 0 0 0 0 0 0 0 0 0 0 0 0 0 0 0 0 0
1995 1 Volkhov 1.4 W 10 1.18587 99 1 1 0 0 0 0 0 0 0 0 0 0 0 0 0 0 0 0 0 0 0 0 0
1995 1 Volkhov 1.8 W 10 2.43345 159 5 0 0 0 0 0 0 0 0 0 0 0 0 0 0 0 0 0 0 0 0 0 0
1995 1 Volkhov 2.6 W 10 2.65187 23 5 5 2 0 0 0 0 0 0 0 0 0 0 0 0 0 0 0 0 0 0 0 0
1995 1 Volkhov 1.9 S 10 1.61407 34 7 0 0 0 0 0 0 0 0 0 0 0 0 0 0 0 0 0 0 0 0 0 0
1995 1 Volkhov 3.5 W 10 1.48327 12 14 0 0 0 0 0 0 0 0 0 0 0 0 0 0 0 0 0 0 0 0 0 0
1995 1 Volkhov 4.2 W 10 1.73624 92 18 1 0 0 0 0 0 2 0 0 0 0 0 0 0 0 0 0 0 0 0 0 0
1995 1 Volkhov 8.5 N 10 2.49885 201 6 0 3 0 0 0 0 0 0 0 0 0 0 0 0 0 0 0 0 0 0 0 0
1995 1 Volkhov 4.3 S 10 1.56718 226 14 0 0 0 0 0 0 0 0 0 0 0 0 0 0 0 0 0 0 0 0 0 0
1995 1 Volkhov 11.6 N 10 1.85594 54 14 1 0 0 0 0 0 0 0 0 0 0 0 0 0 0 0 0 0 0 0 0 0
1995 1 Volkhov 9.4 S 10 1.53016 130 6 0 2 0 0 0 0 0 0 0 0 0 0 0 0 0 0 0 0 0 0 0 0
1995 1 Volkhov 13.0 S 10 2.33720 302 2 2 4 0 0 0 0 0 0 0 0 0 0 0 0 0 0 0 0 0 0 0 0
1995 1 Volkhov 15.9 N 10 2.79007 192 18 0 12 0 0 0 0 0 0 3 0 0 0 0 0 0 0 0 0 0 0 0 0
1995 1 Volkhov 15.3 S 1 0.59726 44 1 0 1 0 0 0 0 0 0 0 0 0 0 0 0 0 0 0 0 0 0 0 0
1995 2 Syasstro 0.3 N 10 1.12294 0 5 21 1 4 0 0 0 0 0 0 0 0 0 0 0 0 0 0 0 0 0 0 0
1995 2 Syasstro 2.7 W 10 0.92180 0 0 8 3 0 0 2 0 0 0 0 0 0 0 0 0 0 0 0 0 0 0 0 0
1995 2 Syasstro 3.2 N 10 1.92751 0 3 73 2 0 0 0 0 0 0 0 0 0 0 0 0 2 0 0 0 0 0 0 0
1995 2 Syasstro 3.5 W 10 0.99584 0 2 18 5 47 0 0 0 0 0 0 0 0 0 0 0 0 0 0 0 0 0 0 0
1995 2 Syasstro 5.0 N 10 0.97980 0 2 4 3 3 0 32 0 0 0 0 0 0 0 0 0 0 0 0 0 0 0 0 0
1995 2 Syasstro 5.2 W 10 0.92550 0 4 23 0 9 0 0 0 0 0 0 0 0 0 0 0 0 0 0 0 0 0 0 0
1995 2 Syasstro 7.6 W 10 1.05384 0 1 37 9 6 0 0 0 0 0 0 0 0 0 0 1 0 0 0 0 0 0 0 0
1995 2 Syasstro 8.6 N 10 1.29076 0 3 26 2 1 0 13 0 0 0 0 0 0 0 0 0 0 0 0 0 0 0 0 0
1995 2 Syasstro 9.6 W 10 1.64245 0 6 69 8 5 0 3 0 0 0 0 0 0 0 0 0 1 0 0 0 0 0 0 0
1995 2 Syasstro 11.7 W 10 1.13528 0 8 9 2 0 0 2 0 0 1 0 1 0 0 0 0 0 0 0 0 0 0 0 0
1995 2 Syasstro 12.8 W 10 1.65356 0 9 78 14 0 0 13 0 0 0 0 1 0 0 0 0 1 0 0 0 0 0 0 0
1995 2 Syasstro 14.2 N 10 0.97609 0 1 49 1 1 0 2 0 0 0 0 0 0 0 0 0 0 0 0 0 0 0 0 0
1995 2 Volkhov 0.4 W 10 0.93290 0 6 112 3 0 0 0 0 0 0 0 0 0 0 0 0 0 0 0 0 0 0 0 0
1995 2 Volkhov 1.4 W 10 1.90159 0 0 20 4 1 0 10 0 0 0 0 0 0 0 0 33 0 0 0 0 0 0 0 0
1995 2 Volkhov 1.8 W 10 1.71896 0 2 50 2 11 0 2 0 0 0 0 0 0 0 0 0 0 0 0 0 0 0 0 0
1995 2 Volkhov 2.6 W 10 1.19204 0 5 36 7 0 0 0 0 0 0 0 1 0 0 0 0 0 0 0 0 0 0 0 0
1995 2 Volkhov 1.9 S 10 1.20562 0 8 19 8 0 0 2 0 0 0 0 0 0 0 0 0 0 0 0 0 0 0 0 0
1995 2 Volkhov 3.5 W 10 0.73670 0 4 25 3 0 0 0 0 0 0 0 0 0 0 0 0 0 0 0 0 0 0 0 0
1995 2 Volkhov 4.2 W 10 1.07728 0 3 21 4 0 0 1 0 0 0 0 0 0 0 0 0 0 0 0 0 0 0 0 0
1995 2 Volkhov 8.5 N 10 0.64291 0 1 13 1 0 0 9 0 0 0 0 0 0 0 0 0 0 0 0 0 0 0 0 0
1995 2 Volkhov 4.3 S 10 1.00571 0 8 16 5 0 0 5 0 0 0 0 0 0 0 0 0 0 0 0 0 0 0 0 0
1995 2 Volkhov 11.6 N 10 1.29940 0 6 55 0 9 0 3 0 0 0 0 0 0 0 0 0 0 0 0 0 0 0 0 0
1995 2 Volkhov 9.4 S 10 0.87984 0 4 8 3 0 0 1 0 0 1 0 0 0 0 0 0 0 0 0 0 0 0 0 0
1995 2 Volkhov 13.9 N 10 1.11060 21 4 62 1 2 0 4 0 0 1 0 0 0 0 0 0 0 0 0 0 0 0 0 0
1995 2 Volkhov 13.0 S 10 2.12001 0 4 19 5 12 0 9 0 0 0 0 0 0 0 0 0 0 0 0 0 0 0 0 0
1995 2 Volkhov 15.9 N 10 3.84638 0 14 6 4 17 0 8 0 0 0 0 0 0 0 0 0 0 0 0 0 0 0 0 0
1995 2 Volkhov 15.3 S 10 1.43144 0 8 5 11 0 11 26 0 0 0 0 0 0 0 0 0 0 0 0 0 0 0 0 0
1996 1 Syasstro 0.3 N 10 0.68734 41 0 0 1 0 0 0 0 56 0 0 0 0 0 0 0 0 0 0 0 0 0 0 0
1996 1 Syasstro 2.7 W 10 0.53062 183 0 0 0 0 0 0 0 15 0 0 0 0 0 0 0 0 0 0 0 0 0 0 0

1996 1 Syasstro 3.2 N 10 0.53185 14 0 0 0 0 0 0 0 8 0 0 0 0 0 0 0 0 0 0 0 0 0 0 0 0
1996 1 Syasstro 3.5 W 10 0.59972 74 0 0 0 0 0 0 0 4 0 0 0 0 0 0 0 0 0 0 0 0 0 0 0 0
1996 1 Syasstro 5.0 N 10 1.01682 236 3 0 1 1 1 0 0 15 0 0 0 0 0 0 0 0 0 0 0 0 0 0 0 0
1996 1 Syasstro 5.2 W 10 0.74163 127 7 0 1 13 0 0 0 58 0 0 0 0 0 0 0 0 0 0 0 0 0 0 0 0
1996 1 Syasstro 7.6 W 10 1.12417 84 1 0 0 0 0 0 0 31 0 0 0 0 0 0 0 0 0 0 0 0 0 0 0 0
1996 1 Syasstro 8.6 N 10 0.73793 118 0 0 0 1 0 0 0 71 0 0 0 0 0 0 0 0 0 0 0 0 0 0 0 0
1996 1 Syasstro 9.6 W 10 0.87861 105 4 0 0 0 0 0 0 10 0 0 0 0 0 0 0 0 0 0 0 0 0 0 0 0
1996 1 Syasstro 11.7 W 10 1.47463 107 3 0 2 0 0 0 0 40 0 0 0 0 2 0 0 0 0 0 0 0 0 0 0 0
1996 1 Syasstro 12.8 W 10 0.77619 127 0 0 1 0 0 0 0 2 0 0 0 0 0 0 0 0 0 0 0 0 0 0 0 0
1996 1 Syasstro 14.2 N 10 0.86133 231 2 0 0 0 0 0 0 18 0 0 0 0 0 0 0 0 0 0 0 0 0 0 0 0
1996 1 Volkhov 0.4 W 10 1.00448 5 1 0 0 0 0 0 0 68 0 0 1 0 0 0 0 0 0 0 0 0 0 0 0 0
1996 1 Volkhov 1.4 W 10 1.09703 34 2 0 6 0 0 0 0 48 0 0 0 0 0 0 0 0 0 0 0 0 0 0 0 0
1996 1 Volkhov 1.8 W 10 1.64122 56 4 0 0 7 0 0 0 222 0 0 0 0 0 0 0 0 0 0 0 0 0 0 0 0
1996 1 Volkhov 2.6 W 10 0.79593 19 3 0 1 0 0 0 0 108 0 0 0 0 0 0 0 0 0 0 0 0 0 0 0 0
1996 1 Volkhov 1.9 S 10 0.89465 2 7 0 0 2 0 0 0 18 0 0 0 0 0 0 0 0 0 0 0 0 0 0 0 0
1996 1 Volkhov 3.5 W 10 0.71078 1 4 0 0 0 0 0 0 78 0 0 0 0 0 0 0 0 0 0 0 0 0 0 0 0
1996 1 Volkhov 4.2 W 10 0.63304 4 13 0 0 2 0 0 0 63 0 0 0 0 0 0 0 0 0 0 0 0 0 0 0 0
1996 1 Volkhov 8.5 N 10 0.77865 180 5 0 1 11 0 0 0 15 0 0 0 0 0 0 0 0 0 0 0 0 0 0 0 0
1996 1 Volkhov 4.3 S 10 0.94895 178 1 0 0 0 0 0 0 110 0 0 0 0 0 0 0 0 0 0 0 0 0 0 0 0
1996 1 Volkhov 11.6 N 10 1.16983 30 5 0 0 0 0 0 0 0 0 0 0 0 0 0 0 0 0 0 0 0 0 0 0 0
1996 1 Volkhov 9.4 S 10 0.62687 197 0 0 0 14 0 0 0 70 0 0 1 0 0 0 0 0 0 0 0 0 0 0 0 0
1996 1 Volkhov 13.9 N 10 0.91810 92 2 0 0 4 0 0 0 48 0 0 0 0 0 0 0 0 0 0 0 0 0 0 0 0
1996 1 Volkhov 13.0 S 10 2.57289 118 2 0 0 43 0 0 0 64 0 0 1 0 0 0 0 0 0 0 0 0 0 0 0 0
1996 1 Volkhov 15.9 N 10 1.50301 169 0 0 0 99 0 0 0 5 0 0 0 1 0 0 0 0 0 0 0 0 0 0 0 0
1996 1 Volkhov 15.3 S 10 2.25822 82 5 0 0 9 0 0 0 19 0 0 0 0 0 0 0 0 0 0 0 0 0 0 0 0
1996 2 Syasstro 0.3 N 10 1.83126 0 1 0 0 0 0 0 0 0 0 0 0 0 0 0 0 0 0 0 0 0 0 0 0 0
1996 2 Syasstro 2.7 W 10 2.47911 21 2 0 1 0 0 0 0 0 0 0 0 0 0 0 0 0 0 0 0 0 0 0 0 0
1996 2 Syasstro 3.2 N 10 3.44533 0 2 3 0 1 0 2 0 0 0 0 0 0 0 0 0 0 0 0 0 0 0 0 0
1996 2 Syasstro 3.5 W 10 3.11215 0 1 1 0 0 0 0 0 0 0 0 0 0 0 0 0 0 0 0 0 0 0 0 0
1996 2 Syasstro 5.0 N 10 2.63336 0 0 0 1 0 0 1 0 0 0 0 0 0 0 0 0 0 0 0 0 0 0 0 0
1996 2 Syasstro 5.2 W 10 1.89913 1 1 0 0 0 0 7 0 0 0 0 0 0 0 0 0 0 0 0 0 0 0 0 0
1996 2 Syasstro 7.6 W 10 2.12865 0 1 1 0 0 0 4 0 0 0 0 0 0 0 0 0 0 0 0 0 0 0 0 0
1996 2 Syasstro 8.6 N 10 2.43838 2 0 1 0 0 0 4 0 0 0 0 0 0 0 0 0 0 0 0 0 0 0 0 0
1996 2 Syasstro 9.6 W 10 2.12865 0 1 1 0 0 0 4 0 0 0 0 0 0 0 0 0 0 0 0 0 0 0 0 0
1996 2 Syasstro 11.7 W 10 2.66297 4 4 1 0 6 0 5 0 0 0 0 0 0 0 0 0 0 0 0 0 0 0 0 0
1996 2 Syasstro 12.8 W 10 2.96407 0 5 0 1 0 0 3 0 0 0 0 0 0 0 0 0 0 0 0 0 0 0 0 0
1996 2 Syasstro 14.2 N 10 1.77079 0 4 0 0 0 0 2 0 0 0 0 0 0 0 0 0 0 0 0 0 0 0 0 0
1996 2 Volkhov 0.4 W 10 1.75598 0 1 2 1 5 0 0 0 0 0 0 0 0 0 0 0 0 0 0 0 0 0 0 0
1996 2 Volkhov 1.4 W 10 1.81028 0 1 4 0 3 0 12 0 0 0 0 0 0 0 0 0 0 0 0 0 0 0 0 0
1996 2 Volkhov 1.8 W 10 2.23848 0 1 0 0 9 0 3 0 0 0 0 0 0 0 0 0 0 0 0 0 0 0 0 0
1996 2 Volkhov 2.6 W 10 1.67330 1 1 0 0 10 0 1 0 0 0 0 0 0 0 0 0 0 0 0 0 0 0 0 0
1996 2 Volkhov 1.9 S 10 1.82509 0 0 1 0 0 0 0 0 0 0 0 0 0 0 0 0 0 0 0 0 0 0 0 0
1996 2 Volkhov 3.5 W 10 2.06325 1 2 0 2 0 0 2 0 0 0 0 0 0 0 0 0 0 0 0 0 0 0 0 0
1996 2 Volkhov 4.2 W 10 2.28043 0 5 1 0 3 0 6 0 0 0 0 0 0 0 0 0 0 0 0 0 0 0 0 0
1996 2 Volkhov 8.5 N 10 2.62348 0 6 0 0 0 0 8 0 0 0 0 0 0 0 0 0 0 0 0 0 0 0 0 0
1996 2 Volkhov 4.3 S 10 2.58646 0 5 1 0 0 0 2 0 0 0 0 0 0 0 0 0 0 0 0 0 0 0 0 0
1996 2 Volkhov 11.6 N 10 2.10644 0 3 0 1 0 0 13 0 0 0 0 0 0 0 0 0 0 0 0 0 0 0 0 0
1996 2 Volkhov 9.4 S 10 2.34337 0 3 1 0 0 0 4 0 0 0 0 0 0 0 0 0 0 0 0 0 0 0 0 0
1996 2 Volkhov 13.9 N 10 2.42358 1 5 2 0 4 0 0 0 0 0 0 0 0 0 0 0 0 0 0 0 0 0 0 0
1996 2 Volkhov 13.0 S 10 2.81229 0 3 3 0 2 0 1 0 0 0 0 0 0 0 0 0 0 0 0 0 0 0 0 0
1996 2 Volkhov 15.9 N 10 3.41695 4 5 0 5 8 0 20 0 0 0 0 0 0 0 0 0 0 0 0 0 0 0 0 0
1996 2 Volkhov 15.3 S 10 2.84067 0 6 1 0 16 0 8 0 0 0 0 0 0 0 0 0 0 0 0 0 0 0 0 0
1997 1 Syasstro 0.3 N 10 0.95141 46 3 0 0 9 0 0 0 16 0 0 0 0 0 0 0 0 0 0 0 0 0 0 0 0
1997 1 Syasstro 2.7 W 10 0.85640 158 2 0 0 0 0 0 0 16 0 0 0 0 0 0 0 0 0 0 0 0 0 0 0 0
1997 1 Syasstro 3.2 N 10 1.41540 26 0 0 0 0 0 0 0 7 0 0 0 0 0 0 0 0 0 0 0 0 0 0 0 0
1997 1 Syasstro 3.5 W 10 1.20192 83 1 0 0 0 0 0 0 0 0 0 0 0 0 0 0 0 0 0 0 0 0 0 0 0
1997 1 Syasstro 5.0 N 10 1.39689 30 1 0 0 0 0 0 0 0 0 0 0 0 0 0 0 0 0 0 0 0 0 0 0 0
1997 1 Syasstro 5.2 W 10 1.73994 62 6 0 0 0 0 0 0 41 0 0 0 0 0 0 0 0 0 0 0 0 0 0 0 0
1997 1 Syasstro 7.6 W 10 1.38455 58 1 0 0 2 0 0 0 26 0 0 0 0 0 0 0 0 0 0 0 0 0 0 0 0
1997 1 Syasstro 8.6 N 10 0.80704 78 2 0 0 0 0 0 0 49 0 0 0 0 0 0 0 0 0 0 0 0 0 0 0 0
1997 1 Syasstro 9.6 W 10 1.03162 56 0 0 0 0 0 0 0 53 1 0 0 0 1 0 0 0 0 0 0 0 0 0 0 0
1997 1 Syasstro 11.7 W 14 2.23724 196 2 0 3 0 0 0 0 55 0 0 0 0 0 0 0 0 0 0 0 0 0 0 0 0
1997 1 Syasstro 12.8 W 20 2.45566 281 6 0 1 0 0 0 0 77 0 0 0 0 0 0 0 0 0 0 0 0 0 0 0 0
1997 1 Syasstro 14.2 N 10 1.28336 90 0 0 0 0 0 0 0 56 0 0 0 0 0 0 0 0 0 0 0 0 0 0 0 0
1997 1 Volkhov 0.4 W 10 1.16736 26 2 0 0 0 0 0 0 32 0 0 1 0 0 0 0 0 0 0 0 0 0 0 0 0
1997 1 Volkhov 1.4 W 10 1.68441 35 1 0 0 2 0 0 0 34 0 0 0 0 0 0 0 0 0 0 0 0 0 0 0 0
1997 1 Volkhov 1.8 W 10 1.34136 37 0 0 0 2 0 0 0 42 0 0 0 0 0 0 0 0 0 0 0 0 0 0 0 0
1997 1 Volkhov 2.6 W 10 1.01311 5 6 0 0 0 0 0 0 38 0 0 0 0 0 0 0 0 0 0 0 0 0 0 0 0
1997 1 Volkhov 1.9 S 10 0.88848 11 2 0 0 1 0 0 0 15 0 0 0 0 0 0 0 0 0 0 0 0 0 0 0 0
1997 1 Volkhov 3.5 W 10 1.11430 4 1 0 0 0 0 0 0 49 0 0 0 0 0 0 0 0 0 0 0 0 0 0 0 0
1997 1 Volkhov 4.2 W 10 0.87491 30 4 0 0 0 0 0 0 22 0 0 0 0 0 0 0 0 0 0 0 0 0 0 0 0
1997 1 Volkhov 8.5 N 10 3.46137 441 2 0 0 0 0 0 0 76 0 0 0 0 0 0 0 0 0 0 0 0 0 0 0 0
1997 1 Volkhov 4.3 S 10 0.83789 99 0 0 0 0 0 0 0 49 0 0 0 0 0 0 0 0 0 0 0 0 0 0 0 0
1997 1 Volkhov 11.6 N 10 1.30804 31 2 0 0 0 0 0 0 0 0 0 0 0 0 0 0 0 0 0 0 0 0 0 0 0
1997 1 Volkhov 9.4 S 10 0.65772 73 2 0 0 0 0 0 0 38 0 0 0 0 0 0 0 0 0 0 0 0 0 0 0 0
1997 1 Volkhov 13.9 N 10 1.48574 19 0 0 0 3 1 0 0 53 0 0 0 0 0 0 0 0 0 0 0 0 0 0 0 0
1997 1 Volkhov 13.0 S 10 3.44656 36 1 0 0 0 0 0 0 37 0 0 0 0 0 0 0 0 0 0 0 0 0 0 0 0
1997 1 Volkhov 15.9 N 10 2.93322 57 9 0 0 58 0 0 0 55 0 3 0 0 0 0 0 0 0 0 0 0 0 0 0 0
1997 1 Volkhov 15.3 S 19 2.95543 131 11 1 0 3 0 0 0 43 0 0 0 0 0 0 0 0 0 0 0 0 0 0 0 0
1997 2 Syasstro 0.3 N 10 1.94108 5 0 0 3 0 0 2 1 0 0 0 0 0 0 0 0 1 39 0 0 0 0 0 0
1997 2 Syasstro 2.7 W 10 2.33473 0 3 0 10 0 0 2 0 0 0 0 0 0 0 0 0 0 14 0 0 0 0 0 0
1997 2 Syasstro 3.2 N 10 1.52152 1 5 2 10 0 0 0 0 0 0 0 0 0 0 0 0 0 0 0 0 0 0 0 0
1997 2 Syasstro 3.5 W 10 1.78190 0 4 0 33 0 0 31 1 0 0 0 0 0 0 0 0 0 32 0 0 0 0 0 0
1997 2 Syasstro 5.0 N 10 2.22614 6 6 0 9 0 0 15 0 0 0 0 0 0 0 0 0 0 0 0 0 0 0 0 0
1997 2 Syasstro 5.2 W 10 3.25899 1 7 0 7 5 0 7 5 0 0 0 0 0 0 0 0 0 28 0 0 0 0 0 0
1997 2 Syasstro 7.6 W 10 1.46229 2 5 9 9 1 0 1 1 0 0 0 0 0 0 0 0 0 11 0 0 0 0 1 0
1997 2 Syasstro 8.6 N 10 1.45735 0 7 1 11 0 0 0 0 0 0 0 0 0 0 0 0 1 2 0 0 0 0 0 0
1997 2 Syasstro 9.6 W 10 2.41494 1 10 2 16 1 0 0 0 0 0 26 0 0 0 0 0 0 30 0 0 0 0 0 0

```
1997 2 Syasstro 11.7 W 10 2.76293 0 3 6 23 0 0 0 0 0 0 0 0 0 0 0 0 0 0 3 0 0 0 0 0 0
1997 2 Syasstro 12.8 W 10 2.65187 1 19 10 12 0 0 2 0 0 0 0 0 0 0 0 0 0 0 0 0 0 0 0 0 0 0
1997 2 Syasstro 14.2 N 10 3.43546 0 5 2 5 0 0 0 0 0 0 0 0 0 0 0 0 1 0 3 0 0 0 0 0 0
1997 2 Volkhov 0.4 W 10 1.56718 2 6 1 21 0 0 1 0 0 0 15 0 0 0 0 0 0 0 0 0 0 0 0 0 0
1997 2 Volkhov 1.4 W 10 1.56841 0 3 0 32 11 0 2 0 0 0 1 0 0 0 0 0 0 0 0 0 0 0 0 0 0
1997 2 Volkhov 1.8 W 10 1.45242 0 0 1 39 9 0 1 0 0 0 6 0 0 0 0 0 0 0 1 0 0 0 0 0 0
1997 2 Volkhov 2.6 W 10 3.76987 0 4 0 33 0 0 4 0 0 0 2 0 0 0 0 0 0 0 0 0 0 0 0 0 0
1997 2 Volkhov 1.9 S 10 1.99661 3 0 0 12 0 0 1 0 0 0 0 0 0 0 0 0 0 0 0 0 0 0 0 0 0
1997 2 Volkhov 3.5 W 10 1.58075 0 2 0 8 0 0 1 0 0 0 0 0 0 0 0 0 0 0 0 0 0 0 0 0
1997 2 Volkhov 4.2 W 10 1.86334 3 2 0 24 0 0 6 0 0 0 6 0 0 0 0 0 0 0 0 0 0 0 0 0 0
1997 2 Volkhov 8.5 N 10 2.94186 11 12 0 4 0 0 9 0 0 0 0 0 0 0 0 0 0 0 0 0 0 0 0 0 0
1997 2 Volkhov 4.3 S 10 2.77650 13 4 0 12 0 0 1 0 0 0 2 0 0 0 0 0 0 0 0 0 0 0 0 0 0
1997 2 Volkhov 11.6 N 10 1.79547 4 19 1 19 0 0 8 0 0 0 0 0 0 0 0 0 0 0 0 0 0 0 0 0 0
1997 2 Volkhov 9.4 S 10 2.86165 1 5 1 42 2 0 21 0 0 0 211 0 0 0 0 0 2 0 0 0 0 0 0 0
1997 2 Volkhov 13.9 N 10 2.84560 0 7 10 11 2 0 5 0 0 0 0 0 0 0 0 1 0 0 0 0 0 0 0
1997 2 Volkhov 13.0 S 10 1.90900 0 9 1 39 1 0 1 0 0 0 5 0 0 0 0 0 0 0 0 0 0 0 0 0 0
1997 2 Volkhov 15.9 N 10 2.25575 0 12 0 68 6 0 14 0 0 0 0 0 0 0 0 0 0 0 0 0 0 0 0 0 0
1997 2 Volkhov 15.3 S 10 1.73377 0 4 0 35 20 0 0 0 0 0 2 0 0 0 0 0 0 0 0 0 0 0 0 12
1998 1 Syasstro 0.3 N 10 1.42404 19 4 0 0 0 1 0 0 5 0 0 0 0 0 0 0 0 0 0 0 0 0 0 0 0
1998 1 Syasstro 2.7 W 10 1.12911 106 4 0 0 0 0 0 0 2 0 0 0 0 0 0 0 0 0 0 0 0 0 0 0 0
1998 1 Syasstro 3.2 N 10 1.85470 73 0 0 0 0 0 0 0 2 0 0 0 0 0 0 0 0 0 0 0 0 0 0 0 0
1998 1 Syasstro 3.5 W 10 1.68441 45 1 0 0 3 0 0 0 4 0 0 0 0 0 0 0 0 0 0 0 0 0 0 0 0
1998 1 Syasstro 5.0 N 10 1.50054 118 1 0 0 0 0 0 0 0 0 0 0 0 0 1 0 0 0 0 0 0 0 0 0
1998 1 Syasstro 5.2 W 10 1.35617 37 3 0 0 0 0 0 0 20 0 0 0 0 0 0 0 0 0 0 0 0 0 0 0 0
1998 1 Syasstro 7.6 W 10 1.29447 36 2 0 0 0 0 0 0 8 0 0 0 0 0 0 0 0 0 0 0 0 0 0 0 0
1998 1 Syasstro 8.6 N 10 0.70461 12 0 0 0 0 0 0 0 0 0 0 0 0 0 0 0 0 0 0 0 0 0 0 0 0
1998 1 Syasstro 9.6 W 10 1.77943 54 0 0 0 0 0 0 0 24 0 0 0 0 0 0 0 0 0 0 0 0 0 0 0 0
1998 1 Syasstro 11.7 W 10 1.55237 100 5 0 0 1 1 0 0 50 0 0 0 0 0 0 0 0 0 0 0 0 0 0 0 0
1998 1 Syasstro 14.2 N 10 1.37714 4 5 0 0 0 0 0 0 0 0 0 0 0 0 0 0 0 0 0 0 0 0 0 0 0
1998 1 Volkhov 0.4 W 10 1.33025 33 0 0 1 0 0 0 0 8 0 0 0 0 0 0 1 0 0 0 0 0 0 0 0
1998 1 Volkhov 1.4 W 10 1.69058 62 1 0 0 0 0 0 0 2 0 0 0 0 0 0 0 0 0 0 0 0 0 0 0
1998 1 Volkhov 1.8 W 10 0.83048 108 0 0 0 1 0 0 0 15 0 0 0 0 0 0 2 0 0 0 0 0 0 0 0
1998 1 Volkhov 2.6 W 10 0.85023 5 7 0 1 0 0 0 0 16 0 0 0 0 0 0 0 0 0 0 0 0 0 0 0
1998 1 Volkhov 1.9 S 10 1.63628 26 1 0 0 0 0 0 0 6 0 0 0 0 0 0 0 0 0 0 0 0 0 0 0
1998 1 Volkhov 3.5 W 10 0.83912 14 2 0 1 0 0 0 0 9 0 0 0 0 0 0 0 0 0 0 0 0 0 0 0
1998 1 Volkhov 4.2 W 10 1.12541 5 2 0 0 0 0 0 0 3 0 0 0 0 0 0 0 0 0 0 0 0 0 0
1998 1 Volkhov 8.5 N 10 2.46923 619 3 0 2 6 0 0 0 21 0 0 0 0 0 0 0 0 0 0 0 0 0 0 0
1998 1 Volkhov 4.3 S 10 1.13528 80 7 0 0 0 0 0 0 1 0 0 0 0 0 0 0 0 0 0 0 0 0 0 0
1998 1 Volkhov 11.6 N 10 1.09949 35 5 0 0 0 0 0 0 0 0 0 1 0 0 0 0 0 0 0 0 0 0 0 0
1998 1 Volkhov 9.4 S 10 0.90699 138 0 0 0 0 0 0 0 6 0 0 0 0 0 0 0 0 0 0 0 0 0 0 0
1998 1 Volkhov 13.9 N 10 1.08592 38 1 0 0 0 0 0 0 0 0 0 0 0 0 1 0 0 0 0 0 0 0 0 0
1998 1 Volkhov 13.0 S 10 1.11060 41 0 0 0 2 0 0 0 8 0 0 0 0 0 0 0 0 0 0 0 0 0 0 0
1998 1 Volkhov 15.9 N 10 1.44501 35 9 0 0 8 0 0 0 5 0 1 0 0 0 0 0 0 0 0 0 0 0 0 0
1998 1 Volkhov 15.3 S 10 1.19204 36 4 0 0 1 0 0 0 10 0 0 0 0 0 0 0 0 0 0 0 0 0 0 0
1998 2 Syasstro 0.3 N 10 3.26270 0 37 5 19 3 0 10 0 0 0 56 0 0 0 0 0 0 0 0 0 0 0 0 0 0
1998 2 Syasstro 2.7 W 10 3.05045 37 4 0 7 0 0 0 0 0 0 20 0 0 0 0 0 0 0 0 0 0 0 0 0
1998 2 Syasstro 3.2 N 10 4.38564 0 0 1 24 32 0 0 1 0 0 0 0 0 0 0 0 0 0 0 0 0 0 0 0 0
1998 2 Syasstro 3.5 W 10 2.22490 0 17 0 3 0 0 2 0 0 0 54 0 0 0 0 0 0 0 0 0 0 0 0 0
1998 2 Syasstro 5.0 N 10 3.81306 5 31 3 25 0 0 13 2 0 0 30 0 0 0 0 0 0 0 0 0 0 0 0 0 0
1998 2 Syasstro 5.2 W 10 2.16320 0 23 1 5 0 0 2 4 0 0 60 1 0 0 0 0 0 0 0 0 0 0 0 0
1998 2 Syasstro 7.6 W 10 3.41201 1 19 3 16 6 0 6 2 0 0 22 0 0 0 5 0 0 0 0 0 0 0 0 0
1998 2 Syasstro 8.6 N 10 3.19729 0 45 0 6 0 0 0 1 0 0 100 0 0 0 0 0 0 0 0 0 0 0 0 0
1998 2 Syasstro 9.6 W 10 2.12865 0 21 2 3 0 0 0 0 0 0 8 0 0 0 0 0 0 0 0 0 0 0 0 0
1998 2 Syasstro 11.7 W 10 2.48528 1 20 2 11 0 0 0 0 0 0 17 0 0 0 0 0 0 0 0 0 0 0 0 0 0
1998 2 Syasstro 12.8 W 10 2.00895 0 23 10 8 0 0 2 0 0 0 14 0 0 0 0 0 0 0 0 0 0 0 0 0 0
1998 2 Syasstro 14.2 N 10 4.92736 0 47 0 4 0 0 0 0 0 0 0 0 0 0 0 2 0 0 0 0 0 0 0 0
1998 2 Volkhov 0.4 W 10 3.46014 0 1 0 13 7 0 15 0 0 0 40 1 0 0 0 0 0 0 0 0 0 0 0 0 0
1998 2 Volkhov 1.4 W 10 1.98674 0 4 0 4 0 0 11 0 0 0 0 0 0 0 0 0 0 0 0 0 0 0 0 0
1998 2 Volkhov 1.8 W 10 3.23678 0 4 0 8 2 0 0 1 0 0 0 0 0 0 0 0 0 0 0 0 0 0 0
1998 2 Volkhov 2.6 W 10 5.78746 1 19 0 9 0 0 3 0 0 0 0 0 0 0 0 0 0 0 0 0 0 0 0 0
1998 2 Volkhov 1.9 S 10 3.07513 1 4 0 5 0 0 1 0 0 0 3 0 0 0 0 0 0 0 0 0 0 0 0
1998 2 Volkhov 3.5 W 10 2.04967 0 1 0 13 0 0 13 0 0 0 0 0 0 0 0 0 0 0 0 0 0 0 0 0
1998 2 Volkhov 4.2 W 10 2.15333 0 6 0 5 0 0 1 0 0 0 0 0 0 0 0 0 0 0 0 0 0 0 0
1998 2 Volkhov 8.5 N 10 2.16690 0 8 0 1 0 0 3 0 0 0 0 1 0 0 0 0 0 0 0 0 0 0 0
1998 2 Volkhov 4.3 S 10 2.29647 0 2 1 10 0 0 0 0 0 0 2 0 0 0 0 0 0 0 0 0 0 0 0 0
1998 2 Volkhov 11.6 N 10 2.31869 0 5 0 4 0 0 7 1 0 0 0 0 0 0 0 0 0 0 0 0 0 0 0
1998 2 Volkhov 9.4 S 10 2.21750 0 0 2 4 0 0 1 0 0 0 70 0 0 0 0 0 0 0 0 0 0 0 0
1998 2 Volkhov 13.9 N 10 3.43299 0 15 0 5 0 0 2 0 0 0 0 0 0 0 0 0 0 0 0 0 0 0 0 0
1998 2 Volkhov 13.0 S 10 2.11508 0 1 0 10 0 0 1 0 0 0 0 0 0 0 0 0 0 0 0 0 0 0 0 0
1998 2 Volkhov 15.9 N 10 3.11955 0 6 1 16 0 0 9 0 0 0 0 0 0 0 0 0 0 0 0 0 0 0 0 0
1998 2 Volkhov 15.3 S 10 2.76293 0 15 2 4 0 0 1 0 0 0 0 1 0 0 0 0 0 0 0 0 0 0 0 0
1999 1 Syasstro 0.3 N 10 1.06494 49 3 0 0 0 0 0 0 0 0 0 0 0 0 0 0 0 0 0 0 0 0 0 0 0
1999 1 Syasstro 2.7 W 10 1.06001 232 1 0 0 0 0 0 0 0 0 0 0 0 0 0 0 0 0 0 0 0 0 0 0 0
1999 1 Syasstro 3.2 N 10 1.38208 9 0 0 0 1 0 0 0 0 0 0 0 0 0 0 0 0 0 0 0 0 0 0 0
1999 1 Syasstro 3.5 W 10 0.92056 8 0 0 0 0 0 0 0 0 0 0 0 0 0 1 0 0 0 0 0 0 0 0 0
1999 1 Syasstro 5.0 N 10 1.27966 5 0 0 0 0 0 0 0 0 0 0 0 0 0 0 0 0 0 0 0 0 0 0 0
1999 1 Syasstro 5.2 W 10 1.26979 36 0 0 0 0 0 0 0 13 0 0 0 0 0 0 0 0 0 0 0 0 0 0 0 0
1999 1 Syasstro 7.6 W 10 1.55361 33 3 0 0 0 0 0 0 3 0 0 0 0 0 0 0 0 0 0 0 0 0 0 0
1999 1 Syasstro 8.6 N 10 1.49561 9 2 0 0 0 0 0 0 7 0 0 0 0 0 0 0 0 0 0 0 0 0 0 0
1999 1 Syasstro 9.6 W 10 1.47463 101 5 0 0 0 0 0 0 3 0 0 0 0 0 0 0 0 0 0 0 0 0 0 0 0
1999 1 Syasstro 11.7 W 10 0.78606 64 0 0 0 0 0 0 0 0 0 0 0 0 0 0 0 0 0 0 0 0 0 0 0 0
1999 1 Syasstro 12.8 W 10 1.14885 52 0 0 3 0 0 0 0 0 0 0 0 0 0 0 0 0 0 0 0 0 0 0 0 0
1999 1 Syasstro 14.2 N 10 1.32038 10 2 0 0 0 2 0 0 0 0 0 0 0 0 0 0 0 0 0 0 0 0 0 0 0
1999 1 Volkhov 0.4 W 10 1.96823 175 0 0 0 0 0 0 0 8 0 0 0 0 0 0 0 0 0 0 0 0 0 0 0
1999 1 Volkhov 1.4 W 10 1.69305 62 0 0 0 0 0 0 0 0 0 0 0 0 0 0 0 0 0 0 0 0 0 0 0
1999 1 Volkhov 1.8 W 10 1.94232 51 0 0 0 0 0 0 0 2 0 0 0 0 0 0 0 0 0 0 0 0 0 0 0
1999 1 Volkhov 2.6 W 10 1.32655 26 0 0 0 0 0 0 0 7 0 0 0 0 0 0 0 0 0 0 0 0 0 0 0
1999 1 Volkhov 1.9 S 10 1.61531 2 0 0 0 0 0 0 0 0 0 0 0 0 0 0 0 0 0 0 0 0 0 0 0
```

1999 1 Volkhov 3.5 W 10 0.95141 6 5 0 0 0 0 0 0 0 0 0 0 0 0 0 0 0 0 0 0 0 0 0 0
1999 1 Volkhov 4.2 W 10 1.15749 36 2 0 0 0 0 0 0 14 0 0 0 0 0 0 0 0 0 0 0 0 0 0 0
1999 1 Volkhov 8.5 N 10 1.06864 279 1 0 0 0 0 0 0 9 0 0 0 0 0 0 0 0 0 0 0 0 0 0 0
1999 1 Volkhov 4.3 S 10 1.42897 122 0 0 0 0 0 0 0 4 0 0 0 0 0 0 0 0 0 0 0 0 0 0 0
1999 1 Volkhov 11.6 N 10 1.46969 10 1 0 0 0 0 0 0 0 0 0 0 0 0 0 0 0 0 0 0 0 0 0 0
1999 1 Volkhov 9.4 S 10 1.58569 16 0 0 0 0 0 0 0 0 0 0 0 0 0 0 0 0 0 0 0 0 0 0 0
1999 1 Volkhov 13.9 N 10 1.34383 50 1 0 0 0 0 0 0 0 0 0 0 0 0 0 0 0 0 0 0 0 0 0 0
1999 1 Volkhov 13.0 S 10 1.61901 38 2 0 0 0 0 0 0 0 0 0 0 0 0 0 0 0 0 0 0 0 0 0 0
1999 1 Volkhov 15.9 N 10 1.38948 8 2 0 0 1 0 0 0 9 0 0 0 0 0 0 0 0 0 0 0 0 0 0 0
1999 1 Volkhov 15.3 S 10 1.71403 9 0 0 0 0 0 0 0 0 0 0 0 0 0 0 0 0 0 0 0 0 0 0 0
1999 2 Syasstro 0.3 N 10 1.04273 0 3 0 5 0 0 2 0 0 0 0 0 0 0 0 0 0 0 0 0 0 0 0 0
1999 2 Syasstro 2.7 W 10 1.17107 0 7 0 1 0 0 2 0 0 0 0 0 0 0 0 0 0 0 0 0 0 0 0 0
1999 2 Syasstro 3.2 N 10 1.13405 0 3 2 0 0 0 1 0 0 0 0 0 0 0 0 0 0 0 0 0 0 0 0 0
1999 2 Syasstro 3.5 W 10 1.16490 0 3 0 4 0 0 1 0 0 0 0 1 0 0 0 2 0 0 0 0 1 0 0 0
1999 2 Syasstro 5.0 N 10 0.99214 5 10 3 2 0 0 4 0 0 0 0 0 0 0 0 0 0 0 0 0 0 0 0 0
1999 2 Syasstro 5.2 W 10 1.15132 0 21 2 4 0 0 7 3 0 0 0 0 0 0 0 0 0 0 0 0 2 0 0 0
1999 2 Syasstro 7.6 W 10 1.74611 20 11 2 6 1 0 0 0 0 0 0 0 0 0 0 0 0 0 0 0 2 0 0 0
1999 2 Syasstro 8.6 N 10 1.19945 3 9 0 0 0 0 7 0 2 0 0 0 0 0 0 0 0 0 0 0 3 0 0 0
1999 2 Syasstro 9.6 W 10 1.44378 0 6 8 4 0 0 0 1 1 0 0 0 0 0 0 0 0 0 0 0 1 0 0 0
1999 2 Syasstro 11.7 W 10 1.48203 0 7 2 2 0 0 5 0 0 0 0 0 0 0 0 0 0 0 0 0 1 0 0 0
1999 2 Syasstro 12.8 W 10 1.20685 2 6 0 1 0 0 4 0 0 0 0 0 0 0 0 0 0 1 0 0 9 0 0 0
1999 2 Syasstro 14.2 N 10 1.57212 0 18 0 1 0 0 0 0 0 0 0 0 0 0 0 2 0 0 0 0 0 0 0 0
1999 2 Volkhov 0.4 W 10 1.30064 0 9 0 4 2 0 1 0 0 0 0 1 0 0 0 0 0 0 0 0 0 0 0 0
1999 2 Volkhov 1.4 W 10 1.27472 1 15 0 1 0 0 3 0 2 0 0 0 0 0 0 0 0 0 0 0 0 0 0 0
1999 2 Volkhov 1.8 W 10 1.09456 1 7 0 1 0 0 0 1 0 0 0 0 0 0 0 0 0 0 0 0 0 0 0 0
1999 2 Volkhov 2.6 W 10 1.18711 0 31 0 0 0 0 1 0 0 0 0 0 0 0 0 0 0 0 0 0 0 0 0 0
1999 2 Volkhov 1.9 S 10 1.06494 0 1 1 0 0 0 1 0 0 0 0 0 0 0 0 0 0 0 0 0 0 0 0 0
1999 2 Volkhov 3.5 W 10 1.17107 0 10 0 2 0 0 0 0 0 0 0 0 0 0 0 0 0 0 0 0 0 0 0 0
1999 2 Volkhov 4.2 W 10 1.11677 3 6 0 4 0 0 3 0 0 0 0 0 0 0 0 0 0 0 0 0 0 0 0 0
1999 2 Volkhov 8.5 N 10 1.30187 5 17 0 2 0 0 2 0 0 0 0 0 0 0 0 0 0 0 0 0 0 0 0 0
1999 2 Volkhov 4.3 S 10 1.18834 2 10 0 0 0 0 9 0 0 0 0 0 1 0 0 0 0 0 0 0 0 0 0 0
1999 2 Volkhov 11.6 N 10 0.90946 1 16 0 0 0 0 12 0 3 0 0 0 0 0 0 0 0 0 0 0 2 0 3 0
1999 2 Volkhov 9.4 S 10 1.34876 0 3 0 2 0 0 3 0 0 0 0 0 0 0 0 0 0 0 0 0 0 0 0 0
1999 2 Volkhov 13.9 N 10 0.76138 0 12 0 2 0 0 14 0 0 0 0 0 0 0 0 0 0 0 0 0 0 0 0 0
1999 2 Volkhov 13.0 S 10 1.06864 0 10 0 2 0 0 2 0 0 0 0 0 0 0 0 0 0 0 0 0 1 0 0 0
1999 2 Volkhov 15.9 N 10 1.28706 0 5 0 2 0 0 41 1 0 0 0 0 0 0 0 0 0 0 0 0 0 0 0 0
1999 2 Volkhov 15.3 S 10 0.72929 0 16 0 0 3 0 1 0 0 0 0 0 0 0 0 0 0 0 0 0 0 0 0 0
2000 1 Syasstro 0.3 N 10 0.99831 20 1 0 0 0 0 0 0 4 0 0 0 0 0 0 0 0 0 0 0 0 0 0 0
2000 1 Syasstro 2.7 W 10 1.20192 82 0 0 0 0 0 0 0 7 0 0 0 0 0 0 0 0 0 0 0 0 0 0 0
2000 1 Syasstro 3.2 N 10 1.28336 58 1 0 0 0 0 0 0 0 0 0 1 0 0 0 0 0 0 0 0 0 0 0 0
2000 1 Syasstro 3.5 W 10 1.39565 50 0 0 0 0 0 0 0 9 0 0 0 0 0 0 0 0 0 0 0 0 0 0 0
2000 1 Syasstro 5.0 N 10 1.64122 58 0 0 0 0 0 0 0 0 0 0 0 0 0 0 0 0 0 0 0 0 0 0 0
2000 1 Syasstro 5.2 W 10 0.96992 43 0 0 0 0 0 0 0 0 0 0 0 0 0 0 0 0 0 0 0 0 0 0 0
2000 1 Syasstro 7.6 W 10 1.68441 52 0 0 0 2 0 0 0 7 0 0 0 0 0 0 0 0 0 0 0 0 0 0 0
2000 1 Syasstro 8.6 N 10 1.53263 49 7 0 0 0 0 0 0 9 0 0 0 0 0 0 0 0 0 0 0 0 0 0 0
2000 1 Syasstro 9.6 W 10 1.39812 99 2 0 0 0 0 0 0 8 0 0 0 0 0 0 0 0 0 0 0 0 0 0 0
2000 1 Syasstro 11.7 W 10 1.42404 148 1 0 0 0 0 0 0 5 0 0 0 0 0 0 0 0 0 0 0 0 0 0 0
2000 1 Syasstro 12.8 W 10 1.65479 131 12 0 0 2 0 0 0 13 0 0 0 0 0 0 0 0 0 0 0 0 0 0 0
2000 1 Syasstro 14.2 N 10 2.08423 28 3 0 0 0 0 0 0 8 0 0 0 0 0 0 0 0 0 0 0 0 0 0 0
2000 1 Volkhov 0.4 W 10 1.49437 36 0 0 0 0 0 0 0 12 0 0 0 0 0 0 0 0 0 0 0 0 0 0 0
2000 1 Volkhov 1.4 W 10 1.49561 36 3 0 0 0 0 0 0 1 0 0 0 0 0 0 1 0 0 0 0 0 0 0 0
2000 1 Volkhov 1.8 W 10 1.20809 60 1 0 0 0 0 0 0 2 0 0 0 0 0 0 0 0 0 0 0 0 0 0 0
2000 1 Volkhov 2.6 W 10 0.97363 24 0 0 0 0 0 0 0 6 0 0 0 0 0 0 0 0 0 0 0 0 0 0 0
2000 1 Volkhov 1.9 S 10 1.58692 9 0 0 0 0 0 0 0 3 0 0 0 0 0 0 0 0 0 0 0 0 0 0 0
2000 1 Volkhov 3.5 W 10 1.46106 8 1 0 0 0 0 0 0 0 0 0 0 0 0 0 0 0 0 0 0 0 0 0 0
2000 1 Volkhov 4.2 W 10 1.63628 15 0 0 0 0 0 0 0 2 0 0 0 0 0 0 0 0 0 0 0 0 0 0 0
2000 1 Volkhov 8.5 N 10 2.12495 696 0 0 0 9 0 0 0 0 0 0 0 0 0 0 0 0 0 0 0 0 0 0 0
2000 1 Volkhov 4.3 S 10 1.10813 100 0 0 0 2 0 0 0 6 0 0 0 0 0 0 0 0 0 0 0 0 0 0 0
2000 1 Volkhov 11.6 N 10 2.00525 103 0 0 0 0 0 0 0 0 0 0 0 0 0 0 0 0 0 0 0 0 0 0 0
2000 1 Volkhov 9.4 S 10 1.34876 14 0 0 0 0 0 0 0 4 0 0 0 0 0 0 0 0 0 0 0 0 0 0 0
2000 1 Volkhov 13.9 N 10 1.72760 68 0 0 0 0 0 0 0 1 0 0 0 0 0 0 0 0 0 0 0 0 0 0 0
2000 1 Volkhov 13.0 S 10 1.68564 38 0 0 0 0 0 0 0 4 0 0 0 0 0 0 0 0 0 0 0 0 0 0 0
2000 1 Volkhov 15.9 N 10 1.44131 38 3 0 0 16 0 0 0 21 0 0 0 0 0 0 0 0 0 0 0 0 0 0 0
2000 1 Volkhov 15.3 S 10 1.62271 42 2 0 0 2 0 0 0 0 0 0 2 0 0 0 0 0 0 0 0 0 0 0 0
2000 2 Syasstro 0.3 N 10 2.53710 0 13 1 0 0 0 4 0 0 0 0 0 0 0 0 0 0 0 0 0 0 0 0 0
2000 2 Syasstro 2.7 W 10 3.02207 1 17 0 1 0 0 1 0 0 0 0 0 0 0 0 0 0 0 0 0 0 0 0 0
2000 2 Syasstro 3.2 N 10 2.57536 13 8 1 2 0 0 1 0 0 0 0 0 0 0 0 0 0 0 0 0 0 0 0 0
2000 2 Syasstro 3.5 W 10 2.72097 0 14 0 0 0 0 4 0 0 0 0 0 0 0 0 0 0 0 0 0 0 0 0 0
2000 2 Syasstro 5.0 N 10 4.73239 0 21 26 2 0 0 27 2 0 0 0 0 0 0 0 0 0 6 0 1 0 0 0 0
2000 2 Syasstro 5.2 W 10 1.83743 0 17 3 5 0 0 4 10 0 0 0 0 0 0 0 1 0 0 0 0 0 0 0 0
2000 2 Syasstro 7.6 W 10 3.88587 0 20 0 2 0 0 1 0 0 0 0 0 0 0 0 2 0 0 0 0 0 0 0 0
2000 2 Syasstro 8.6 N 10 1.92874 0 21 0 1 0 0 2 0 0 0 0 0 0 0 0 0 0 0 0 0 0 0 0 0
2000 2 Syasstro 9.6 W 10 2.87769 0 11 21 2 0 0 0 0 0 0 0 0 0 0 0 0 0 0 0 0 0 0 0 0
2000 2 Syasstro 11.7 W 10 1.90653 0 11 0 1 1 0 2 1 0 1 0 0 0 0 0 2 0 0 0 0 0 0 0 0
2000 2 Syasstro 12.8 W 10 4.26964 0 18 0 0 0 0 10 0 0 0 0 0 0 0 0 0 0 0 0 1 0 0 0 0
2000 2 Syasstro 14.2 N 10 2.88879 0 6 0 0 0 0 2 0 0 0 0 0 0 0 0 0 0 0 0 0 0 0 0 0
2000 2 Volkhov 0.4 W 10 2.16814 1 7 0 0 0 0 7 0 0 0 0 0 0 0 0 0 0 0 0 0 0 0 0 0
2000 2 Volkhov 1.4 W 10 2.68395 0 11 1 0 0 0 0 0 0 0 0 0 0 0 0 0 0 1 0 0 0 0 0 0
2000 2 Volkhov 1.8 W 10 4.10428 0 11 0 1 2 0 2 0 0 0 0 0 0 0 0 0 0 0 0 0 0 0 0 0
2000 2 Volkhov 2.6 W 10 4.34245 0 27 0 1 0 0 0 0 0 0 0 0 0 0 0 0 0 0 0 0 0 0 0 0
2000 2 Volkhov 1.9 S 10 1.93121 1 3 2 0 0 0 0 0 0 0 0 0 0 0 0 0 0 0 0 0 0 0 0 0
2000 2 Volkhov 3.5 W 10 2.16320 1 10 1 0 0 0 1 0 0 0 0 0 0 0 0 0 0 0 0 0 0 0 0 0
2000 2 Volkhov 4.2 W 10 3.25282 0 5 2 1 0 0 3 0 0 0 0 0 0 0 0 0 0 0 0 0 0 0 0 0
2000 2 Volkhov 8.5 N 10 2.22120 1 11 0 0 0 0 0 0 0 0 0 0 0 0 0 0 0 0 0 2 0 0 0 0
2000 2 Volkhov 4.3 S 10 2.88633 0 4 0 1 0 0 1 0 0 0 0 0 0 0 0 0 0 0 0 0 0 0 0 0
2000 2 Volkhov 11.6 N 10 1.46723 0 5 1 3 0 0 13 0 0 0 0 0 0 0 0 0 0 0 0 2 0 0 0 0
2000 2 Volkhov 9.4 S 10 3.45643 0 9 1 1 1 0 1 0 0 0 0 0 0 0 0 0 0 0 0 0 0 0 0 0
2000 2 Volkhov 13.9 N 10 3.98335 0 10 1 0 0 0 2 0 0 0 0 0 0 0 0 0 0 0 0 1 0 0 0 0

```
2000 2 Volkhov 13.0 S 10 1.96700 0 4 0 0 0 0 0 0 0 0 0 0 0 0 0 0 0 0 0 0 0 0 0 0
2000 2 Volkhov 15.9 N 10 1.93368 6 23 2 4 0 0 5 0 0 0 0 0 0 0 0 0 0 2 0 1 0 0 0 0
2000 2 Volkhov 15.3 S 10 2.51489 0 6 0 0 0 0 3 0 0 0 0 0 0 0 0 0 0 0 0 0 0 0 0 0
2001 1 Syasstro 0.3 N 10 1.41046 136 0 0 0 0 0 0 0 7 0 0 0 0 0 0 0 0 0 0 0 0 0 0 0
2001 1 Syasstro 2.7 W 10 1.35493 200 0 0 0 0 0 0 0 0 0 0 0 0 0 0 0 0 0 0 0 0 0 0 0
2001 1 Syasstro 3.2 N 10 1.52522 23 0 0 0 0 0 0 0 0 0 0 0 0 0 0 0 0 0 0 0 0 0 0 0
2001 1 Syasstro 3.5 W 10 1.06247 13 0 0 0 0 0 0 0 0 0 0 0 0 0 0 0 0 0 0 0 0 0 0 0
2001 1 Syasstro 5.0 N 10 1.72143 64 0 0 0 0 0 0 0 0 0 0 0 0 0 0 0 0 0 0 0 0 0 0 0
2001 1 Syasstro 5.2 W 10 1.76585 25 1 0 0 12 0 0 0 7 0 0 0 0 0 0 0 0 0 0 0 0 0 0 0
2001 1 Syasstro 7.6 W 10 1.92381 29 2 0 0 0 0 0 0 0 0 0 0 0 0 0 0 0 0 0 0 0 0 0 0
2001 1 Syasstro 8.6 N 10 1.58939 81 1 0 0 1 0 0 0 1 0 0 0 0 0 0 0 0 0 0 0 0 0 0 0
2001 1 Syasstro 9.6 W 10 1.50054 90 1 0 0 10 0 0 0 12 0 0 0 0 0 0 0 0 0 0 0 0 0 0 0
2001 1 Syasstro 11.7 W 10 1.24634 37 6 3 0 0 0 0 0 10 0 0 0 0 0 0 0 0 0 0 0 0 0 0 0
2001 1 Syasstro 12.8 W 10 1.66096 92 3 0 0 0 0 0 0 22 0 0 0 0 0 0 0 0 0 0 0 0 0 0 0
2001 1 Syasstro 14.2 N 10 1.45859 20 0 0 0 0 0 0 0 55 0 0 0 0 0 0 0 0 0 0 0 0 0 0 0
2001 1 Volkhov 0.4 W 10 1.54620 8 2 0 0 21 0 0 0 0 0 0 0 0 0 0 0 0 0 0 0 0 0 0 0
2001 1 Volkhov 1.4 W 10 1.16490 65 5 0 0 4 0 0 0 20 0 0 0 0 0 0 0 0 0 0 0 0 0 0 0
2001 1 Volkhov 1.8 W 10 1.52029 70 4 0 0 5 0 0 0 51 0 0 0 0 0 0 0 0 0 0 0 0 0 0 0
2001 1 Volkhov 2.6 W 10 1.53016 19 9 0 0 0 0 0 0 91 0 0 0 0 0 0 0 0 0 0 0 0 0 0 0
2001 1 Volkhov 1.9 S 10 1.74117 11 1 0 0 0 0 0 0 5 0 0 0 0 0 0 0 0 0 0 0 0 0 0 0
2001 1 Volkhov 3.5 W 10 1.30557 16 4 0 0 0 0 0 0 9 0 0 0 0 0 0 0 0 0 0 0 0 0 0 0
2001 1 Volkhov 4.2 W 10 1.17600 15 1 0 0 0 0 0 0 34 0 0 0 0 0 1 0 0 0 0 0 0 0 0
2001 1 Volkhov 8.5 N 10 0.94524 390 1 0 0 0 0 0 0 0 0 0 0 0 0 0 0 0 0 0 0 0 0 0 0
2001 1 Volkhov 4.3 S 10 1.21796 29 0 0 0 0 0 0 0 9 0 0 0 0 0 0 0 0 0 0 0 0 0 0 0
2001 1 Volkhov 11.6 N 10 1.26115 62 3 0 0 0 0 0 0 0 0 0 0 0 0 0 0 0 0 0 0 0 0 0 0
2001 1 Volkhov 9.4 S 10 1.30434 36 0 0 0 0 0 0 0 33 0 0 0 0 0 0 0 0 0 0 0 0 0 0 0
2001 1 Volkhov 13.9 N 10 2.06695 77 5 0 0 1 0 0 0 2 0 0 0 0 0 0 0 0 0 0 0 0 0 0 0
2001 1 Volkhov 13.0 S 10 1.47463 77 0 0 0 2 0 0 0 0 0 0 0 0 0 0 0 0 0 0 0 0 0 0 0
2001 1 Volkhov 15.9 N 10 1.89296 57 3 0 0 42 0 0 0 7 0 0 0 0 0 0 0 0 0 0 0 0 0 0 0
2001 1 Volkhov 15.3 S 10 2.42728 50 0 0 0 3 0 0 0 15 0 0 0 0 0 0 0 0 0 0 0 0 0 0 0
2001 2 Syasstro 0.3 N 10 2.24465 30 11 1 1 0 0 3 0 0 0 0 0 0 0 0 0 1 1 0 0 0 0 0 0
2001 2 Syasstro 2.7 W 10 2.20022 54 2 0 0 1 0 2 0 0 0 0 0 0 0 0 0 0 0 0 0 0 0 0 0
2001 2 Syasstro 3.2 N 10 1.52769 13 22 3 2 0 0 1 0 1 0 0 0 0 0 0 0 0 0 0 0 1 0 0 0
2001 2 Syasstro 3.5 W 10 1.35123 0 21 0 8 0 0 10 2 0 0 0 0 0 0 0 2 0 0 0 0 0 0 0 0
2001 2 Syasstro 5.0 N 10 1.87074 5 31 6 0 0 0 27 9 1 0 0 0 0 0 0 8 0 1 0 0 0 0 2 0
2001 2 Syasstro 5.2 W 10 1.64122 2 20 4 8 4 0 18 4 0 1 0 0 0 0 0 1 0 0 0 0 0 0 0 0
2001 2 Syasstro 7.6 W 10 1.39319 4 20 4 0 0 0 1 3 0 0 0 0 0 0 0 5 0 4 0 0 0 0 0 0
2001 2 Syasstro 8.6 N 10 1.51659 2 11 1 0 0 0 0 0 0 0 0 0 0 0 0 0 0 0 0 0 0 0 0 0
2001 2 Syasstro 9.6 W 10 1.62024 2 11 3 1 0 0 11 2 0 0 0 0 0 0 0 0 0 0 0 0 0 0 0 0
2001 2 Syasstro 11.7 W 10 1.82632 3 8 3 0 0 0 8 2 4 0 0 0 1 0 0 2 0 2 0 1 0 0 0 0
2001 2 Syasstro 12.8 W 10 1.68811 7 18 6 5 0 0 9 2 2 0 0 1 0 0 0 0 0 0 0 2 0 0 0 0
2001 2 Syasstro 14.2 N 10 1.70539 6 20 2 3 0 0 0 0 0 0 0 0 0 0 0 0 0 0 0 0 0 0 0 0
2001 2 Volkhov 0.4 W 10 1.78930 0 10 0 0 1 0 5 3 1 0 0 0 0 0 0 11 0 0 0 0 0 0 0 0
2001 2 Volkhov 1.4 W 10 1.89049 2 3 1 0 18 4 5 1 0 0 0 11 0 0 0 0 0 2 0 0 0 0 0 0
2001 2 Volkhov 1.8 W 10 1.39812 4 4 1 2 38 0 1 0 0 0 0 0 0 0 0 0 0 0 0 0 1 0 0 0
2001 2 Volkhov 2.6 W 10 1.49684 2 5 1 2 0 0 0 3 0 0 0 0 0 0 0 0 0 0 0 0 0 0 0 0
2001 2 Volkhov 1.9 S 10 1.82385 5 0 1 1 0 0 1 0 0 0 0 0 0 0 0 0 0 0 0 0 0 0 0 0
2001 2 Volkhov 3.5 W 10 1.64862 2 5 1 1 0 0 1 1 0 0 0 0 0 0 0 0 0 0 0 0 0 0 0 0
2001 2 Volkhov 4.2 W 10 1.72020 2 0 0 4 1 0 0 0 0 0 0 0 0 0 0 0 0 0 0 0 0 0 0 0
2001 2 Volkhov 8.5 N 10 1.77819 29 4 0 1 0 0 24 7 0 0 0 0 0 0 0 0 0 0 0 0 0 0 0 0
2001 2 Volkhov 4.3 S 10 1.75968 40 3 0 0 1 0 0 1 0 0 0 0 0 0 0 0 0 0 0 0 0 0 0 0
2001 2 Volkhov 11.6 N 10 1.98921 2 5 1 0 1 0 4 1 0 0 0 0 0 0 0 0 0 0 0 1 0 0 0 0
2001 2 Volkhov 9.4 S 10 2.01882 5 5 0 0 9 0 3 0 0 0 0 0 0 0 0 0 0 0 0 0 0 0 0 0
2001 2 Volkhov 13.9 N 10 1.57088 1 6 0 1 2 0 10 0 0 0 0 0 0 0 0 0 0 0 0 1 0 0 0 0
2001 2 Volkhov 13.0 S 10 1.21549 1 5 1 1 0 0 0 0 0 0 0 0 0 0 0 0 0 0 0 0 0 0 0 0
2001 2 Volkhov 15.9 N 10 1.47216 2 1 0 0 3 0 4 0 0 0 0 0 0 0 0 0 0 0 0 1 0 0 0 0
2001 2 Volkhov 15.3 S 10 2.90730 5 3 0 3 4 0 2 0 0 0 0 0 0 0 0 0 0 0 0 1 2 0 0 0
2002 1 Syasstro 0.3 N 10 1.14515 203 0 0 0 0 0 0 0 66 0 0 0 0 0 0 0 0 0 0 0 0 0 0 0
2002 1 Syasstro 2.7 W 10 1.43638 261 0 0 0 0 0 0 0 9 0 0 0 0 0 0 0 0 0 0 0 0 0 0 0
2002 1 Syasstro 3.2 N 10 2.93815 72 3 0 0 0 0 0 0 3 0 564 0 0 0 0 0 0 0 0 0 0 0 0 0
2002 1 Syasstro 3.5 W 10 1.18341 90 1 0 1 9 0 0 0 23 0 0 0 0 0 0 0 0 0 0 0 0 0 0 0
2002 1 Syasstro 5.0 N 10 1.33519 262 5 0 0 25 0 1 0 41 0 28 0 0 0 0 1 0 0 0 0 0 1 0 0
2002 1 Syasstro 5.2 W 10 1.93738 75 2 0 0 42 0 0 0 33 0 22 0 0 0 0 1 0 0 0 0 0 0 0 0
2002 1 Syasstro 7.6 W 10 1.24017 98 4 0 0 33 0 0 1 59 0 0 0 0 0 0 0 0 0 0 0 0 0 0 0
2002 1 Syasstro 8.6 N 10 1.87074 58 13 0 0 3 0 0 0 15 0 0 0 0 0 0 0 0 0 0 0 0 0 0 0
2002 1 Syasstro 9.6 W 10 1.42527 118 3 0 1 5 0 0 0 21 0 0 0 0 0 0 0 0 0 0 0 0 0 0 0
2002 1 Syasstro 11.7 W 10 1.62765 98 1 0 1 4 0 0 0 25 0 0 0 0 0 0 0 0 0 0 0 0 0 0 0
2002 1 Syasstro 12.8 W 10 1.85470 292 8 0 0 4 0 0 0 63 0 0 0 0 0 0 0 0 0 0 0 0 0 0 0
2002 1 Syasstro 14.2 N 10 1.78066 96 2 0 0 0 0 0 0 22 0 0 0 0 0 0 0 0 1 0 0 2 0 0 0
2002 1 Volkhov 0.4 W 10 1.28953 31 3 0 0 1 0 0 0 28 0 0 0 0 0 0 0 0 0 0 0 0 0 0 0
2002 1 Volkhov 1.4 W 10 1.54620 132 6 0 0 42 0 0 0 36 0 0 0 0 0 0 0 0 0 0 0 0 0 0 0
2002 1 Volkhov 1.8 W 10 2.01636 141 14 0 0 61 0 0 0 45 0 0 1 0 0 0 0 0 0 0 0 0 0 0 0
2002 1 Volkhov 2.6 W 10 1.99908 47 6 0 0 1 0 0 0 101 0 0 0 0 0 0 0 0 0 0 0 0 0 0 0
2002 1 Volkhov 1.9 S 10 1.32778 20 0 0 0 0 0 0 0 34 0 0 0 0 0 0 0 0 0 0 0 0 0 0 0
2002 1 Volkhov 3.5 W 10 2.00525 24 5 0 0 0 0 0 0 78 0 0 0 0 0 0 0 0 0 0 0 0 0 0 0
2002 1 Volkhov 4.2 W 10 1.81768 56 4 0 0 3 0 0 0 18 0 0 0 0 0 0 0 0 0 0 0 0 0 0 0
2002 1 Volkhov 8.5 N 10 1.97070 284 7 0 0 2 31 0 0 4 0 0 0 0 0 1 0 0 0 0 0 0 0 0
2002 1 Volkhov 4.3 S 10 1.52769 140 4 0 0 1 0 0 0 88 0 0 0 0 0 0 0 0 0 0 0 0 0 0 0
2002 1 Volkhov 11.6 N 10 1.67084 86 20 1 0 0 0 0 0 0 0 0 0 0 0 0 0 0 0 0 0 0 0 0 0
2002 1 Volkhov 9.4 S 10 1.80041 193 5 0 0 2 0 0 0 53 0 0 0 0 0 0 0 0 0 0 0 0 0 0 0
2002 1 Volkhov 13.9 N 10 1.78807 288 4 0 0 26 0 0 0 11 0 0 0 0 0 0 0 0 0 0 0 0 0 0 0
2002 1 Volkhov 13.0 S 10 1.92134 102 1 0 0 16 0 0 0 34 0 0 1 0 0 0 0 0 0 0 0 0 0 0 0
2002 1 Volkhov 15.9 N 10 1.79794 269 10 2 1 42 0 0 0 162 0 0 0 0 0 0 0 0 2 0 2 0 0 0 0
2002 1 Volkhov 15.3 S 10 1.70292 132 3 0 0 12 0 0 0 30 0 0 0 0 0 0 0 0 0 0 0 0 0 0 0
2002 2 Syasstro 0.3 N 10 2.99985 21 6 1 3 0 0 6 0 0 0 0 0 0 0 0 0 1 0 0 0 0 0 0 0
2002 2 Syasstro 2.7 W 10 1.64739 5 6 0 1 0 0 0 0 0 0 0 0 0 0 0 0 0 0 0 0 0 0 0 0
2002 2 Syasstro 3.2 N 10 1.15132 1 1 0 0 0 0 4 0 0 0 0 0 0 0 0 0 0 0 0 0 0 0 0 0
2002 2 Syasstro 3.5 W 10 1.04520 0 5 1 4 0 0 10 2 0 0 0 0 0 0 0 0 0 0 0 0 0 0 0 0
```

```
2002 2 Syasstro 5.0 N 10 1.37838 0 2 0 1 0 0 0 0 0 0 0 0 0 0 0 0 0 0 0 0 0 0 0 0
2002 2 Syasstro 5.2 W 10 2.37915 1 5 2 1 0 0 4 0 0 0 0 0 0 0 0 0 1 0 0 0 0 0 0
2002 2 Syasstro 7.6 W 10 1.63999 0 8 1 1 0 0 1 0 0 0 0 0 0 0 0 0 0 0 0 1 0 0 0
2002 2 Syasstro 8.6 N 10 1.05507 0 5 0 0 0 0 0 0 0 0 0 0 0 0 0 0 0 0 0 0 0 0 0 0
2002 2 Syasstro 9.6 W 10 1.41416 0 1 0 0 0 0 11 0 0 0 0 0 0 0 0 0 0 0 0 0 0 0 0 0
2002 2 Syasstro 11.7 W 10 1.59433 0 3 0 3 0 0 10 0 0 0 0 0 0 0 0 0 0 0 0 0 0 0 0 0
2002 2 Syasstro 12.8 W 10 1.66096 0 9 1 4 0 0 31 0 0 0 0 0 0 0 0 0 0 0 0 1 0 0 0 0
2002 2 Syasstro 14.2 N 10 1.59063 0 2 0 4 0 0 2 0 0 0 0 0 0 0 0 0 0 0 0 0 0 0 0 0
2002 2 Volkhov 0.4 W 10 1.42157 0 3 1 5 0 0 13 0 0 0 0 0 0 0 0 0 0 0 0 0 0 0 0
2002 2 Volkhov 1.4 W 10 1.36234 1 1 1 3 0 0 10 0 0 0 0 0 0 0 0 0 0 0 1 0 0 0 0
2002 2 Volkhov 1.8 W 10 2.00525 1 1 0 3 0 0 2 3 0 0 0 0 0 0 0 0 0 0 0 0 0 0 0
2002 2 Volkhov 2.6 W 10 1.22783 1 3 1 1 0 0 3 0 0 0 0 0 0 0 0 0 0 1 0 0 0 0 0 0
2002 2 Volkhov 1.9 S 10 1.46229 0 6 1 2 0 0 1 0 0 0 0 0 0 0 0 0 0 0 0 0 0 0 0
2002 2 Volkhov 3.5 W 10 1.63382 0 3 4 1 0 0 2 0 0 0 0 0 0 0 0 0 0 0 0 0 0 0 0
2002 2 Volkhov 4.2 W 10 1.64492 0 0 0 3 0 0 3 0 0 0 0 0 0 0 0 0 0 0 0 0 0 0 0
2002 2 Volkhov 8.5 N 10 1.36851 2 5 1 1 0 0 5 1 0 0 0 0 0 0 0 0 0 0 0 0 0 0 0
2002 2 Volkhov 4.3 S 10 1.42774 0 5 0 4 0 0 3 0 0 0 0 0 0 0 0 0 0 0 0 0 0 0 0
2002 2 Volkhov 11.6 N 10 1.50548 0 4 1 5 0 0 13 1 0 0 0 0 0 0 0 0 0 0 0 1 0 0 0 0
2002 2 Volkhov 9.4 S 10 1.41046 0 4 1 1 0 0 2 0 0 0 0 0 0 0 0 0 0 0 0 0 0 0 0
2002 2 Volkhov 13.9 N 10 1.94355 0 2 0 3 0 0 24 0 0 0 0 0 0 0 0 0 0 0 0 4 0 0 0 0
2002 2 Volkhov 13.0 S 10 0.84899 0 1 0 0 0 0 1 0 0 0 0 0 0 0 0 0 0 0 0 0 0 0 0 0
2002 2 Volkhov 15.9 N 10 1.41293 0 2 2 5 0 0 11 2 0 0 0 0 0 0 0 0 2 0 0 0 0 0 0
2002 2 Volkhov 15.3 S 10 1.18587 0 2 0 2 0 0 9 0 0 0 0 0 0 0 0 0 0 0 1 0 0 0 0
2003 1 Syasstro 0.3 N 10 2.34954 41 2 0 0 3 0 0 0 0 0 0 0 0 0 0 0 0 0 0 0 0 0 0 0
2003 1 Syasstro 2.7 W 10 1.42527 388 5 0 0 0 0 0 0 0 0 0 0 0 0 0 0 0 0 0 0 0 0 0 0
2003 1 Syasstro 3.2 N 10 1.20809 46 0 0 0 0 0 0 0 0 0 0 0 0 0 0 0 0 0 0 0 0 0 0 0
2003 1 Syasstro 3.5 W 10 1.75845 47 1 0 0 8 0 0 0 0 0 0 0 0 0 0 0 0 0 0 0 0 0 0 0
2003 1 Syasstro 5.0 N 10 1.44008 34 0 0 0 8 0 0 0 0 0 0 0 0 0 0 1 0 0 0 0 0 0 0 0
2003 1 Syasstro 5.2 W 10 0.94771 92 0 0 0 5 0 0 0 0 0 0 0 0 0 0 0 0 0 0 0 0 0 0 0
2003 1 Syasstro 7.6 W 10 1.14145 50 2 0 0 8 0 0 0 0 0 0 0 0 0 0 0 0 0 0 0 0 0 0 0
2003 1 Syasstro 8.6 N 10 0.68981 33 2 4 0 0 0 0 0 0 0 0 0 0 0 0 0 0 0 0 0 0 0 0 0
2003 1 Syasstro 9.6 W 10 1.70415 100 8 0 0 0 0 0 0 0 0 0 0 0 0 0 0 0 0 0 0 0 0 0 0
2003 1 Syasstro 11.7 W 10 0.91439 78 1 0 2 12 0 0 0 0 0 0 0 0 0 0 0 0 0 0 0 0 0 0 0
2003 1 Syasstro 12.8 W 10 1.28213 128 2 0 0 0 0 0 0 0 0 0 0 0 0 1 0 0 0 0 0 0 0 0
2003 1 Syasstro 14.2 N 10 0.87861 48 0 0 0 0 0 0 0 0 0 0 0 0 0 0 0 0 0 0 0 0 0 0 0
2003 1 Volkhov 0.4 W 10 1.12664 23 1 0 0 3 0 0 0 0 0 0 0 0 0 0 0 0 0 0 0 0 0 0
2003 1 Volkhov 1.4 W 10 1.15009 70 1 0 0 3 0 0 0 0 0 0 0 0 0 0 0 0 0 0 0 0 0 0
2003 1 Volkhov 1.8 W 10 1.62765 30 5 11 0 0 0 0 0 0 0 0 0 0 0 0 0 0 0 0 0 0 0 0 0
2003 1 Volkhov 2.6 W 10 1.06247 13 1 0 0 2 0 0 0 0 0 0 0 0 0 0 0 0 0 0 0 0 0 0
2003 1 Volkhov 1.9 S 10 1.49314 5 3 0 0 0 0 0 0 0 0 0 0 0 0 0 0 0 0 0 0 0 0 0
2003 1 Volkhov 3.5 W 10 2.06572 5 0 0 0 0 0 0 0 0 0 0 0 0 0 0 0 0 0 0 0 0 0 0
2003 1 Volkhov 4.2 W 10 3.43916 33 5 0 0 3 0 0 0 0 0 0 0 0 0 0 0 0 0 0 0 0 0 0
2003 1 Volkhov 8.5 N 10 0.95635 199 0 0 0 4 0 0 0 0 0 0 0 0 0 0 0 0 0 0 0 0 0 0
2003 1 Volkhov 4.3 S 10 1.12047 34 1 0 0 1 0 0 0 0 0 0 0 0 0 0 0 0 0 0 0 0 0 0
2003 1 Volkhov 11.6 N 10 1.03533 397 0 0 0 0 0 0 0 0 0 0 0 0 0 0 0 0 0 0 0 0 0 0 0
2003 1 Volkhov 9.4 S 10 0.98720 9 0 0 0 2 0 0 0 0 0 0 0 0 0 0 0 0 0 0 0 0 0
2003 1 Volkhov 13.9 N 10 1.50918 109 8 0 0 10 0 0 0 0 0 0 0 0 0 0 0 0 0 0 0 0 0 0 0
2003 1 Volkhov 13.0 S 10 0.73793 19 0 0 0 8 0 0 0 0 0 0 0 0 0 0 1 0 0 0 0 0 0 0 0
2003 1 Volkhov 15.9 N 10 1.33149 96 1 0 0 1 0 0 0 0 0 0 0 0 0 0 0 0 0 0 0 0 0 0
2003 1 Volkhov 15.3 S 10 1.08098 97 0 0 0 10 0 0 0 0 0 0 0 0 0 0 0 0 0 0 0 0 0 0 0
2003 2 Syasstro 0.3 N 10 1.84606 6 6 0 0 0 0 1 0 0 0 0 0 0 0 0 0 0 0 0 0 0 0 0
2003 2 Syasstro 2.7 W 10 2.10767 9 13 0 0 3 0 0 0 0 0 0 0 0 0 0 0 0 0 0 0 0 0 0
2003 2 Syasstro 3.2 N 10 1.20438 1 3 0 0 0 0 21 0 0 0 0 0 0 0 0 0 0 0 0 0 0 0 0
2003 2 Syasstro 3.5 W 10 1.03286 0 3 0 0 1 0 2 0 0 0 0 0 0 0 0 0 0 0 1 0 0 0 0
2003 2 Syasstro 5.0 N 10 1.29817 1 3 0 0 4 0 7 0 0 0 0 0 0 0 0 0 0 0 0 0 0 0 0
2003 2 Syasstro 5.2 W 10 1.15379 0 7 0 0 0 0 8 0 0 0 0 0 0 0 0 0 0 0 0 0 0 0 0
2003 2 Syasstro 7.6 W 10 1.23153 0 4 0 0 12 0 4 0 0 0 0 0 0 0 0 0 0 0 0 3 0 0 0 0
2003 2 Syasstro 8.6 N 10 1.66590 0 4 0 0 0 0 1 0 0 0 0 0 0 0 0 0 0 0 0 0 0 0 0
2003 2 Syasstro 9.6 W 10 1.28583 0 5 0 0 0 0 1 2 0 0 0 0 0 0 0 0 0 0 0 0 0 0 0
2003 2 Syasstro 11.7 W 10 1.09332 0 0 0 0 0 0 1 0 0 0 0 0 0 0 0 0 0 0 0 5 0 0 0 0
2003 2 Syasstro 12.8 W 10 1.17970 0 5 0 0 3 0 2 0 0 0 0 0 0 0 0 0 1 0 0 1 0 0 0 0
2003 2 Syasstro 14.2 N 10 1.64369 0 2 0 0 1 0 0 0 0 0 0 0 0 0 0 0 0 0 0 0 0 0 0 0
2003 2 Volkhov 0.4 W 10 0.96622 0 2 1 0 2 0 1 0 0 0 0 0 0 0 0 0 1 0 0 1 0 0 0 0
2003 2 Volkhov 1.4 W 10 1.19451 0 6 0 0 11 0 0 0 0 0 0 0 0 0 0 0 0 0 0 0 0 0 0
2003 2 Volkhov 1.8 W 10 0.74534 0 4 0 0 6 0 0 0 0 0 0 0 0 0 0 0 0 0 0 0 0 0 0
2003 2 Volkhov 2.6 W 10 0.71572 0 3 0 0 0 0 8 0 0 0 0 0 0 0 1 0 0 0 0 0 0 0 0
2003 2 Volkhov 1.9 S 10 1.19081 0 2 0 0 4 0 1 0 0 0 0 0 0 0 0 0 0 0 2 0 0 0 0
2003 2 Volkhov 3.5 W 10 1.06618 0 4 1 1 3 0 1 0 0 0 0 0 0 0 0 0 0 0 0 0 0 0 0
2003 2 Volkhov 4.2 W 10 1.02422 0 7 3 0 3 0 1 0 0 0 0 0 0 0 0 0 0 0 0 0 0 0 0
2003 2 Volkhov 8.5 N 10 0.80827 0 2 0 0 0 0 1 1 0 0 0 0 0 0 0 0 0 0 1 0 0 0 0
2003 2 Volkhov 4.3 S 10 1.19204 0 0 0 0 1 0 1 0 0 0 0 0 0 0 0 0 0 0 0 0 0 0 0
2003 2 Volkhov 11.6 N 10 1.93244 0 1 0 0 0 0 5 3 0 0 0 0 0 0 0 0 0 0 2 0 0 0 0
2003 2 Volkhov 9.4 S 10 1.28583 0 1 0 0 2 0 0 0 0 0 0 0 0 0 0 0 0 0 0 0 0 0 0
2003 2 Volkhov 13.9 N 10 1.39936 0 7 0 0 1 0 12 0 0 0 0 0 0 0 0 0 0 0 0 7 0 0 0 0
2003 2 Volkhov 13.0 S 10 0.85516 0 1 0 0 4 0 0 0 0 0 0 0 0 0 0 0 0 0 0 4 0 0 0 0
2003 2 Volkhov 15.9 N 10 1.27225 0 5 0 1 4 0 2 0 0 0 0 0 0 0 0 0 0 0 0 0 0 0 0 0
2003 2 Volkhov 15.3 S 10 0.91933 0 3 0 0 3 0 0 0 0 0 0 0 0 0 0 0 0 0 0 3 0 0 0 0
2004 1 Syasstro 0.3 N 9 1.81275 3 0 0 0 0 0 0 0 0 0 0 0 0 0 0 0 0 0 0 0 0 0 0
2004 1 Syasstro 2.7 W 10 2.08793 4 0 0 0 0 0 0 0 0 0 0 0 0 0 0 0 0 0 0 0 0 0 0 0
2004 1 Syasstro 3.2 N 10 1.71156 0 0 0 0 0 0 0 0 0 0 0 0 0 0 0 0 0 0 0 0 0 0 0 0
2004 1 Syasstro 3.5 W 10 1.67824 1 0 0 0 0 0 0 0 0 0 0 0 0 0 0 0 0 0 0 0 0 0 0 0
2004 1 Syasstro 5.0 N 10 1.68564 4 0 0 0 0 0 0 0 0 0 0 0 0 0 0 0 0 0 0 0 0 0 0 0
2004 1 Syasstro 5.2 W 10 1.92010 5 0 0 0 0 0 0 0 0 0 0 0 0 0 0 0 0 0 0 0 0 0 0 0
2004 1 Syasstro 7.6 W 10 1.67824 0 0 0 0 0 0 0 0 0 0 0 0 0 0 0 0 0 0 0 0 0 0 0 0
2004 1 Syasstro 8.6 N 10 1.81892 0 0 0 0 0 0 0 0 0 0 0 0 0 0 0 0 0 0 0 0 0 0 0 0
2004 1 Syasstro 9.6 W 10 1.64616 1 1 0 0 0 0 0 0 0 0 0 0 0 0 0 0 0 0 0 0 0 0 0 0
2004 1 Syasstro 11.7 W 10 1.79300 3 0 0 0 0 0 0 0 0 0 0 0 0 0 0 0 0 0 0 0 0 0 0 0
2004 1 Syasstro 12.8 W 10 1.72390 3 0 0 0 0 0 0 0 0 0 0 0 0 0 0 0 0 0 0 0 0 0 0 0
```

```
2004 1 Syasstro 14.2 N 10 1.61654 0 0 0 0 0 0 0 0 0 0 0 0 0 0 0 0 0 0 0 0 0 0 0 0
2004 1 Volkhov 0.4 W 10 1.46599 2 0 0 0 0 0 0 0 0 0 0 0 0 0 0 0 0 0 0 0 0 0 0 0
2004 1 Volkhov 1.4 W 10 1.35863 0 1 0 0 0 0 0 0 0 0 0 0 0 0 0 0 0 0 0 0 0 0 0 0
2004 1 Volkhov 1.8 W 10 1.85223 6 1 0 0 0 0 0 0 0 0 0 0 0 0 0 0 0 0 0 0 0 0 0 0
2004 1 Volkhov 2.6 W 10 1.51905 6 0 0 0 0 0 0 0 0 0 0 0 0 0 0 0 0 0 0 0 0 0 0 0
2004 1 Volkhov 1.9 S 10 2.06942 7 0 0 0 0 0 0 0 0 0 0 0 0 0 0 0 0 0 0 0 0 0 0 0
2004 1 Volkhov 3.5 W 10 1.69305 4 2 0 0 0 0 0 0 0 0 0 0 0 0 0 0 0 0 0 0 0 0 0 0
2004 1 Volkhov 4.2 W 10 2.27673 4 0 0 0 0 0 0 0 0 0 0 0 0 0 0 0 0 0 0 0 0 0 0 0
2004 1 Volkhov 8.5 N 10 1.82138 2 0 0 0 0 0 0 0 0 0 0 0 0 0 0 0 0 0 0 0 0 0 0 0
2004 1 Volkhov 4.3 S 10 1.69305 2 0 0 0 0 0 0 0 0 0 0 0 0 0 0 0 0 0 0 0 0 0 0 0
2004 1 Volkhov 11.6 N 10 1.87815 0 0 0 0 0 0 0 0 0 0 0 0 0 0 0 0 0 0 0 0 0 0 0 0
2004 1 Volkhov 9.4 S 10 1.92504 3 0 0 0 0 0 0 0 0 0 0 0 0 0 0 0 0 0 0 0 0 0 0 0
2004 1 Volkhov 13.9 N 10 2.46677 2 0 0 0 0 0 0 0 0 0 0 0 0 0 0 0 0 0 0 0 0 0 0 0
2004 1 Volkhov 13.0 S 10 1.72637 0 0 0 0 0 0 0 0 0 0 0 0 0 0 0 0 0 0 0 0 0 0 0 0
2004 1 Volkhov 15.9 N 10 1.30557 5 1 0 0 0 0 0 0 0 0 0 0 0 0 0 0 0 0 0 0 0 0 0 0
2004 1 Volkhov 15.3 S 10 1.41416 0 0 0 0 0 0 0 0 0 0 0 0 0 0 0 0 0 0 0 0 0 0 0 0
2004 2 Syasstro 0.3 N 10 2.12988 0 0 5 1 0 0 1 0 0 0 0 0 0 0 0 1 1 0 0 0 0 0 0 0
2004 2 Syasstro 2.7 W 10 2.86535 0 4 0 0 0 0 0 0 0 0 0 0 0 0 0 1 0 0 0 0 0 0 0 0
2004 2 Syasstro 3.2 N 10 1.71156 0 1 0 2 0 0 1 0 0 0 0 0 0 0 0 0 0 0 0 0 0 0 0 0
2004 2 Syasstro 3.5 W 10 1.81028 0 3 0 2 13 0 3 0 0 0 0 0 0 0 0 0 0 0 0 1 0 0 0 0
2004 2 Syasstro 5.0 N 10 1.77573 0 1 0 2 15 0 4 1 0 0 0 0 0 0 0 0 0 0 0 0 0 0 0 0
2004 2 Syasstro 5.2 W 10 1.94355 0 9 29 2 0 0 6 0 0 0 0 0 0 0 0 0 0 0 0 0 0 0 0 0
2004 2 Syasstro 7.6 W 10 1.67701 0 3 0 3 1 0 1 0 0 0 0 0 0 0 0 0 0 0 0 3 0 0 0 0
2004 2 Syasstro 8.6 N 10 1.59803 0 0 0 4 0 0 0 0 0 0 0 0 0 0 0 0 0 0 0 0 0 0 0 0
2004 2 Syasstro 9.6 W 10 1.53139 0 6 0 0 2 0 1 0 0 0 0 0 0 0 0 0 0 0 0 0 0 0 0 0
2004 2 Syasstro 11.7 W 10 1.75845 0 5 0 2 3 0 5 0 0 0 0 0 0 0 0 0 0 0 0 1 0 0 0 0
2004 2 Syasstro 12.8 W 10 1.55607 0 2 1 3 0 0 0 0 0 0 0 0 0 0 0 0 0 0 0 0 0 0 0 0
2004 2 Syasstro 14.2 N 10 1.84853 0 1 0 0 0 0 0 0 0 0 0 0 0 0 0 0 0 0 0 0 0 0 0 0
2004 2 Volkhov 0.4 W 10 1.42404 0 1 4 3 15 0 3 0 0 0 0 0 0 0 0 0 0 0 0 0 0 0 0 0
2004 2 Volkhov 1.4 W 10 1.46476 0 2 0 2 36 0 1 0 0 0 0 0 0 0 0 0 0 0 0 0 0 0 0 0
2004 2 Volkhov 1.8 W 10 1.38948 0 1 0 1 9 0 1 0 0 0 0 0 0 0 0 0 0 0 0 0 0 0 0 0
2004 2 Volkhov 2.6 W 10 1.52893 0 1 0 2 1 0 0 0 0 0 0 0 0 0 0 0 0 0 0 0 0 0 0 0
2004 2 Volkhov 1.9 S 10 1.47216 0 2 1 2 1 0 6 0 0 0 0 0 0 0 0 1 0 0 0 0 0 0 0 0
2004 2 Volkhov 3.5 W 10 1.84853 0 6 3 1 0 3 0 0 0 0 0 0 0 0 0 0 0 0 0 0 0 0 0 0
2004 2 Volkhov 4.2 W 10 2.38409 0 0 1 3 0 0 1 0 0 0 0 0 0 0 0 0 0 0 0 0 0 0 0 0
2004 2 Volkhov 8.5 N 10 0.96869 0 0 0 0 0 0 4 6 0 0 0 0 0 0 0 0 0 0 0 2 0 0 0 0
2004 2 Volkhov 4.3 S 10 1.69552 0 1 0 0 0 0 0 0 0 0 0 0 0 0 0 0 0 0 0 0 0 0 0 0
2004 2 Volkhov 11.6 N 10 1.82755 0 1 0 4 0 0 7 0 0 0 0 0 0 0 0 0 0 0 0 0 0 0 0 0
2004 2 Volkhov 9.4 S 10 2.02870 0 1 0 0 0 0 1 0 0 0 0 0 0 0 0 0 0 0 0 0 0 0 0 0
2004 2 Volkhov 13.9 N 10 3.63783 0 4 0 4 7 0 9 0 0 0 0 0 0 0 0 0 0 0 0 0 0 0 0 0
2004 2 Volkhov 13.0 S 10 1.89296 0 0 1 3 9 0 0 0 0 0 0 0 0 0 0 0 0 0 0 0 0 0 0 0
2004 2 Volkhov 15.9 N 10 1.16613 0 5 0 7 28 0 6 0 0 0 0 0 0 0 0 0 0 0 0 1 0 0 0 0
2004 2 Volkhov 15.3 S 10 1.15256 0 0 0 0 0 0 6 0 0 0 0 0 0 0 0 0 0 0 0 2 0 0 0 0
2005 1 Syasstro 0.3 N 10 1.75845 8 6 0 0 5 0 0 0 13 0 0 0 0 0 0 1 0 0 0 0 0 0 0 0
2005 1 Syasstro 2.7 W 10 1.46723 6 5 0 0 1 0 0 0 54 0 0 0 0 0 0 0 0 0 0 0 0 0 0 0
2005 1 Syasstro 3.5 W 10 1.48327 13 2 0 0 22 0 0 0 3 0 1 0 0 0 0 0 0 0 0 0 0 0 0 0
2005 1 Syasstro 5.0 N 10 1.97193 29 2 0 0 52 0 0 0 1 0 0 0 0 0 0 0 0 0 0 0 0 0 0 0
2005 1 Syasstro 5.2 W 10 1.46476 46 2 0 0 461 0 0 0 37 0 0 0 0 0 0 0 0 0 0 0 0 0 0 0
2005 1 Syasstro 7.6 W 10 1.83372 52 2 0 1 13 0 0 0 46 0 0 0 0 0 0 18 0 0 0 0 0 0 0 0
2005 1 Syasstro 8.6 N 10 1.28089 19 0 0 0 17 1 0 0 0 0 0 0 0 0 0 0 0 0 0 0 0 0 0 0
2005 1 Syasstro 9.6 W 10 3.23678 9 0 0 0 24 0 0 0 21 0 1 0 0 0 0 0 0 0 0 0 0 0 0 0
2005 1 Syasstro 11.7 W 10 1.79547 26 1 0 3 72 0 0 0 6 0 0 0 0 0 0 0 0 0 0 0 0 0 0 0
2005 1 Syasstro 12.8 W 10 1.89419 88 0 0 1 3 0 0 0 5 0 0 0 0 0 0 0 0 0 0 0 0 0 0 0
2005 1 Syasstro 14.2 N 10 1.75845 14 13 0 0 1 0 0 0 11 0 0 0 0 0 0 0 0 0 0 0 0 0 0 0
2005 1 Volkhov 0.4 W 10 1.63875 2 0 0 0 4 0 0 0 0 0 0 0 0 0 0 0 0 0 0 0 0 0 0 0
2005 1 Volkhov 1.4 W 10 1.64492 5 1 0 0 45 0 0 0 0 0 0 0 0 0 0 0 0 0 0 0 0 0 0 0
2005 1 Volkhov 1.8 W 10 1.72266 27 2 0 0 47 0 0 0 0 0 0 0 0 0 0 0 0 0 0 0 0 0 0 0
2005 1 Volkhov 2.6 W 10 1.99785 2 4 0 0 3 0 0 0 1 0 0 0 0 0 1 0 0 0 0 0 0 0 0 0
2005 1 Volkhov 1.9 S 10 2.00155 1 9 0 0 0 0 0 0 0 0 0 0 0 0 0 0 0 0 0 0 0 0 0 0
2005 1 Volkhov 3.5 W 10 1.69305 0 18 0 0 0 0 0 0 22 0 0 0 0 0 0 0 0 0 0 0 0 0 0 0
2005 1 Volkhov 4.2 W 10 1.47216 3 6 0 0 0 0 1 0 2 0 0 0 0 0 0 1 0 0 0 0 0 0 0 0
2005 1 Volkhov 8.5 N 10 2.14099 24 3 3 3 27 0 0 0 6 0 1 0 0 0 0 1 0 0 0 0 0 0 0 0
2005 1 Volkhov 4.3 S 10 1.77449 21 1 0 0 0 0 0 0 4 0 0 0 0 0 0 0 0 0 0 0 0 0 0 0
2005 1 Volkhov 11.6 N 10 1.89296 405 21 0 1 8 0 0 0 0 0 0 1 0 0 0 23 0 0 0 0 0 0 0 0
2005 1 Volkhov 9.4 S 10 1.70045 45 3 0 0 128 0 0 0 12 0 0 0 0 0 0 0 0 0 0 0 0 0 0 0
2005 1 Volkhov 13.9 N 10 2.31375 82 6 0 0 18 0 0 0 15 0 2 0 0 0 0 0 0 0 0 0 0 0 0 0
2005 1 Volkhov 13.0 S 10 1.37097 87 3 0 0 24 0 0 0 0 0 0 0 0 0 0 0 0 0 0 0 1 0 0 0
2005 1 Volkhov 15.9 N 10 2.24465 41 4 0 0 306 0 0 0 16 0 0 0 0 0 0 0 0 0 0 0 0 0 0 0
2005 1 Volkhov 15.3 S 10 2.01512 54 3 0 0 21 0 0 0 2 0 0 0 0 0 0 0 0 0 0 0 0 0 0 0
2005 2 Syasstro 0.3 N 10 1.63382 0 12 1 0 7 0 6 0 0 0 0 0 0 0 0 0 0 0 0 0 0 0 0 0
2005 2 Syasstro 2.7 W 10 2.03240 2 11 0 0 0 0 5 0 0 0 0 0 0 0 0 0 0 0 0 0 0 0 0 0
2005 2 Syasstro 3.5 W 10 2.15703 3 15 2 8 11 0 0 0 0 0 0 0 0 0 1 0 0 0 1 1 0 0 0 0
2005 2 Syasstro 5.0 N 10 3.07883 14 16 8 8 75 0 2 0 0 0 0 0 0 0 0 0 0 0 0 1 1 0 0 1
2005 2 Syasstro 5.2 W 10 2.15333 1 5 3 0 170 0 1 1 0 0 0 0 0 0 0 3 0 0 0 1 0 0 0 0
2005 2 Syasstro 7.6 W 10 2.61361 5 7 2 2 59 0 0 0 0 0 0 0 0 0 0 0 0 0 0 1 0 0 0 0
2005 2 Syasstro 8.6 N 10 2.23107 0 5 0 2 11 0 6 0 0 0 0 0 0 0 0 0 0 0 0 0 0 0 0 0
2005 2 Syasstro 9.6 W 10 2.81722 4 6 7 0 0 0 5 0 0 0 0 1 0 0 0 0 0 0 0 4 0 0 0 0
2005 2 Syasstro 11.7 W 10 1.94108 3 3 0 0 2 0 0 0 0 0 0 0 0 0 0 1 0 0 0 2 0 0 0 0
2005 2 Syasstro 12.8 W 10 2.74935 6 18 1 2 0 0 8 0 0 0 0 0 0 0 0 1 1 0 0 2 0 0 0 0
2005 2 Syasstro 14.2 N 10 3.05292 3 4 4 3 2 0 3 0 0 0 0 0 0 0 0 0 1 0 0 0 0 0 0 0
2005 2 Volkhov 0.4 W 10 1.76585 0 6 0 4 0 0 0 0 0 0 0 0 0 0 0 0 0 0 0 0 0 0 0 0
2005 2 Volkhov 1.4 W 10 1.89913 0 4 1 39 0 0 4 0 0 0 0 0 0 0 0 0 0 0 0 0 0 0 0 0
2005 2 Volkhov 1.8 W 10 2.31992 1 7 3 9 43 0 1 1 0 0 0 0 0 0 0 0 0 0 0 0 0 0 0 0
2005 2 Volkhov 2.6 W 10 1.95342 0 4 0 6 0 0 1 0 0 0 0 0 0 0 0 0 0 0 0 0 0 0 0 0
2005 2 Volkhov 1.9 S 10 1.88308 0 7 0 7 0 0 0 0 0 0 0 0 0 0 0 0 0 0 0 0 0 0 0 0
2005 2 Volkhov 3.5 W 10 2.16197 0 12 4 5 0 0 0 0 0 0 0 0 0 0 0 0 0 0 0 0 0 0 0 0
2005 2 Volkhov 4.2 W 10 1.72266 0 12 0 1 1 0 1 0 0 0 0 0 0 0 0 0 0 0 0 0 0 0 0 0
2005 2 Volkhov 8.5 N 10 2.67531 0 1 1 2 0 0 6 0 0 0 0 0 0 0 0 0 0 0 0 1 0 0 0 0
```

```
2005 2 Volkhov 4.3 S 10 1.88185 0 10 0 4 3 0 0 0 0 0 0 0 0 0 1 0 0 0 0 0 0 0 0
2005 2 Volkhov 11.6 N 10 2.50008 3 10 4 1 4 0 4 0 0 0 0 0 0 0 0 0 0 0 0 0 0 0 0
2005 2 Volkhov 9.4 S 10 1.39565 0 1 0 6 3 0 17 0 0 0 0 0 0 0 0 0 0 0 1 0 0 0 0
2005 2 Volkhov 13.9 N 10 2.56549 0 5 1 1 12 0 0 0 0 0 0 0 0 0 0 0 0 0 1 2 0 0 0
2005 2 Volkhov 13.0 S 10 2.34213 0 4 0 1 0 0 0 0 0 0 0 0 0 0 0 0 0 0 0 0 0 0 0
2005 2 Volkhov 15.9 N 10 2.33473 0 9 0 8 16 0 6 0 0 0 0 0 0 0 0 0 0 1 0 0 0 0 0 0
2005 2 Volkhov 15.3 S 10 2.02623 0 3 1 1 4 0 0 0 0 0 0 0 0 0 0 0 0 0 1 0 0 0 0
2017 1 Syasstro 0.3 N 10 1.47093 0 1 0 0 0 0 0 0 15 0 0 0 0 0 0 0 0 0 0 0 0 0 0
2017 1 Syasstro 2.7 W 10 1.55607 0 1 0 0 1 0 0 0 33 0 0 0 0 0 0 0 0 0 0 0 0 0 0
2017 1 Syasstro 3.5 W 10 1.82262 6 2 0 0 11 0 0 0 27 0 0 0 0 0 0 0 0 0 0 0 0 0 0
2017 1 Syasstro 5.0 N 10 1.64245 2 0 0 0 106 0 0 0 42 0 0 0 0 0 0 0 0 0 0 0 0 0 0
2017 1 Syasstro 5.2 W 10 1.53756 0 1 0 0 17 0 0 0 69 0 0 0 0 0 0 0 0 0 0 0 0 0 0
2017 1 Syasstro 7.6 W 10 1.81645 7 3 0 0 11 0 0 0 44 0 0 0 0 0 0 0 0 0 0 0 0 0 0
2017 1 Syasstro 8.6 N 10 1.84360 2 4 0 0 17 2 0 0 22 0 0 0 0 0 0 0 0 0 0 0 0 0 0
2017 1 Syasstro 9.6 W 10 1.96329 1 3 0 0 6 0 0 0 44 0 0 0 0 0 0 0 0 0 0 0 0 0 0
2017 1 Syasstro 11.7 W 10 1.66960 4 1 0 0 6 0 0 0 30 0 0 0 0 0 0 0 0 0 0 0 0 0 0
2017 1 Syasstro 12.8 W 10 1.46352 33 0 0 0 0 0 0 0 0 0 0 0 0 0 0 0 0 0 0 0 0 0 0
2017 1 Syasstro 14.2 N 10 1.80781 11 7 0 0 14 0 0 0 72 0 0 0 0 0 0 0 0 0 0 0 0 0 0
2017 1 Volkhov 1.4 W 10 1.33395 9 3 0 0 0 0 0 0 66 0 0 0 0 0 0 0 0 0 0 0 0 0 0
2017 1 Volkhov 1.8 W 10 1.68811 0 3 0 0 1 1 0 0 0 0 0 0 0 0 1 0 0 0 0 0 0 0 0
2017 1 Volkhov 2.6 W 10 1.57582 0 2 0 0 0 0 0 0 15 0 0 0 0 0 0 0 0 0 0 0 0 0 0
2017 1 Volkhov 1.9 S 10 0.96005 0 3 0 0 0 0 0 0 11 0 0 0 0 0 0 0 0 0 0 0 0 0 0
2017 1 Volkhov 3.5 W 10 1.56595 0 17 0 0 0 0 0 0 1 0 0 0 0 0 0 0 0 0 0 0 0 0 0
2017 1 Volkhov 4.2 W 4 0.52445 0 0 0 0 0 0 0 0 0 0 0 0 0 0 0 0 0 0 0 0 0 0 0
2017 1 Volkhov 8.5 N 10 1.73624 46 5 0 0 23 0 0 0 6 0 0 0 0 0 0 1 0 0 0 0 0 0 0 0
2017 1 Volkhov 4.3 S 10 1.61654 1 0 0 0 0 0 0 0 20 0 0 0 0 0 0 0 0 0 0 0 0 0 0
2017 1 Volkhov 11.6 N 10 1.68811 2 16 0 0 6 0 0 0 43 0 0 0 0 0 0 0 0 0 0 0 0 0 0
2017 1 Volkhov 9.4 S 10 1.40676 29 5 0 0 3 0 0 0 28 0 0 0 0 0 0 0 0 0 0 0 0 0 0
2017 1 Volkhov 13.9 N 10 1.54250 4 6 0 0 12 0 0 0 12 0 0 0 0 0 0 0 0 0 0 0 0 0 0
2017 1 Volkhov 13.0 S 10 1.67330 9 0 0 0 1 0 0 0 63 0 0 0 0 0 0 0 0 0 0 0 0 0 0
2017 1 Volkhov 15.9 N 10 1.98797 26 6 0 0 27 0 0 0 142 0 0 2 0 0 0 1 0 0 0 0 0 0 0 0
2017 1 Volkhov 15.3 S 10 1.59926 1 1 0 0 9 0 0 0 18 0 0 0 0 0 0 0 0 0 0 0 0 0 0
2017 2 Syasstro 0.3 N 10 1.40799 1 2 1 1 0 0 4 0 5 1 0 0 10 0 0 1 0 0 0 0 0 0 0 0
2017 2 Syasstro 2.7 W 10 1.43638 1 5 0 0 5 0 3 0 0 0 0 0 0 0 0 1 1 0 0 0 0 0 0 0
2017 2 Syasstro 3.5 W 10 1.45859 0 2 8 2 17 0 12 1 0 0 0 0 0 0 0 0 0 0 0 0 2 0 0 0
2017 2 Syasstro 5.0 N 10 1.36480 1 5 5 0 35 0 6 0 0 0 0 1 0 0 0 0 0 0 0 0 1 0 0 0
2017 2 Syasstro 5.2 W 10 2.02499 0 17 21 0 106 0 6 0 1 0 0 1 0 0 0 1 0 0 0 0 0 0 0 0
2017 2 Syasstro 7.6 W 10 2.06942 5 5 4 2 27 0 0 0 0 0 0 1 1 0 0 1 0 0 0 0 0 0 2 0
2017 2 Syasstro 8.6 N 10 1.65850 0 16 4 5 28 0 4 0 0 0 0 0 0 0 0 1 1 0 0 0 1 0 0 0
2017 2 Syasstro 9.6 W 10 1.77079 0 8 20 0 2 0 1 0 0 0 0 0 0 0 0 1 1 0 0 0 0 0 0 0
2017 2 Syasstro 11.7 W 10 1.60050 13 5 10 0 3 0 3 5 0 0 12 0 1 0 0 17 2 0 0 0 0 0 0 0 0
2017 2 Syasstro 12.8 W 10 1.60297 0 1 8 0 0 0 0 0 0 0 0 0 0 0 0 2 0 0 0 0 0 0 0 0
2017 2 Syasstro 14.2 N 10 1.91393 0 4 4 0 11 0 0 0 0 0 0 0 2 2 0 0 0 0 0 0 0 0 0 0
2017 2 Volkhov 1.4 W 10 1.63752 1 2 1 1 2 0 3 0 2 0 0 0 0 0 0 0 1 0 0 0 0 0 0 0
2017 2 Volkhov 1.8 W 10 1.98427 1 3 1 0 12 0 1 0 0 0 0 0 0 0 0 0 0 0 0 0 1 0 0 0
2017 2 Volkhov 2.6 W 10 1.48574 1 5 1 0 0 0 1 0 0 0 0 0 1 0 0 0 0 0 0 0 0 0 0
2017 2 Volkhov 1.9 S 10 1.55607 0 0 5 3 0 0 6 0 0 0 0 0 0 0 0 0 1 0 0 0 0 0 0 0
2017 2 Volkhov 3.5 W 10 1.39812 0 2 0 2 0 0 0 0 0 0 0 0 0 0 0 0 0 0 0 0 0 0 0 0
2017 2 Volkhov 8.5 N 4 0.68734 0 0 0 0 0 0 3 0 0 0 0 0 0 0 0 2 0 0 0 0 0 0 0 0
2017 2 Volkhov 4.3 S 10 1.76462 2 3 3 0 9 0 5 0 2 0 0 1 0 0 0 1 0 0 0 0 0 0 1 0
2017 2 Volkhov 11.6 N 10 1.20685 0 0 0 0 4 0 1 0 0 0 0 1 0 0 0 1 0 0 0 0 0 0 0 0
2017 2 Volkhov 9.4 S 10 1.94232 9 5 7 1 3 0 0 0 0 0 0 0 0 0 0 0 0 0 0 0 1 0 0 0
2017 2 Volkhov 13.9 N 10 1.47093 0 4 2 0 16 0 3 0 0 0 0 6 0 0 0 2 0 0 0 2 0 0 0 0
2017 2 Volkhov 13.0 S 10 1.68441 0 4 7 1 0 0 2 0 6 0 0 0 0 0 0 0 0 0 0 0 0 0 0 0
2017 2 Volkhov 15.9 N 10 1.34753 0 7 5 2 3 0 5 0 0 0 0 1 0 0 0 0 0 0 0 0 0 0 0 0
2017 2 Volkhov 15.3 S 10 1.52276 0 10 20 1 39 0 4 0 0 0 0 1 0 0 0 0 0 0 0 6 0 0 0 0
2025 1 Syasstro 0.3 N 10 1.08345 5 0 0 0 1 0 0 0 29 0 0 0 0 0 0 0 1 0 0 0 0 0 0 0
2025 1 Syasstro 2.7 W 10 1.63752 1 2 0 0 25 0 0 0 4 0 0 0 0 0 0 0 0 0 0 0 0 0 0 0
2025 1 Syasstro 3.5 W 10 1.91147 0 1 0 0 44 0 0 0 0 0 0 0 0 0 0 0 0 0 0 0 0 0 0 0
2025 1 Syasstro 5.2 W 10 1.61777 2 0 0 0 43 0 0 0 10 0 0 0 0 0 0 0 0 0 0 0 0 0 0 0
2025 1 Syasstro 7.6 W 10 2.25575 2 0 0 0 74 0 0 0 11 0 12 0 0 0 0 0 0 0 0 0 0 0 0 0
2025 1 Syasstro 8.6 N 10 1.74364 9 0 2 1 129 0 0 8 5 0 6 0 0 0 0 0 0 0 0 0 0 0 0
2025 1 Syasstro 9.6 W 10 1.63999 0 3 1 0 72 0 0 4 5 0 0 0 0 0 0 0 0 0 0 0 0 0 0
2025 1 Syasstro 11.7 W 10 2.15086 0 1 0 0 10 0 0 0 0 0 0 0 0 0 0 0 0 0 0 0 0 0 0
2025 1 Syasstro 12.8 W 10 1.64492 1 0 0 0 194 0 0 0 12 0 0 0 0 0 0 0 0 0 0 0 0 0 0 0
2025 1 Syasstro 14.2 N 10 1.65603 2 1 0 0 23 0 0 0 16 0 0 0 0 0 0 0 0 0 0 0 0 0 0
2025 1 Volkhov 1.4 W 10 1.27102 2 0 0 0 32 0 0 0 2 0 0 0 0 0 0 0 0 0 0 0 0 0 0
2025 1 Volkhov 1.8 W 10 2.56178 4 2 2 0 46 0 0 0 3 0 0 0 0 0 0 0 0 0 0 0 0 0 0
2025 1 Volkhov 1.9 S 10 0.91810 0 1 0 0 0 0 0 0 0 0 0 0 0 0 0 0 0 0 0 0 0 0 0
2025 1 Volkhov 3.5 W 10 1.15626 0 2 4 0 11 0 0 0 3 0 0 0 0 0 0 0 0 0 0 0 0 0 0
2025 1 Volkhov 4.2 W 5 0.67130 0 0 0 0 0 0 0 0 4 0 0 0 0 0 0 0 0 0 0 0 0 0 0
2025 1 Volkhov 8.5 N 10 2.38902 1 3 2 0 52 0 0 0 4 0 0 0 0 0 0 0 0 0 0 0 0 0 0
2025 1 Volkhov 4.3 S 10 1.08715 44 0 1 1 6 0 0 0 11 0 0 0 0 0 0 0 0 0 0 0 0 0 0
2025 1 Volkhov 11.6 N 10 2.50379 7 0 1 0 120 0 0 0 20 0 0 0 0 0 0 0 0 0 0 0 0 0 0
2025 1 Volkhov 9.4 S 10 0.96992 1 0 0 0 59 0 0 0 1 0 0 0 0 0 0 0 0 0 0 0 0 0 0
2025 1 Volkhov 13.9 N 10 3.32686 9 4 7 0 64 0 0 0 29 0 0 0 0 0 0 0 3 0 0 0 0 0 0 0
2025 1 Volkhov 15.9 N 10 1.73130 8 1 0 0 63 0 0 0 6 0 0 0 0 0 0 0 2 0 0 0 0 0 0 0
2025 1 Volkhov 15.3 S 10 1.29447 2 2 0 0 42 0 0 0 0 0 0 0 0 0 0 0 0 0 0 1 0 0 0
2025 2 Syasstro 0.3 N 10 0.72189 0 4 1 3 0 0 0 0 3 0 0 0 0 0 0 0 0 0 0 0 0 0 0
2025 2 Syasstro 2.7 W 10 0.80333 1 2 0 3 5 0 2 0 1 0 0 0 0 0 0 0 1 0 0 0 0 0 0 0
2025 2 Syasstro 3.5 W 10 1.28706 0 1 3 1 18 0 7 1 0 0 0 0 0 0 0 0 0 0 0 0 0 0 0
2025 2 Syasstro 5.2 W 10 0.96622 4 6 0 1 0 0 0 0 31 0 0 0 0 0 0 0 0 0 0 0 0 0 0
2025 2 Syasstro 7.6 W 10 1.75351 0 4 0 6 17 0 0 0 0 0 0 0 0 0 0 0 0 0 0 0 0 0 0
2025 2 Syasstro 8.6 N 9 1.58075 0 8 0 3 6 0 1 0 5 0 0 0 0 0 0 0 0 0 0 0 0 0 0
2025 2 Syasstro 9.6 W 10 2.44702 0 9 2 1 8 0 0 0 0 0 0 0 0 0 0 1 0 0 0 0 0 0 0 0
2025 2 Syasstro 11.7 W 10 1.46106 0 5 1 1 6 0 0 0 0 0 0 0 0 0 0 0 0 0 0 0 0 0 0
2025 2 Syasstro 12.8 W 10 0.70338 0 6 0 12 12 0 10 0 0 0 0 0 0 0 0 0 0 0 0 0 0 0 0 0
2025 2 Syasstro 14.2 N 10 2.07929 1 4 0 5 0 0 0 0 0 0 0 1 0 0 0 0 0 0 0 0 0 0 0
```

```
2025 2 Volkhov 1.4 W 8 0.64045 0 1 0 6 1 0 0 0 0 0 0 0 0 0 0 0 0 0 0 0 0 0 0 0
2025 2 Volkhov 1.8 W 5 0.51705 0 0 0 26 8 0 0 0 1 0 0 0 0 0 0 0 0 0 0 0 0 0 0 0
2025 2 Volkhov 1.9 S 10 0.82801 0 0 1 0 0 0 0 0 0 0 0 0 0 0 0 0 0 0 0 0 0 0 0 0
2025 2 Volkhov 3.5 W 10 1.08839 0 0 1 1 17 0 0 0 0 0 0 0 0 0 0 0 0 0 0 0 0 0 0 0
2025 2 Volkhov 4.2 W 10 0.75644 0 1 0 1 3 0 0 0 0 0 0 0 0 0 0 0 2 0 0 0 0 0 0 0
2025 2 Volkhov 8.5 N 10 2.15086 0 0 12 1 15 0 0 0 0 0 0 0 0 1 0 0 0 0 0 0 0 0 0 0
2025 2 Volkhov 4.3 S 7 0.58492 0 1 1 0 0 0 1 0 0 0 0 0 0 0 0 0 0 0 0 0 0 0 0 0
2025 2 Volkhov 11.6 N 10 2.21256 0 3 0 10 1 0 0 0 0 0 0 0 0 0 0 0 0 0 0 0 0 0 0 0
2025 2 Volkhov 9.4 S 10 0.88478 0 1 2 1 2 0 0 0 0 0 0 0 0 0 0 0 0 0 0 0 0 0 0 0
2025 2 Volkhov 13.9 N 10 2.44579 0 5 1 2 4 0 3 0 0 0 0 0 0 0 0 0 0 0 0 0 0 0 0 0
2025 2 Volkhov 15.9 N 10 1.95959 0 0 0 2 28 0 0 0 0 0 0 0 0 0 0 0 0 0 0 1 0 0 0 0
2025 2 Volkhov 15.3 S 6 0.46152 0 0 1 0 4 0 1 0 0 0 0 0 0 0 0 0 0 0 0 0 0 0 0 0
```

Data S2
Live weight of third-instar larvae of the autumnal moth, *Epirrita autumnata* fed with foliage of *Betula pubescens* from the surroundings of Volkhov and Syasstroy, 2017.

Explanations of columns:

1. Pollution gradient (Volkhov or Syasstroy);
2. Pollution load (low vs. high);
3. Site code (distance and compass direction from the polluter to the study site; for site co-ordinates consult Table S2);
4. Initial weight (mg);
5. Final weight (mg).

Volkhov	Low	15.9N	8.7	8.8
Volkhov	Low	15.9N	12.0	20.8
Volkhov	Low	15.9N	16.9	21.9
Volkhov	Low	15.9N	27.1	27.2
Volkhov	Low	15.9N	32.2	36.6
Volkhov	High	1.4W	8.9	17.0
Volkhov	High	1.4W	11.6	17.7
Volkhov	High	1.4W	17.6	27.4
Volkhov	High	1.4W	25.7	29.3
Volkhov	High	1.4W	34.4	48.1
Volkhov	High	0.4W	9.0	14.8
Volkhov	High	0.4W	11.5	24.3
Volkhov	High	0.4W	18.3	27.7
Volkhov	High	0.4W	26.8	29.7
Volkhov	High	0.4W	35.5	57.1
Volkhov	Low	15.3S	9.1	17.0
Volkhov	Low	15.3S	11.1	21.0
Volkhov	Low	15.3S	18.8	19.3
Volkhov	Low	15.3S	25.3	28.8
Volkhov	Low	15.3S	35.5	35.6
Syasstroy	Low	12.8W	11.1	19.3
Syasstroy	Low	12.8W	14.6	28.2
Syasstroy	Low	12.8W	23.9	27.0
Syasstroy	Low	12.8W	27.8	32.6
Syasstroy	Low	12.8W	45.1	75.2
Syasstroy	High	2.7W	10.5	27.5
Syasstroy	High	2.7W	15.3	27.9
Syasstroy	High	2.7W	23.8	37.8
Syasstroy	High	2.7W	31.7	40.0
Syasstroy	High	2.7W	37.8	57.3
Syasstroy	High	0.3N	10.0	21.0
Syasstroy	High	0.3N	16.3	29.2
Syasstroy	High	0.3N	22.5	30.9
Syasstroy	High	0.3N	31.7	39.7
Syasstroy	High	0.3N	36.8	54.8
Syasstroy	Low	14.2N	9.9	17.1
Syasstroy	Low	14.2N	16.4	29.4
Syasstroy	Low	14.2N	19.8	35.9
Syasstroy	Low	14.2N	32.1	33.0
Syasstroy	Low	14.2N	36.6	50.9

Data S3
Distribution of downy birch (*Betula pubescens*) leaves by classes of insect damage in the surroundings of Volkhov and Syasstroy, 2007–2025.

Explanations of columns:
1. Year;
2. Census: 1, late Juny – early July; 2, mid August to late September;
3. Pollution gradient (Volkhov or Syasstroy);
4. Pollution load (low vs. high);
5. Site code (distance and compass direction from the polluter to the study site; for site co-ordinates consult Table S2);
6. Number of undamaged leaves;
7. Number of leaves with ≤1% of area consumed or damaged by insects;
8. Number of leaves with 1%–5% of area consumed or damaged by insects;
9. Number of leaves with 5%–25% of area consumed or damaged by insects;
10. Number of leaves with 25%–50% of area consumed or damaged by insects;
11. Number of leaves with 50%–75% of area consumed or damaged by insects;
12. Number of leaves with 75%–100% of area consumed or damaged by insects;
13. Total umber of leaves.

```
2011 1 Volkhov Low 15.9N 19 41 41 17 0 0 2 120
2011 1 Volkhov Low 15.9N 4 19 40 18 3 2 4 90
2011 1 Volkhov Low 15.9N 15 34 33 15 1 2 0 100
2011 1 Volkhov Low 15.9N 2 30 36 17 2 2 0 89
2011 1 Volkhov Low 15.9N 27 47 33 19 4 1 3 134
2011 1 Volkhov High 1.4W 25 21 14 5 0 0 3 68
2011 1 Volkhov High 1.4W 50 35 50 12 0 0 0 147
2011 1 Volkhov High 1.4W 37 54 92 10 0 3 4 200
2011 1 Volkhov High 1.4W 20 17 30 6 0 0 1 74
2011 1 Volkhov High 1.4W 30 17 35 18 2 0 1 103
2011 1 Volkhov High 0.4W 89 20 12 16 2 2 3 144
2011 1 Volkhov High 0.4W 64 16 19 20 3 1 0 123
2011 1 Volkhov High 0.4W 42 4 3 4 1 0 0 54
2011 1 Volkhov High 0.4W 81 15 24 28 9 1 1 159
2011 1 Volkhov High 0.4W 30 9 22 21 2 0 0 84
2011 1 Volkhov Low 13.0S 2 5 12 22 21 1 1 64
2011 1 Volkhov Low 13.0S 2 5 25 27 11 1 1 72
2011 1 Volkhov Low 13.0S 1 18 16 10 1 1 4 51
2011 1 Volkhov Low 13.0S 0 6 16 29 3 0 0 54
2011 1 Volkhov Low 13.0S 4 16 18 22 2 1 1 64
2011 1 Syasstroy Low 12.8W 59 9 4 3 1 1 1 78
2011 1 Syasstroy Low 12.8W 20 12 16 14 3 1 1 67
2011 1 Syasstroy Low 12.8W 39 12 14 10 2 0 0 77
2011 1 Syasstroy Low 12.8W 35 27 12 8 0 0 1 83
2011 1 Syasstroy Low 12.8W 85 14 17 18 3 0 1 138
2011 1 Syasstroy High 2.7W 36 25 10 4 0 0 0 75
2011 1 Syasstroy High 2.7W 83 26 8 5 1 0 0 123
2011 1 Syasstroy High 2.7W 33 8 9 3 4 1 0 58
2011 1 Syasstroy High 2.7W 63 18 2 1 1 0 0 85
2011 1 Syasstroy High 2.7W 49 18 30 12 6 0 0 115
2011 1 Syasstroy High 0.3N 20 39 36 12 2 0 0 109
2011 1 Syasstroy High 0.3N 6 10 22 21 13 3 10 85
2011 1 Syasstroy High 0.3N 65 22 18 11 1 1 2 120
2011 1 Syasstroy High 0.3N 70 10 33 48 11 1 0 173
2011 1 Syasstroy High 0.3N 77 17 10 17 5 0 1 127
2011 1 Syasstroy Low 14.2N 44 9 6 5 1 2 0 67
2011 1 Syasstroy Low 14.2N 18 7 8 23 3 1 4 64
```

2011 1 Syasstroy Low 14.2N 26 12 4 11 7 0 0 60
2011 1 Syasstroy Low 14.2N 14 18 7 4 1 0 10 54
2011 1 Syasstroy Low 14.2N 12 16 7 6 1 0 5 47
2011 2 Volkhov Low 15.9N 6 4 20 41 10 1 1 83
2011 2 Volkhov Low 15.9N 7 7 41 25 4 0 2 86
2011 2 Volkhov Low 15.9N 3 13 41 12 2 0 0 71
2011 2 Volkhov Low 15.9N 2 6 47 27 2 3 7 94
2011 2 Volkhov Low 15.9N 0 22 48 10 3 0 14 97
2011 2 Volkhov High 1.4W 25 28 69 4 0 0 2 128
2011 2 Volkhov High 1.4W 16 23 60 10 1 1 2 113
2011 2 Volkhov High 1.4W 19 18 115 35 2 0 4 193
2011 2 Volkhov High 1.4W 30 39 81 30 1 0 3 184
2011 2 Volkhov High 1.4W 4 17 66 18 1 0 0 106
2011 2 Volkhov High 0.4W 83 15 10 0 0 0 0 108
2011 2 Volkhov High 0.4W 34 30 28 8 1 2 1 104
2011 2 Volkhov High 0.4W 45 13 30 23 4 0 0 115
2011 2 Volkhov High 0.4W 111 21 29 6 2 0 0 169
2011 2 Volkhov High 0.4W 27 10 25 29 15 1 0 107
2011 2 Volkhov Low 13.0S 12 35 62 9 3 1 5 127
2011 2 Volkhov Low 13.0S 17 15 59 23 9 5 5 133
2011 2 Volkhov Low 13.0S 14 9 41 38 7 0 8 117
2011 2 Volkhov Low 13.0S 12 18 43 20 2 1 2 98
2011 2 Volkhov Low 13.0S 3 6 41 28 1 1 10 90
2011 2 Syasstroy Low 12.8W 8 13 25 20 2 0 0 68
2011 2 Syasstroy Low 12.8W 3 4 22 43 4 0 0 76
2011 2 Syasstroy Low 12.8W 25 4 28 10 0 0 0 67
2011 2 Syasstroy Low 12.8W 24 24 54 37 3 0 0 142
2011 2 Syasstroy Low 12.8W 43 15 8 14 5 2 0 87
2011 2 Syasstroy High 2.7W 84 31 80 25 4 0 0 224
2011 2 Syasstroy High 2.7W 6 13 28 21 3 2 0 73
2011 2 Syasstroy High 2.7W 32 26 79 21 4 0 0 162
2011 2 Syasstroy High 2.7W 34 27 32 14 2 1 2 112
2011 2 Syasstroy High 2.7W 35 18 43 37 10 7 1 151
2011 2 Syasstroy High 0.3N 16 13 43 21 9 0 0 102
2011 2 Syasstroy High 0.3N 43 38 60 4 0 0 0 145
2011 2 Syasstroy High 0.3N 18 21 37 29 11 6 5 127
2011 2 Syasstroy High 0.3N 9 14 45 11 3 0 0 82
2011 2 Syasstroy High 0.3N 11 9 59 27 5 1 0 112
2011 2 Syasstroy Low 14.2N 2 9 22 30 10 11 10 94
2011 2 Syasstroy Low 14.2N 9 4 13 25 10 3 9 73
2011 2 Syasstroy Low 14.2N 18 3 23 20 3 0 1 68
2011 2 Syasstroy Low 14.2N 23 34 49 25 4 0 3 138
2011 2 Syasstroy Low 14.2N 5 9 27 22 12 10 7 92
2017 1 Volkhov Low 15.9N 30 44 35 14 1 0 1 125
2017 1 Volkhov Low 15.9N 50 53 23 10 0 0 1 137
2017 1 Volkhov Low 15.9N 30 43 21 7 1 2 2 106
2017 1 Volkhov Low 15.9N 24 47 19 6 2 0 1 99
2017 1 Volkhov Low 15.9N 4 58 23 10 0 1 1 97
2017 1 Volkhov High 1.4W 57 26 15 7 2 0 0 107
2017 1 Volkhov High 1.4W 41 45 11 4 1 0 2 104
2017 1 Volkhov High 1.4W 74 33 4 1 0 0 0 112
2017 1 Volkhov High 1.4W 62 25 15 3 0 0 0 105
2017 1 Volkhov High 1.4W 106 5 2 0 0 0 0 113
2017 1 Volkhov High 1.8W 54 42 10 2 0 0 0 108
2017 1 Volkhov High 1.8W 17 35 33 19 3 0 0 107
2017 1 Volkhov High 1.8W 7 106 23 3 1 0 0 140
2017 1 Volkhov High 1.8W 9 75 30 11 1 0 2 128
2017 1 Volkhov High 1.8W . . . .
2017 1 Volkhov Low 15.3S 91 21 5 4 1 0 0 122
2017 1 Volkhov Low 15.3S 46 35 9 8 2 1 1 102
2017 1 Volkhov Low 15.3S 72 26 16 6 2 0 0 122

2017	1	Volkhov	Low	15.3S	57	16	15	7	1	5	13	114
2017	1	Volkhov	Low	15.3S	92	20	4	3	0	0	2	121
2017	1	Syasstroy	Low	12.8W	94	16	9	2	0	0	0	121
2017	1	Syasstroy	Low	12.8W	89	21	8	1	0	2	0	121
2017	1	Syasstroy	Low	12.8W	63	7	2	0	0	0	0	72
2017	1	Syasstroy	Low	12.8W	71	27	18	11	0	3	1	131
2017	1	Syasstroy	Low	12.8W	57	24	11	2	0	3	5	102
2017	1	Syasstroy	High	2.7W	29	25	17	12	3	0	0	86
2017	1	Syasstroy	High	2.7W	20	29	9	13	5	2	4	82
2017	1	Syasstroy	High	2.7W	19	28	20	18	4	4	0	93
2017	1	Syasstroy	High	2.7W	22	36	19	27	4	3	0	111
2017	1	Syasstroy	High	2.7W	31	25	9	5	0	0	3	73
2017	1	Syasstroy	High	0.3N	103	25	9	0	0	0	0	137
2017	1	Syasstroy	High	0.3N	193	42	7	1	1	0	0	244
2017	1	Syasstroy	High	0.3N	70	76	12	12	0	2	0	172
2017	1	Syasstroy	High	0.3N	169	10	5	12	2	0	0	198
2017	1	Syasstroy	High	0.3N	130	36	18	7	1	1	0	193
2017	1	Syasstroy	Low	14.2N	49	34	50	52	8	2	0	195
2017	1	Syasstroy	Low	14.2N	106	42	22	11	2	0	4	187
2017	1	Syasstroy	Low	14.2N	87	53	20	11	0	0	1	172
2017	1	Syasstroy	Low	14.2N	64	36	9	13	5	0	4	131
2017	1	Syasstroy	Low	14.2N	51	39	11	16	3	0	2	122
2017	2	Volkhov	Low	15.9N	4	14	43	25	0	0	1	87
2017	2	Volkhov	Low	15.9N	1	11	53	30	4	3	3	105
2017	2	Volkhov	Low	15.9N	1	3	21	35	0	0	8	68
2017	2	Volkhov	Low	15.9N	.	.	.	.	.	.	.	.
2017	2	Volkhov	Low	15.9N	.	.	.	.	.	.	.	.
2017	2	Volkhov	High	1.4W	11	31	39	22	0	0	0	103
2017	2	Volkhov	High	1.4W	13	38	34	11	3	1	2	102
2017	2	Volkhov	High	1.4W	14	59	37	12	1	1	2	126
2017	2	Volkhov	High	1.4W	5	72	40	5	0	0	0	122
2017	2	Volkhov	High	1.4W	18	48	26	7	0	0	2	101
2017	2	Volkhov	High	1.8W	1	24	27	9	4	1	2	68
2017	2	Volkhov	High	1.8W	6	55	18	6	0	0	1	86
2017	2	Volkhov	High	1.8W	6	34	49	12	2	0	2	105
2017	2	Volkhov	High	1.8W	8	61	45	6	1	0	1	122
2017	2	Volkhov	High	1.8W	1	44	48	6	0	0	1	100
2017	2	Volkhov	Low	15.3S	8	23	33	34	6	2	24	130
2017	2	Volkhov	Low	15.3S	1	10	17	21	4	5	43	101
2017	2	Volkhov	Low	15.3S	19	35	43	22	1	0	1	121
2017	2	Volkhov	Low	15.3S	3	31	33	19	7	0	2	95
2017	2	Volkhov	Low	15.3S	5	15	32	22	6	13	19	112
2017	2	Syasstroy	Low	12.8W	6	10	19	11	3	1	0	50
2017	2	Syasstroy	Low	12.8W	2	29	9	7	2	0	1	50
2017	2	Syasstroy	Low	12.8W	6	15	5	12	6	3	3	50
2017	2	Syasstroy	Low	12.8W	12	19	45	53	4	4	1	138
2017	2	Syasstroy	Low	12.8W	6	61	31	20	2	0	3	123
2017	2	Syasstroy	High	2.7W	9	31	49	19	1	0	3	112
2017	2	Syasstroy	High	2.7W	17	41	26	20	7	1	7	119
2017	2	Syasstroy	High	2.7W	16	37	19	36	13	3	10	134
2017	2	Syasstroy	High	2.7W	9	27	28	32	4	1	1	102
2017	2	Syasstroy	High	2.7W	7	50	39	22	26	6	3	153
2017	2	Syasstroy	High	0.3N	17	25	42	10	2	0	0	96
2017	2	Syasstroy	High	0.3N	2	29	66	15	0	0	0	112
2017	2	Syasstroy	High	0.3N	17	38	41	7	0	0	2	105
2017	2	Syasstroy	High	0.3N	21	27	49	21	3	2	2	125
2017	2	Syasstroy	High	0.3N	17	28	57	9	0	0	0	111
2017	2	Syasstroy	Low	14.2N	16	1	32	30	8	4	8	99
2017	2	Syasstroy	Low	14.2N	15	35	32	7	4	0	6	99
2017	2	Syasstroy	Low	14.2N	8	37	28	29	6	1	1	110
2017	2	Syasstroy	Low	14.2N	0	12	41	39	4	4	8	108

```
2017 2 Syasstroy Low 14.2N 14 30 39 8 1 3 10 105
2025 1 Volkhov Low 15.9N 16 30 17 2 0 2 4 71
2025 1 Volkhov Low 15.9N 5 26 18 8 2 6 10 75
2025 1 Volkhov Low 15.9N 4 14 5 0 0 5 7 35
2025 1 Volkhov Low 15.9N 8 23 6 9 1 3 6 56
2025 1 Volkhov Low 15.9N 11 20 27 23 14 1 0 96
2025 1 Volkhov High 1.8W 16 15 51 17 3 1 5 108
2025 1 Volkhov High 1.8W 6 22 38 6 0 5 16 93
2025 1 Volkhov High 1.8W 2 10 37 25 2 8 5 89
2025 1 Volkhov High 1.8W 2 12 27 10 3 1 0 55
2025 1 Volkhov High 1.8W 4 14 23 25 3 1 3 73
2025 1 Volkhov High 1.4W 19 8 4 0 0 1 1 33
2025 1 Volkhov High 1.4W 46 30 15 2 0 0 1 94
2025 1 Volkhov High 1.4W 10 62 24 21 0 3 1 121
2025 1 Volkhov High 1.4W 40 79 31 16 1 3 6 176
2025 1 Volkhov High 1.4W 27 47 7 0 0 6 6 93
2025 1 Volkhov Low 15.3S 2 15 12 5 0 9 10 53
2025 1 Volkhov Low 15.3S 7 23 9 1 0 7 18 65
2025 1 Volkhov Low 15.3S 8 18 27 12 1 0 0 66
2025 1 Volkhov Low 15.3S 25 16 13 7 3 0 3 67
2025 1 Volkhov Low 15.3S 9 19 13 10 2 4 3 60
2025 1 Syasstroy Low 12.8W 4 17 12 10 1 3 2 49
2025 1 Syasstroy Low 12.8W 21 6 2 2 0 0 0 31
2025 1 Syasstroy Low 12.8W 11 27 8 5 2 4 6 63
2025 1 Syasstroy Low 12.8W 5 9 8 4 0 8 13 47
2025 1 Syasstroy Low 12.8W 3 14 4 0 0 6 8 35
2025 1 Syasstroy High 2.7W 12 26 13 4 1 1 2 59
2025 1 Syasstroy High 2.7W 24 19 11 9 1 1 0 65
2025 1 Syasstroy High 2.7W 5 22 17 4 0 2 3 53
2025 1 Syasstroy High 2.7W 26 36 5 4 0 2 6 79
2025 1 Syasstroy High 2.7W 11 42 24 18 1 2 6 104
2025 1 Syasstroy High 0.3N 12 11 28 18 0 0 0 69
2025 1 Syasstroy High 0.3N 60 7 14 12 1 1 0 95
2025 1 Syasstroy High 0.3N 35 12 3 5 0 1 1 57
2025 1 Syasstroy High 0.3N 52 17 5 2 0 0 0 76
2025 1 Syasstroy High 0.3N 25 13 15 13 0 0 2 68
2025 1 Syasstroy Low 14.2N 2 16 21 13 1 1 1 55
2025 1 Syasstroy Low 14.2N 3 11 12 6 1 1 1 35
2025 1 Syasstroy Low 14.2N 17 8 2 5 0 0 0 32
2025 1 Syasstroy Low 14.2N 29 16 8 5 3 0 0 61
2025 1 Syasstroy Low 14.2N 8 42 11 4 0 3 0 68
2025 2 Volkhov Low 15.9N 12 17 17 20 3 9 20 98
2025 2 Volkhov Low 15.9N 3 10 39 29 1 0 2 84
2025 2 Volkhov Low 15.9N 0 3 14 28 3 9 14 71
2025 2 Volkhov Low 15.9N 0 4 5 15 13 14 18 69
2025 2 Volkhov Low 15.9N 2 12 9 15 4 8 17 67
2025 2 Volkhov High 1.8W 10 9 15 13 0 2 2 51
2025 2 Volkhov High 1.8W 13 28 12 12 2 0 0 67
2025 2 Volkhov High 1.8W 6 18 23 16 2 0 4 69
2025 2 Volkhov High 1.8W 2 8 17 14 1 1 2 45
2025 2 Volkhov High 1.8W 1 3 3 11 2 0 1 21
2025 2 Volkhov High 1.4W 24 21 18 13 0 0 0 76
2025 2 Volkhov High 1.4W 9 22 11 7 0 0 1 50
2025 2 Volkhov High 1.4W 26 25 5 4 0 1 1 62
2025 2 Volkhov High 1.4W 14 8 6 1 0 0 0 29
2025 2 Volkhov High 1.4W 6 13 10 8 0 0 0 37
2025 2 Volkhov Low 15.3S 27 10 10 8 0 1 1 57
2025 2 Volkhov Low 15.3S 8 10 8 2 1 0 0 29
2025 2 Volkhov Low 15.3S 9 9 8 18 2 1 1 48
2025 2 Volkhov Low 15.3S 8 12 8 13 2 6 2 51
2025 2 Volkhov Low 15.3S . . . . . . . .
```

```
2025 2 Syasstroy Low 12.8W 13 11 17 25 0 3 4 73
2025 2 Syasstroy Low 12.8W 9 16 12 26 2 6 4 75
2025 2 Syasstroy Low 12.8W 8 12 16 9 1 2 2 50
2025 2 Syasstroy Low 12.8W 11 14 21 21 1 4 8 80
2025 2 Syasstroy Low 12.8W 1 2 10 40 1 3 2 59
2025 2 Syasstroy High 2.7W 22 28 16 17 1 2 1 87
2025 2 Syasstroy High 2.7W 27 14 8 7 0 1 1 58
2025 2 Syasstroy High 2.7W 26 24 7 9 2 0 0 68
2025 2 Syasstroy High 2.7W 84 29 17 5 2 1 0 138
2025 2 Syasstroy High 2.7W 78 17 11 14 0 1 0 121
2025 2 Syasstroy High 0.3N 101 18 13 23 6 4 5 170
2025 2 Syasstroy High 0.3N 90 12 11 3 0 0 1 117
2025 2 Syasstroy High 0.3N 92 28 12 17 2 0 0 151
2025 2 Syasstroy High 0.3N 77 34 24 9 1 0 2 147
2025 2 Syasstroy High 0.3N 74 10 16 9 0 1 0 110
2025 2 Syasstroy Low 14.2N 16 17 18 10 2 0 6 69
2025 2 Syasstroy Low 14.2N 15 12 15 11 1 3 17 74
2025 2 Syasstroy Low 14.2N 17 19 15 12 2 0 8 73
2025 2 Syasstroy Low 14.2N 25 15 7 10 3 0 3 63
2025 2 Syasstroy Low 14.2N 41 33 18 19 1 2 4  118
```
